# Transcriptome-wide splicing analysis reveals HuR-regulated post-transcriptional programs in osteocytes

**DOI:** 10.1016/j.isci.2026.116529

**Published:** 2026-08-10

**Authors:** Ziqiu Fan, Aseel Marahleh, Hideki Kitaura, Jiayi Ren, Abdulrahman Mousa, Fumitoshi Ohori, Kuniyasu Niizuma, Sherif Rashad, Hiroyasu Kanetaka

**Affiliations:** 1 Orthodontics and Dentofacial Orthopedics Department, Graduate School of Dentistry, Tohoku University, Sendai 980-8575, Japan; 2 Frontier Research Institute for Interdisciplinary Sciences, Tohoku University, Sendai 980-8578, Japan; 3 Center for Environmental Response and Aging, Institute of Development, Aging and Cancer, Tohoku University, Sendai 980-8575, Japan; 4 Department of Neurosurgical Engineering, Graduate School of Biomedical Engineering, Tohoku University, Sendai 980-8575, Japan; 5 Department of Translational Neuroscience, Graduate School of Medicine, Tohoku University, Sendai 980-8575, Japan; 6 Department of Biomedical Engineering, Graduate School of Medicine, Tohoku University, Sendai 980-8575, Japan

**Keywords:** HuR/Elavl1, RNA-binding proteins, alternative splicing, hyperglycemia, osteocytes, TXNIP, translation, mTOR

## Abstract

Post-transcriptional gene regulation is central to maintaining cellular homeostasis. Among its mechanisms, alternative splicing (AS) fine-tunes cellular adaptation to stress. This study employed an approach combining RNA splicing analysis with RNA-binding protein (RBP) motif enrichment in primary osteocytes cultured in high-glucose conditions. Our analysis identified the RBP human antigen R (HuR) as a top candidate associated with AS regulation. Loss of HuR reshaped the transcriptome through gene expression and splicing changes, converging on two major pathways: stress response and translational control. Functional validation revealed that HuR depletion heightened oxidative stress, impaired mitochondrial function, and rewired key translational signals, while preserving global protein output. Mechanistically, we identified TXNIP mRNA-protein uncoupling following HuR knockdown (KD), characterized by elevated mRNA but reduced protein expression. Collectively, these findings support HuR’s role as a key post-transcriptional regulator of osteocyte metabolic adaptation under high-glucose stress, with potential implications for hyperglycemic bone fragility.

## Highlights

• High glucose induces extensive splicing changes in osteocytes
• HuR-binding motifs are enriched around spliced transcripts in high glucose
• HuR KD rewires translation signaling pathways (MNK and mTOR)
• HuR uncouples TXNIP mRNA-protein levels post-transcriptionally

## Graphical abstract

**Figure:**
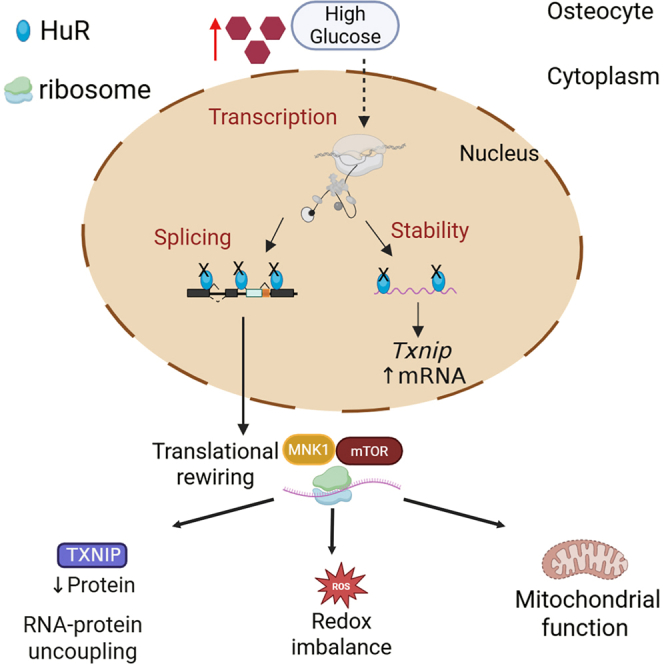

## Teaser

Natural sciences; Biological sciences; Genetics; Cell biology

## Introduction

Post-transcriptional gene regulation controls RNA splicing, modification, localization, stability, translation, and decay, generating transcript diversity and shaping proteomic output.^1^^,^^2^^,^^3^^,^^4^^,^^5^^,^^6^ These processes are central to stress adaptation, helping to promote cell survival.^7^^,^^8^ RNA-binding proteins (RBPs) orchestrate these events by interacting with structured RNA motifs to control nearly every aspect of RNA metabolism.^9^^,^^10^^,^^11^^,^^12^ RBPs contribute to tissue-specific disease phenotypes, often reflecting the expression pattern of their RNA targets and cofactors as well as the RBP’s own abundance and activity level.^13^ Dysregulation of RBPs or their targets contributes to diverse pathologies, including neurodegenerative, muscular, autoimmune, and metabolic diseases, highlighting the critical role of RNA-RBP networks in maintaining cellular homeostasis.^9^^,^^13^^,^^14^

HuR (human antigen R), encoded by *Elavl1* (embryonic lethal abnormal vision-like 1), is a ubiquitously expressed RBP, a classic RBP, possessing RNA recognition motifs and serving as a key modulator of post-transcriptional gene regulation.^15^ It has been extensively studied as a cancer drug target,^16^ and its global deletion has been shown to be embryonically lethal.^17^ Conditional knockouts of HuR experienced impaired postnatal formation of skeletal, immune, and other tissues.^18^^,^^19^^,^^20^^,^^21^ Beyond development, tissue-specific knockouts disrupted metabolic homeostasis, where glucose and lipid metabolism were impaired linking it to obesity and insulin resistance.^22^^,^^23^^,^^24^

Studies on skeletal HuR expression and its role in bone homeostasis are context dependent. In bone, an ovariectomy-induced bone loss model had reduced HuR expression, whereas overexpression alleviated bone loss and silencing impaired osteoblast differentiation.^25^ In contrast, diabetic mouse models showed increased HuR mRNA levels, and its knockdown (KD) improved glucose metabolism and bone microarchitecture.^26^ Similarly, in MC3T3-E1 cells exposed to high glucose (HG), HuR expression increased, and its silencing reduced apoptosis while promoting osteogenic differentiation.^26^ These studies demonstrate that HuR expression and its effects are tissue and context dependent.

Osteocytes, the predominant bone cell type, are post-mitotic mechanosensors maintaining bone remodeling through orchestrating resorption and formation. They are endocrine and signaling cells that maintain skeletal homeostasis.^27^^,^^28^^,^^29^^,^^30^ To date, post-transcriptional RNA regulation in osteocytes and the RBPs regulating it remain largely uncharted. Here, we performed an unbiased RBP motif enrichment analysis on differentially spliced mRNAs from RNA sequencing (RNA-seq) data of primary osteocytes from *Dmp1-Topaz* transgenic mice cultured under HG conditions. This approach enabled us to map the alternative splicing (AS) programs regulated in osteocytes and map the RBPs that regulate them. HuR emerged as a candidate regulator during HG stress. Using HuR KD, we examined both differential gene expression and splicing alterations. HuR depletion reshaped stress-response and translational networks, impaired mitochondrial function, and altered redox homeostasis, supporting a role for HuR in osteocyte adaptation to metabolic stress. We further identified thioredoxin-interacting protein mRNA (*Txnip*) as a HuR-associated target exhibiting mRNA-protein uncoupling following HuR depletion. Although HuR interacted with *Txnip* mRNA and its depletion increased *Txnip* mRNA abundance and stability, protein levels were reduced. Collectively, these findings define HuR as an important post-transcriptional regulator in osteocytes and highlight translational control as a critical layer of metabolic stress adaptation in bone.

## Results

### Splicing-driven RBP motif analysis identifies HuR as a top regulator in osteocytes under HG stress

**Figure 1:**
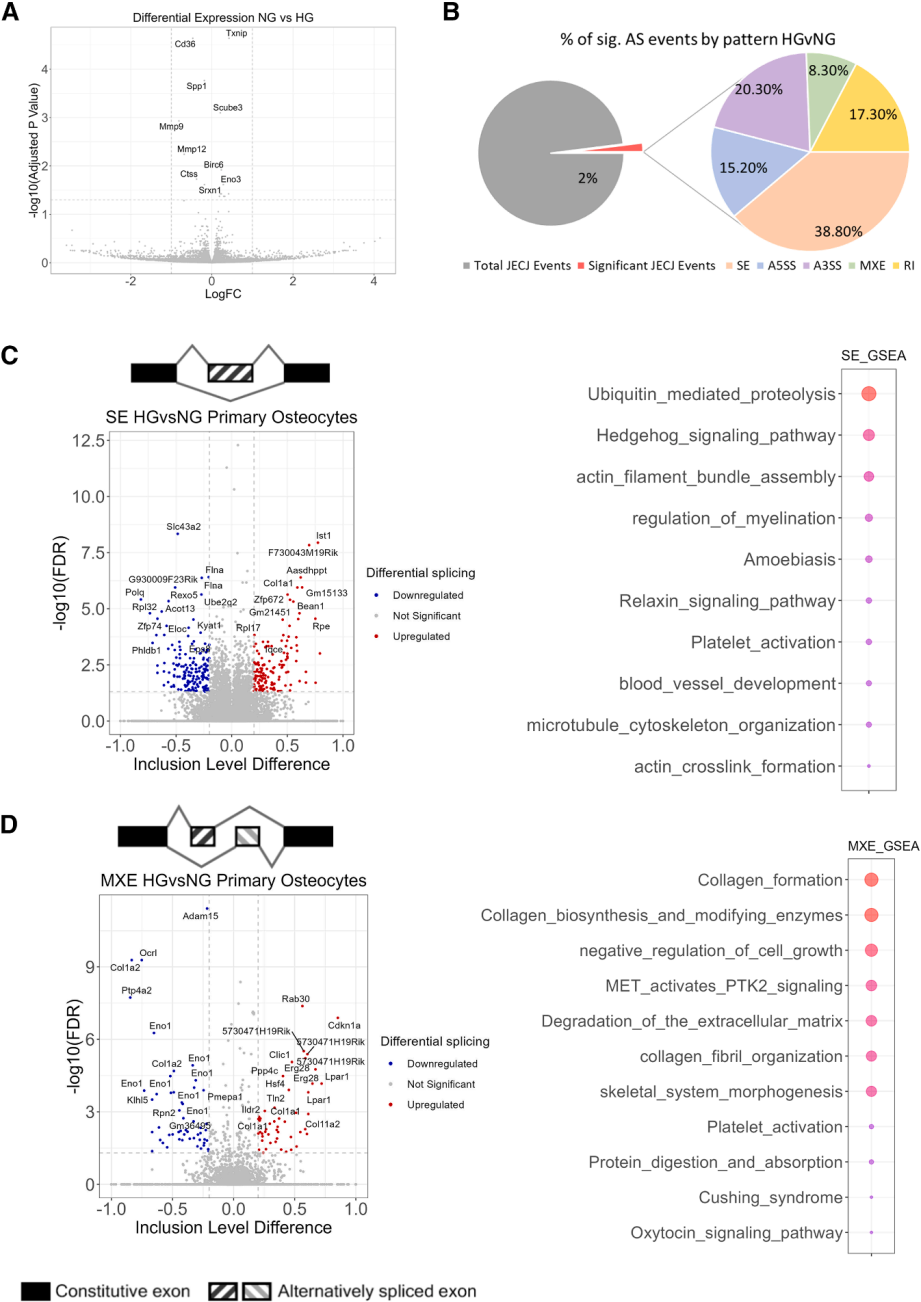
High glucose induces minimal differential gene expression and extensive AS changes in primary osteocytes (A) The volcano plot comparing the log2 FC and −log10 adjusted *p* value of RNA-seq data in primary osteocytes with genes showing the highest changes labeled. (B) The parent pie chart shows significant AS events (2%) of the total events at junction and exon body counts (JCECs), and the secondary pie chart shows the percentage of significant AS events of each type (SE, A5SS, A3SS, MXE, and RI) from total significant events in primary osteocytes. (C and D) Volcano plots and overrepresentation analysis of DSGs around AS events showing inclusion level (ΔѰ) difference comparing 5.5 and 25 mM glucose at FDR < 0.05 and 0.2 < ΔѰ < −0.2 in primary osteocytes. Overrepresentation analysis was done through the eVITTA web-based toolbox. All analysis was done on *n* = 3. Also, see Table S6.

Primary osteocytes obtained from *Dmp1-Topaz* mice were cultured in HG or normal glucose (NG) for 72 h. RNA-seq analysis showed minimal gene expression changes (Figure 1A). rMATS analysis of the 5 canonical splicing patterns—skipped exon (SE), mutually exclusive exons (MXEs), retained introns (RIs), alternative 5′ splice site (A5SS), and alternative 3′ splice site (A3SS)—revealed significant changes (false discovery rate [FDR] <0.05, |ΔΨ| > 0.2) in ∼2% of total events, predominantly SE and A3SS (Figure 1B).^31^ GO Biological Processes (GOBPs) and pathway enrichment analysis of AS showed that different AS programs contribute differently to osteocyte biology. Specifically, SE differentially spliced genes (DSGs) revealed enrichment in cytoskeletal remodeling, protein regulation via ubiquitination, and several pathways involved in matrix maintenance and repair (Figure 1C and Table S1). MXE DSGs were enriched in pathways involved in collagen synthesis and organization, skeletal tissue development, and pathways involved in cell growth and regulation of fluid balance (Figure 1D and Table S1). A5SS DSGs were predominantly enriched in pathways related to post-transcriptional and translational regulation, including ribosome targeting, rRNA processing, and quality control of mRNA translation. A3SS DSGs were involved in RNA splicing, several signaling pathways involved in cell growth and differentiation, and parathyroid hormone action. DSGs in RI showed cell-cycle-related terms, ribosome and translation regulation, as well as ubiquitination (Figures S1A–S1C and Table S1).

**Figure 2:**
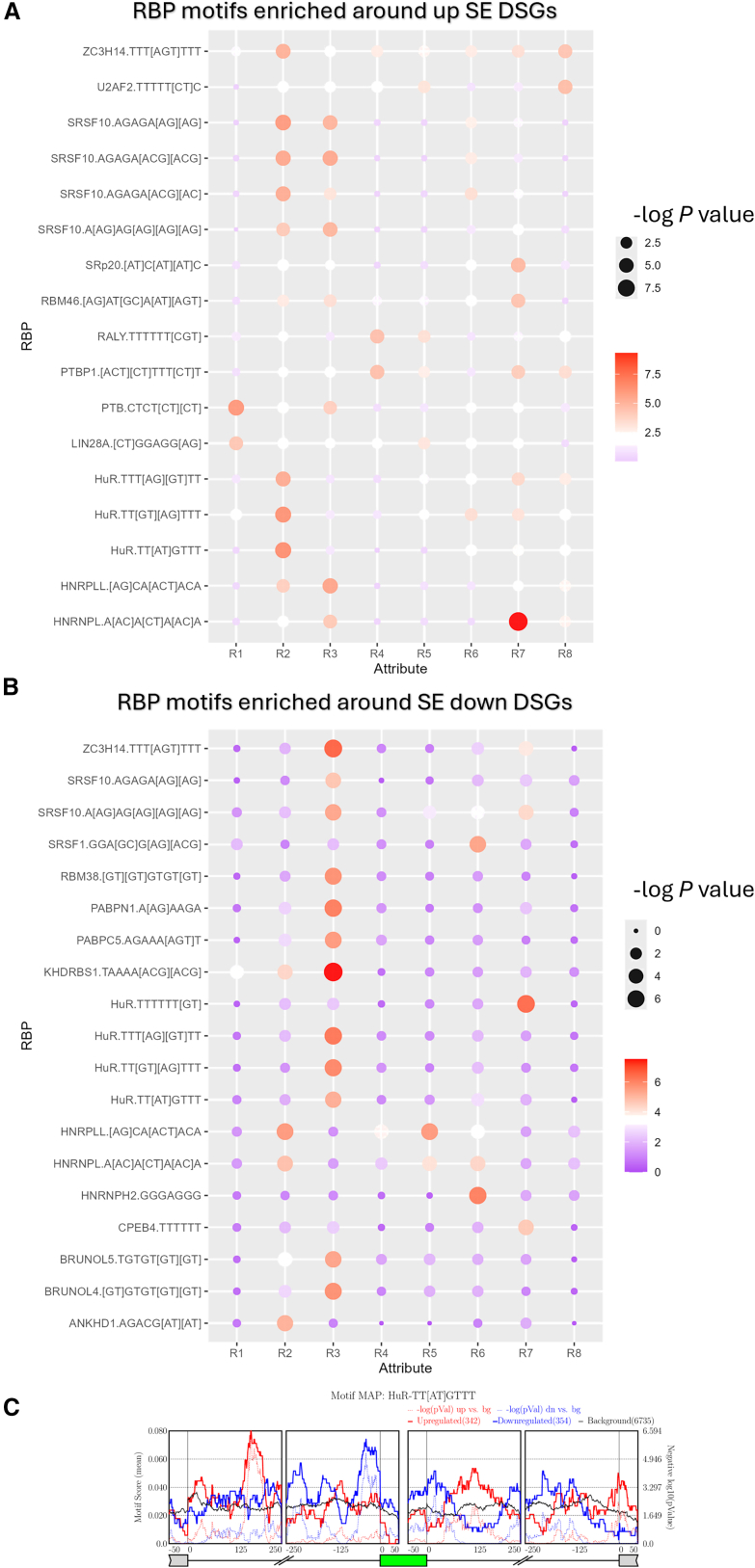
RBP motifs significantly enriched around AS events (A and B) The *x* axis (attribute) represents the position (R) of each splicing event in 5′ → 3′. RBP-binding motifs are described in the *y* axis. The images show skipped exons (SEs) and RBP motifs around up- and downregulated DSGs, and bubble size and color denote the −log10 *p* value. (C) Example positional distribution map of the HuR-binding motif around target SEs. Positional distribution maps were generated using rMAPS2. Intronic regions are 250-bp flanking sequences, and exon regions are 50-bp sequences from the start or end of the exon where the RBP binds around the spliced exon (green) and constitutive exons (gray). The solid red and blue lines are the motif enrichment scores, and the dotted lines are the *p* values in up and down-enriched motifs, respectively. The black line refers to background RBP distribution. Also, see Table S7.

Since these AS changes suggested altered post-transcriptional control, we next interrogated RBP motif enrichment using rMAPS2.^32^ We identified significantly enriched motifs (*p* < 0.05) across all five AS event types and ranked RBPs based on motif enrichment relative to background. For upregulated splice events, HNRNPL and HuR were the top enriched around skipped exon (SE) (Figure 2A and Table S2). In alternate exon usage (MXE), ZC3H14 and HuR were most enriched. HNRNPL and HuR were enriched in A5SS. FUS and HNRNPH2 were enriched around A3SS, and we observed no enriched RBP motifs around intron retention events at a cutoff value of *p* < 0.05 (Figures S2A–S2D and Table S2). For downregulated splice events, KHDRBS1 and ZC3H14 were the top enriched in SE (Figure 2B). ZC3H14 and Tra2-beta were the top enriched in MXE. In A5SS, HNRPLL and HuR showed the highest enrichment. In A3SS, RBM47 and SRp20 were the most enriched. RBMS3 and PABPN1 were the top enriched in RI (Figures S3A–S3D and Table S2).

**Figure 3:**
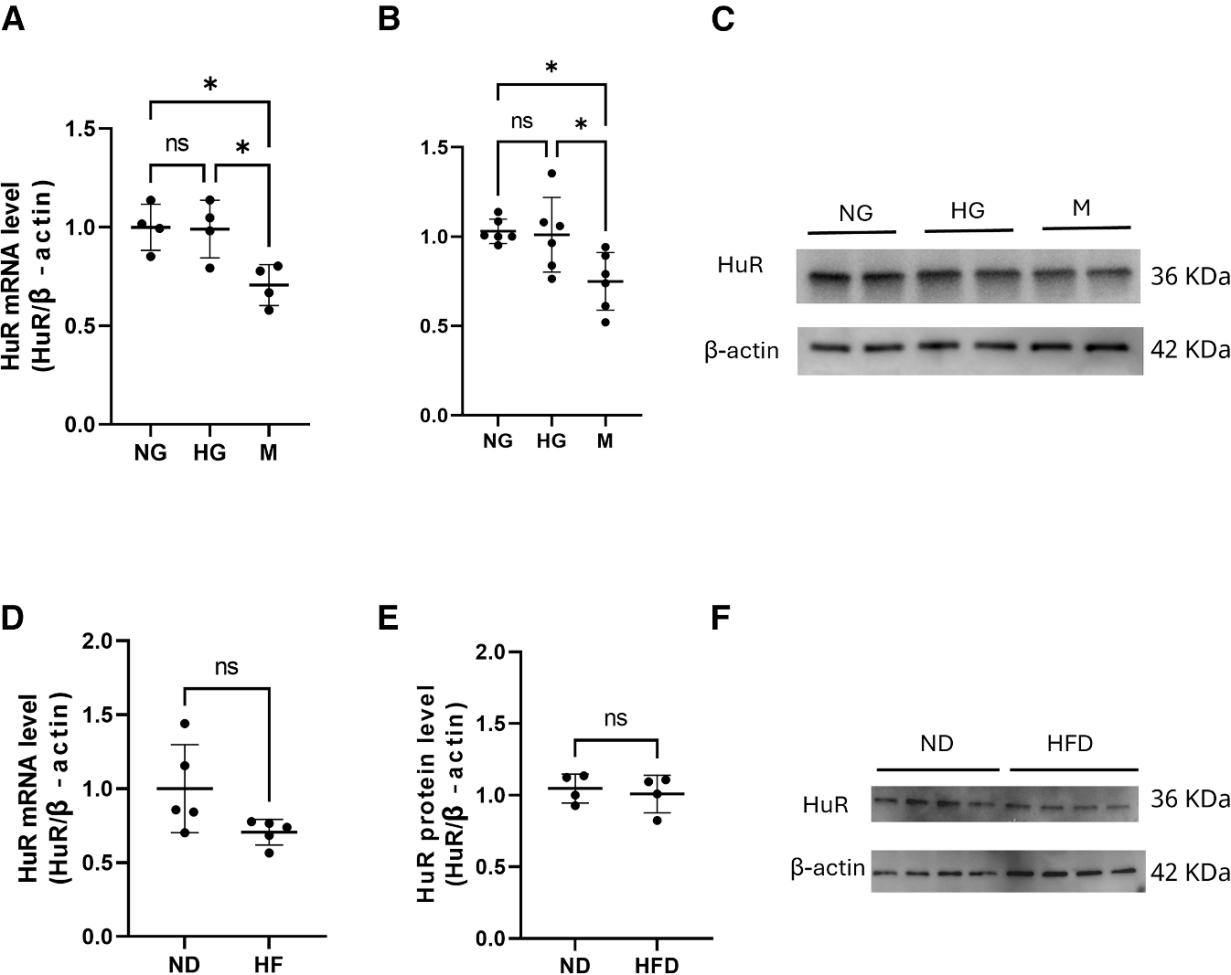
HuR expression is stable in MLO-Y4 cells cultured in HG and in the long bones of HFD-fed mice (A) *Elavl1*/HuR mRNA level in MLO-Y4 cells under normal glucose (NG), high glucose (HG), and osmotic control mannitol (M) relative to β-actin. Data are presented as mean ± SD, *n* = 4. Statistical analysis was performed using one-way ANOVA followed by Tukey’s post hoc test, ∗*p* < 0.05. (B and C) HuR protein level in MLO-Y4 cells in NG, HG, and osmotic control M relative to β-actin. Data are presented as mean ± SD, *n* = 7. Statistical analysis was performed using one-way ANOVA followed by Tukey’s post hoc test. ∗*p* < 0.05. (D) *Elavl1*/HuR mRNA level in the long bones of high-fat diet (HFD)- and normal diet (ND)-fed mice relative to β-actin. Data are presented as mean ± SD, *n* = 5. Statistical analysis was performed using Student’s *t* test. (E and F) HuR protein level in the long bones of HFD- and ND-fed mice relative to β-actin. Data are presented as mean ± SD, *n* = 4. Statistical analysis was performed using Student’s *t* test.

The analysis showed that several HuR motifs were enriched in upregulated splice events. Figure 2C shows an example of HuR-binding motifs around SE differentially spliced exons. Our bioinformatics analysis points to enriched HuR motifs in splicing events, suggesting that HuR may contribute to AS regulation. Therefore, we measured HuR mRNA and protein levels in MLO-Y4 cells exposed to HG, NG, and mannitol (M) osmotic control. mRNA and protein expressions were unaltered as measured by qPCR and western blot, respectively (Figures 3A–3C). M-cultured MLO-Y4 cells exhibited reduced mRNA expression and protein levels. Similarly, HuR mRNA and protein expressions were unaltered in the long bones of hyperglycemic mice that were fed a moderately high-fat diet (HFD) for 16 weeks (Figures 3D–3F and S4).

### HuR KD reveals transcriptomic and phenotypic changes in MLO-Y4 cells

**Figure 4:**
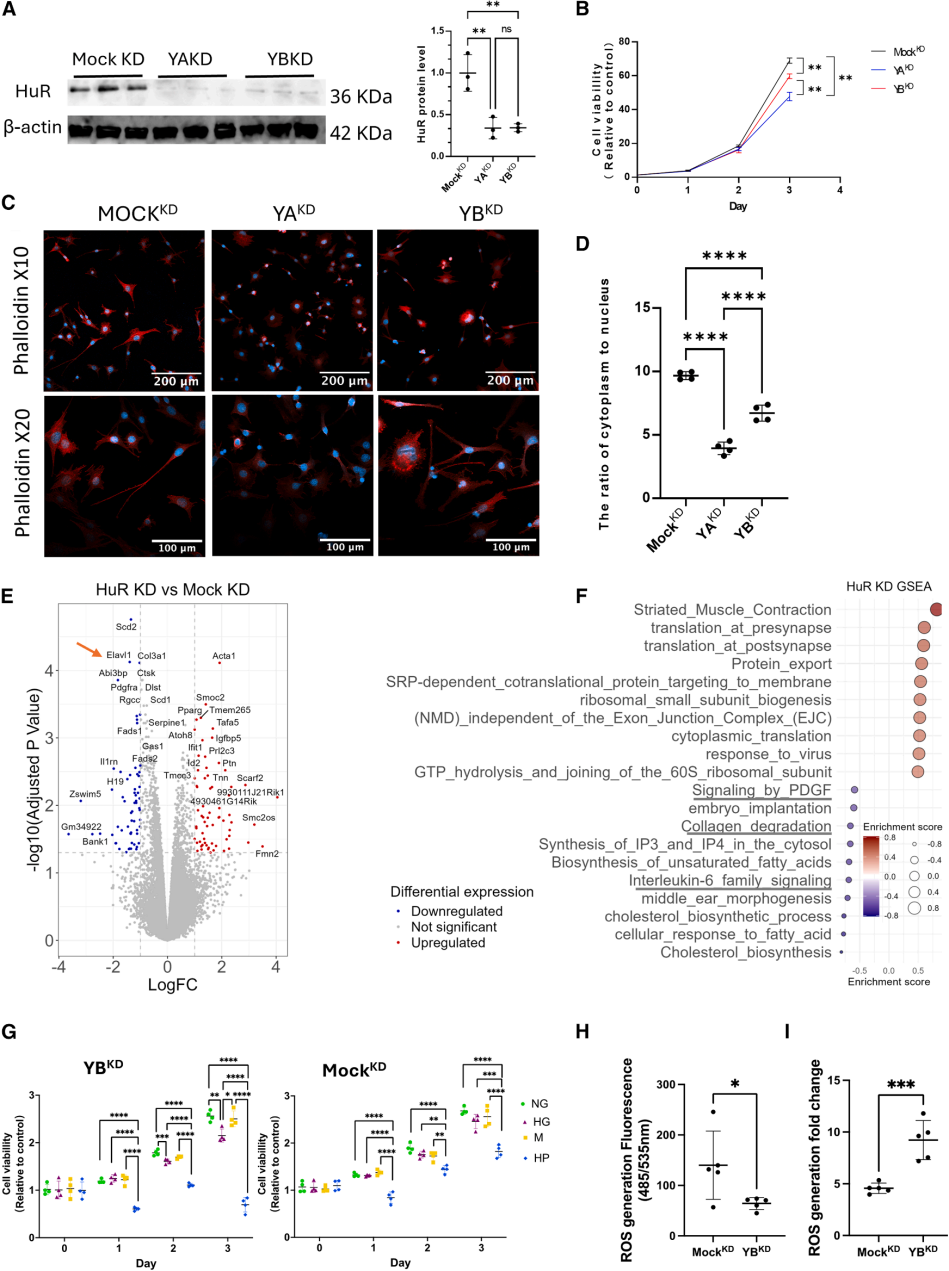
HuR KD reshapes MLO-Y4 cell line phenotype and increases susceptibility to HG injury (A) Western blot analysis of HuR protein levels relative to β-actin after shRNA-mediated KD in MLO-Y4 cells. Data are presented as mean ± SD, *n* = 3. Statistical analysis was performed using one-way ANOVA followed by Tukey’s post hoc test. ∗∗*p* < 0.01. (B) CCK cell viability assay. Data are presented as mean ± SD, *n* = 4. Statistical analysis was performed using one-way ANOVA followed by Tukey’s post hoc test. ∗∗*p* < 0.01. (C) Confocal microscopic images of phalloidin staining. Scale bars: 200 μm for the 10× image and 100 μm for the 20× image. (D) Cytoplasm to nucleus ratio. The mean of 5 analyzed regions (corners and center) of a 35 mm glass-bottom dish was used. Data are presented as mean ± SD, *n* = 4. Statistical analysis was performed using one-way ANOVA followed by Tukey’s post hoc test. ∗∗∗∗*p* < 0.0001. (E) Volcano plot comparing the log2 FC and −log10 adjusted *p* value of RNA-seq data (DEGs) after YB^KD^ in MLO-Y4 cells, red arrow = HuR. *n* = 3. (F) Gene set enrichment analysis using eVITTA toolbox after YB^KD^ of DEGs at |LogFC| ≥ 1 and −log10 adjusted *p* < 0.05. Underlined pathways are most directly relevant to skeletal biology. (G) Cell viability assay in YB^KD^ and Mock^KD^ cells in 5.5 mM glucose (NG), 25 mM glucose (HG), mannitol (M), and H_2_O_2_-stimulated cells. Data are presented as mean ± SD, *n* = 4. Statistical analysis was performed using one-way ANOVA followed by Tukey’s post hoc test. ∗*p* < 0.05, ∗∗*p* < 0.01, ∗∗∗*p* < 0.001, ∗∗∗∗*p* < 0.0001. (H) Reactive oxygen species (ROS) measurement at baseline culture conditions. Data are presented as mean ± SD, *n* = 5 per group. Statistical analysis was performed using Student’s *t* test. ∗*p* < 0.05. (I) ROS measurement after TBHP treatment. Fold change is calculated after treating cells with TBHP to induce ROS formation. Fold change represents TBHP stimulation/basal line culture conditions. Data are presented as mean ± SD, *n* = 5 per group. Statistical analysis was performed using Student’s *t* test. ∗∗∗*p* < 0.001.

**Figure 5:**
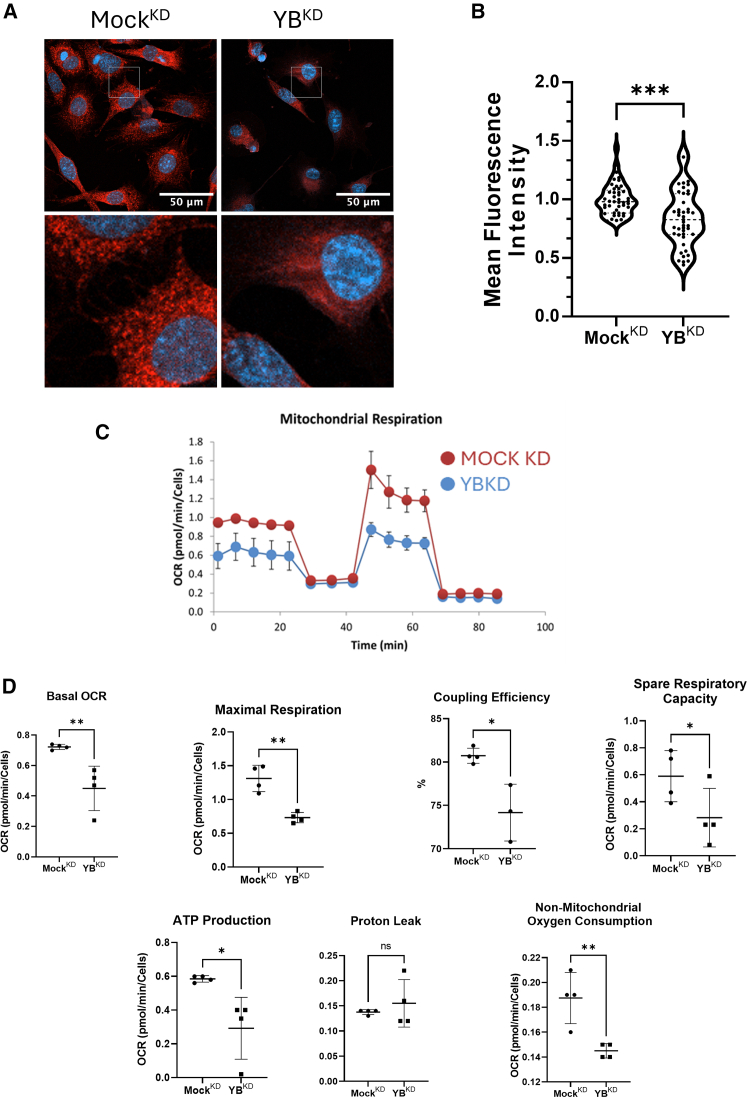
HuR KD induces mitochondrial dysfunction (A) Fluorescent microscopic images of Mitotracker CMXRos Red staining mitochondria. Scale bars: 50 μm. (B) Mean fluorescent intensity (MFI) of Mitotracker CMXRos Red staining mitochondria. Data are presented as mean ± SD, *n* = 4. Statistical analysis was performed using Mann-Whitney U test. ∗∗∗*p* < 0.001. (C) Seahorse XF Cell Mito-Stress showing oxygen consumption rate (OCR) in pmol/min. (D) Parameters of mitochondrial respiration. Data are presented as mean ± SD, *n* = 4. Statistical analysis for basal OCR, maximal respiration rate, coupling efficiency, and spare respiratory capacity was performed using Student’s *t* test. ATP production, proton leak, and non-mitochondrial oxygen consumption were analyzed using the Mann-Whitney U test. ∗*p* < 0.05 and ∗∗*p* < 0.01.

We knocked down HuR in MLO-Y4 cells (Y^KD^) using two independent short hairpin RNA (shRNA) constructs, both of which effectively reduced HuR protein levels (Figure 4A). Despite comparable KD efficiency, we observed differences between the two constructs. Construct A (YA^KD^) induced severe cytotoxicity, including a significant loss of cell viability (Figure 4B) and dramatic morphological changes characterized by a severely reduced cytoplasmic volume and near complete loss of dendrites (Figures 4C and 4D). Consistently, gene set enrichment analysis (GSEA) of YA^KD^ revealed enrichment in DNA damage and cell-cycle checkpoint pathways, alongside terms related to RNA metabolism and splicing, despite minimal evidence for differential splicing (Figures 5A and 5B). By contrast, construct B (YB^KD^) experienced reduced cell viability and morphological changes, though to a lesser extent than YA^KD^, and preserved cell dendrites (Figures 4C and 4D). The absence of detectable splicing changes in YA^KD^, combined with its pronounced cytotoxic phenotype and the lack of prior reports employing this construct, raised concerns about off-target toxicity. In contrast, construct B has been validated in multiple independent studies.^33^^,^^34^^,^^35^^,^^36^ To avoid confounding artifacts, we therefore selected YB^KD^ for further analysis; hereafter, the terms HuR KD and YB^KD^ are used interchangeably. RNA-seq analysis of YB^KD^ revealed 129 differentially expressed genes (DEGs) at |log2FC| ≥ 1 and adjusted *p* value <0.05 compared to Mock^KD^, including HuR downregulation (Figure 4E). The top 10 enriched pathways in pre-ranked GSEA showed downregulation of multiple terms. Among these, signaling by platelet-derived growth factor (PDGF) and collagen degradation are particularly relevant to skeletal biology, as PDGF signaling regulates osteoblast survival and matrix remodeling,^37^^,^^38^ while collagen turnover is central to bone matrix maintenance. In addition, IL-6 family signaling is involved in inflammation, osteoclast formation, and osteogenesis.^39^ Other downregulated pathways included growth and developmental programs (middle ear morphogenesis) and lipid metabolic processes (fatty acid and cholesterol biosynthesis). Upregulated pathways showed a clear trend toward enrichment in pathways related to quality control of mRNA processing, translation, and ribosomal terms. Pathways related to immunity (response to virus) and pathways related to cytoskeleton organization (straited muscle contraction) (Figure 4F and Table S3) were also enriched. Overall, the transcriptomic profile of HuR KD points to a complex role for HuR in controlling several aspects of cellular homeostasis, including skeletal-associated pathways involved in matrix remodeling and inflammatory signaling, as well as a role in regulating RNA processing and translation.

### HuR KD sensitizes cells to hyperglycemic and oxidative stress-induced cell death

Our initial analysis pointed to the HuR motif as a top enriched motif around spliced transcripts in HG conditions; therefore, we assessed the effect of HG on YB^KD^ cells. We cultured cells with NG or HG and M as an osmotic pressure control, and we used H_2_O_2_ as a positive control. HG induced cell death only in YB^KD^ cells but not in Mock^KD^ after 48 and 72 h, indicating that HuR KD sensitizes cells to HG injury. Hydrogen peroxide induced oxidative cell death in both cell lines, but with markedly greater lethality in YB^KD^ compared to Mock^KD^ (Figure 4G). HG is known to induce oxidative stress; therefore, we next examined whether HuR KD affected redox homeostasis and cellular susceptibility to oxidative stress. We performed a 2′,7′-dichlorofluorescin diacetate (DCFDA) assay, which measures reactive oxygen species (ROS) levels. The results indicated a decrease in ROS production under baseline culture conditions in YB^KD^; however, upon treating cells with *tert*-butyl hydroperoxide (TBHP), an ROS-generating compound, ROS accumulation doubled in YB^KD^ compared to Mock^KD^ (Figures 4H and 4I).

### HuR KD induces mitochondrial dysfunction

To quantify the mitochondrial density, we used Mitotracker CMXRos Red, a selective mitochondrial dye. The mitochondria localized around the nuclei and extended to dendrites, and we observed reduced mean fluorescence intensity in YB^KD^ cells compared to Mock^KD^ (Figures 5A and 5B). Next, we measured mitochondrial function using Seahorse XF Cell Mito-Stress test (Figure 5C). YB^KD^ cells exhibited reduced mitochondrial respiration. Basal oxygen consumption rate (OCR), maximal respiration, spare respiratory capacity, coupling efficiency, and ATP production were reduced. Proton leak remained unchanged, suggesting that the mitochondrial membrane is intact. The observed reduction in OCR also extended to non-mitochondrial respiration, suggesting that the decline in oxygen consumption is partly attributable to reduced activity of non-mitochondrial oxygen-consuming enzymatic reactions (Figure 5D). These data agree with the reduced basal ROS production observed in YB^KD^ cells and indicate that the increased ROS is not due to increased basal mitochondrial ROS production.

### HuR KD alters translational and metabolic stress adaptation splicing programs

**Figure 6:**
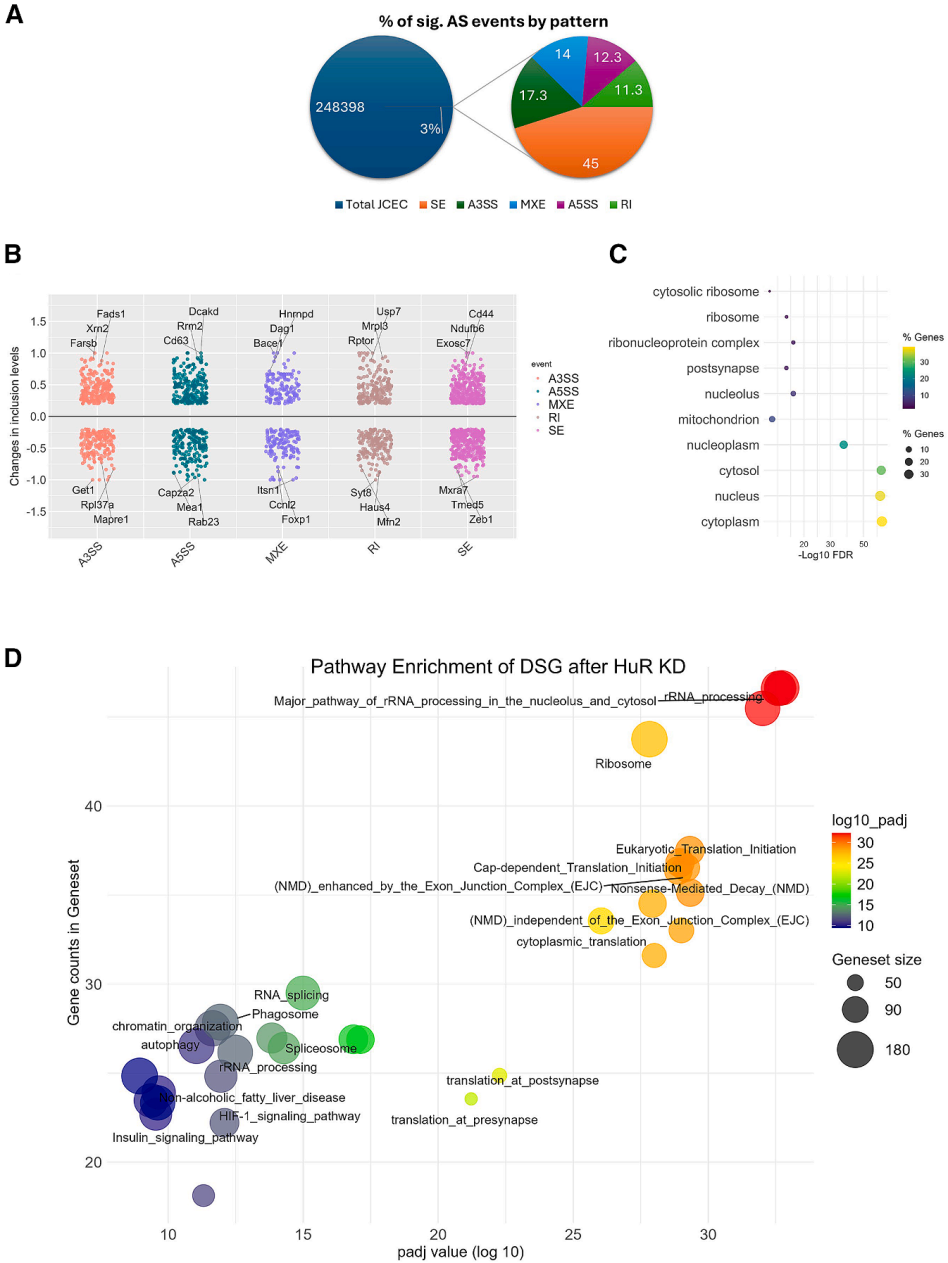
HuR KD induces AS changes in MLO-Y4 cells (A) The parent pie chart shows significant AS events (3%) of the total events (JCEC), and secondary pie chart showing the percentage of significant AS events of each type (SE, A5SS, A3SS, MXE, and RI) from total significant events. (B) Scatterplot showing DSGs around AS events in 5 patterns FDR <0.05 and 0.2 < ΔѰ < −0.2. (C) Gene Ontology Cellular Component (GOCC) enrichment analysis of DSGs performed using the DAVID Functional Annotation Tool. (D) ORA of DSGs after YB^KD^. Also, see Table S8.

We further compared alternative mRNA splicing patterns between YB^KD^ and Mock^KD^. We summarized the output of the AS analysis from junction and exon body counts (JCECs). The total number of JCEC events was 24,000 events, with 3% showing significance between YB^KD^ and Mock^KD^. Among the AS patterns detected, SE represented the most frequently regulated events, while RI events were the least regulated (Figure 6A). This observation aligns with the earlier HuR motif analysis in primary osteocytes (Figures S2 and S3), where RI events showed the least enrichment in HuR motifs around splicing events. DSGs (Figure 6B and Table S4) were used to perform Gene Ontology (GO) and overrepresentation analysis (ORA) following HuR KD. The top 10 scoring cellular components included ribonucleoprotein complexes (RNPs), ribosomes, and mitochondria (Figure 6C). ORA analysis results revealed two major clusters of enriched pathways: first, translation-related terms, including rRNA processing, RNA splicing, mRNA surveillance (nonsense-mediated decay), ribosomal subunits, and translational regulation consistent with HuR’s role as an RBP; second, metabolic or stress-response terms, including autophagy, HIF-1α signaling, and insulin signaling pathways (Figure 6D and Table S4).

### HuR KD remodels translational signaling pathways while preserving global protein output

**Figure 7:**
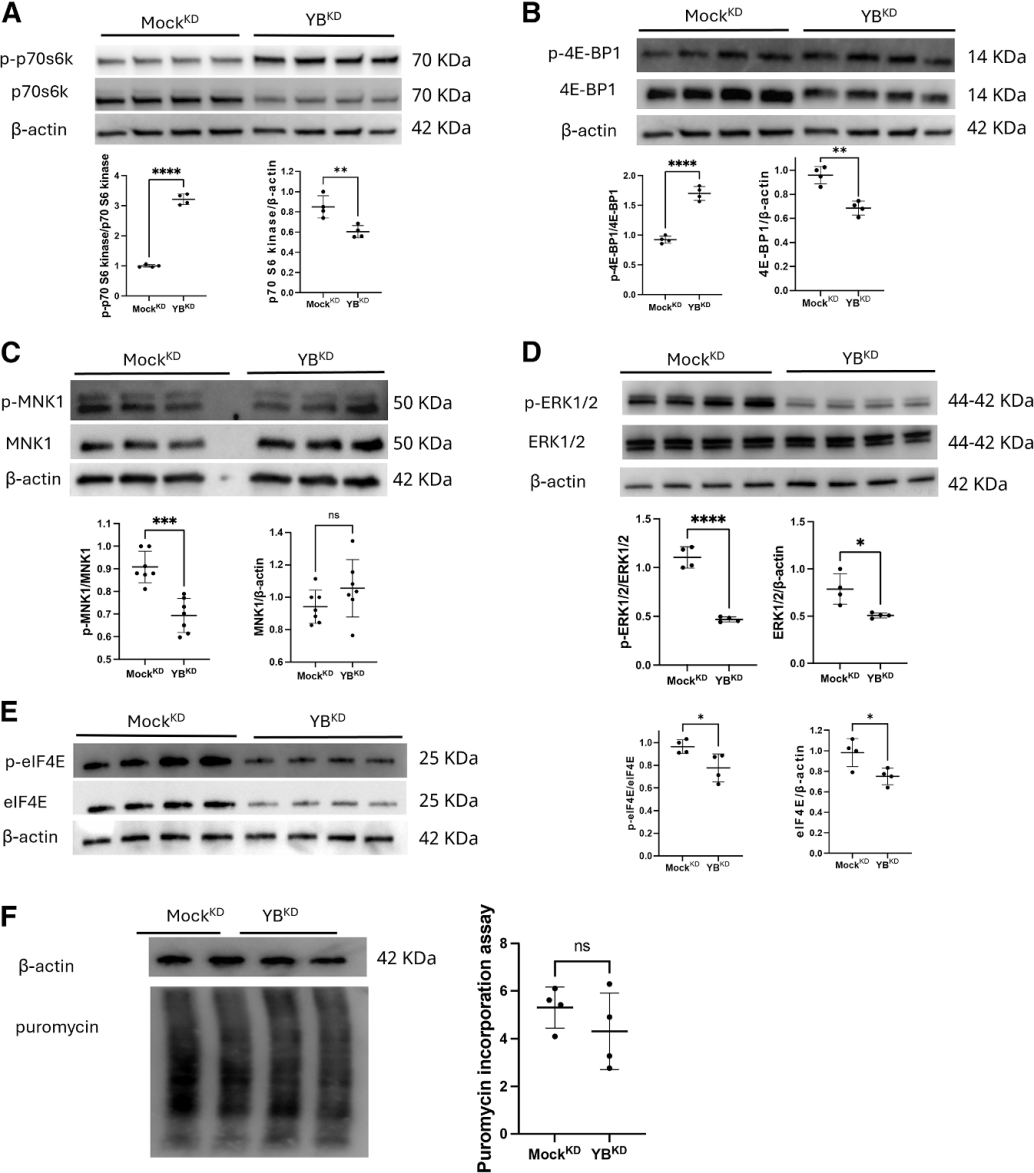
HuR KD remodels translation signaling pathways and preserves global protein synthesis rate (A) Western blot of p-p70S6K and p70S6K protein levels. *n* = 4. (B) Western blot of p-4E-BP1 and 4E-BP1 protein levels. *n* = 4. (C) Western blot of p-MNK1 and MNK1 protein levels. *n* = 7. (D) Western blot of p-ERK1/2 and ERK1/2 protein levels. *n* = 4. (E) Western blot of p-eIF4E and eIF4E protein levels. *n* = 4. (F) Western blot of puromycin incorporation assay. *n* = 4. The same β-actin blot is shown in (A) and (B) because p-P70S6K/P70S6K and p-4EBP1/4 EBP1 were detected from the same membrane after sequential stripping and reprobing, using the same sample lanes. β-actin served as the shared loading control for this membrane. Data are presented as mean ± SD of 2 or more independent blots. All the statistical analysis was performed using Student’s *t* test. ∗*p* < 0.05, ∗∗*p* < 0.01, ∗∗∗*p* < 0.001, and ∗∗∗∗*p* < 0.0001.

Our DSG analysis revealed significant enrichment in pathways related to translational control; therefore, we investigated several key translational signals. We assessed the phosphorylated status of the mechanistic target of rapamycin (mTOR), a central mediator of mRNA translation and protein synthesis. mTOR phosphorylates two key immediate targets, the p70S6 kinase (p70S6K) and eukaryotic initiation factor 4E-binding protein 1 (4E-BP1).^40^ Total levels of p70S6K and 4E-BP1 were reduced in HuR KD cells. However, their phosphorylated forms, p-p70S6K and p-4E-BP1, increased, resulting in increased phosphorylated-to-total protein ratios (Figures 7A and 7B). We next examined mitogen-activated protein kinase-interacting serine/threonine kinase 1 (MAPK-MNK1) axis, which predominantly regulates cap-dependent translation initiation, the predominant form of translation in eukaryotic cells. Phosphorylated MNK1 decreased, whereas total MNK1 levels remained unchanged (Figure 7C). Consistently, upstream of MNK1, the extracellular signal-regulated kinases1/2 (ERK1/2) showed reduced levels of both phosphorylated and total protein (Figure 7D). Furthermore, these pathways converge on the eukaryotic initiation factor 4E (eIF4E), a direct substrate of MNK1 and 4E-BP1. In HuR KD cells, both total and phosphorylated levels were reduced, leading to a lower p-eIF4E/eIF4E ratio (Figure 7E). To determine whether these molecular changes affected overall protein translation, we performed a puromycin incorporation assay to measure the nascent protein synthesis rate. Despite the pronounced alterations in these translational regulators, no significant differences in global protein synthesis rates were detected, suggesting maintenance of translational output through the compensatory activation of mTOR downstream targets (Figure 7F).

### HuR KD disrupts TXNIP RNA-protein coupling

**Figure 8:**
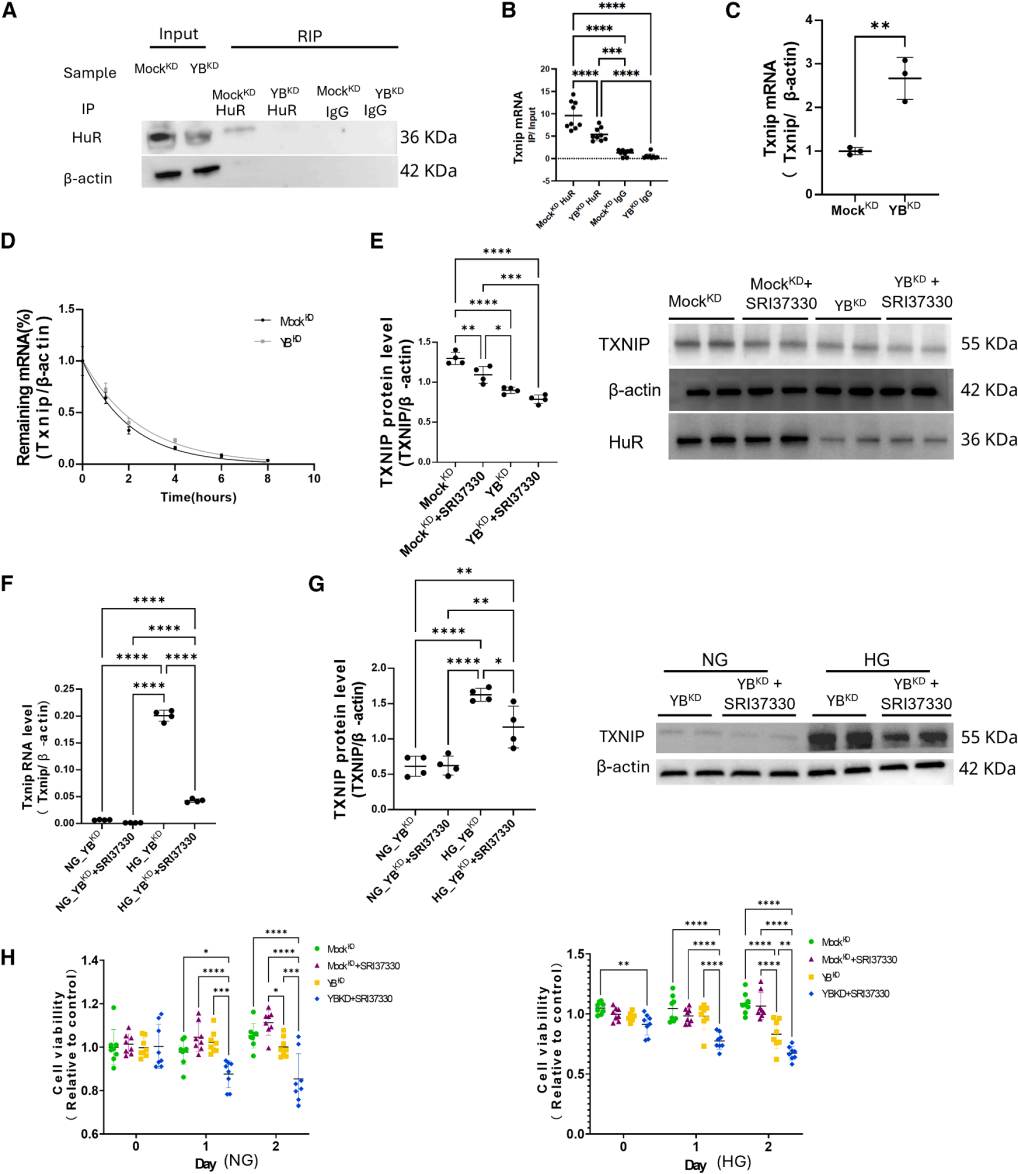
HuR KD induces TXNIP RNA-protein uncoupling (A) Immunoprecipitation plots confirming the presence of HuR protein in the immunoprecipitated samples using an anti-HuR antibody compared to IgG control. *n* = 3. (B) RIP qPCR for *Txnip* in MLO-Y4 cells using anti-HuR or non-specific IgG (control). Data are presented as relative quantity normalized to input presented as mean ± SD, *n* = 9. Statistical analysis was performed using one-way ANOVA. ∗∗∗*p* < 0.001, and ∗∗∗∗*p* < 0.0001. (C) *Txnip* mRNA level in MLO-Y4 cells after HuR KD relative to β-actin. Data are presented as mean ± SD, *n* = 3. Statistical analysis was performed using Student’s *t* test. ∗∗*p* < 0.01. (D) *Txnip* mRNA stability assay following actinomycin D relative to β-actin. Decay curves were fitted using a one-phase exponential model; half-life increased from 1.4 h in Mock^KD^ to 1.7 h in YB^KD^. Data points represent mean ± SEM (*n* = 6), *p* < 0.01 for decay rate constant (K) comparison. (E) TXNIP protein levels in Mock^KD^ and YB^KD^ in NG conditions. Data are presented as mean ± SD, *n* = 4. Statistical analysis was performed using one-way ANOVA followed by Tukey’s post hoc test. ∗*p* < 0.05, ∗∗*p* < 0.01, ∗∗∗*p* < 0.001, and ∗∗∗∗*p* < 0.0001. (F) *Txnip* mRNA levels in YB^KD^ in NG and HG conditions. Data are presented as mean ± SD, *n* = 4. Statistical analysis was performed using one-way ANOVA followed by Tukey’s post hoc test. ∗∗∗∗*p* < 0.0001. (G) TXNIP protein levels in YB^KD^ in NG and HG conditions. Data are presented as mean ± SD, *n* = 4. Statistical analysis was performed using one-way ANOVA followed by Tukey’s post hoc test. ∗*p* < 0.05, ∗∗*p* < 0.01, and ∗∗∗∗*p* < 0.0001. (H) Cell viability in Mock^KD^ and YB^KD^ cells in 5.5 mM/NG (left) vs. 25 mM/HG (right) with and without SRI37330 treatment. Data are presented as mean ± SD, *n* = 8. Statistical analysis was performed using one-way ANOVA followed by Tukey’s post hoc test. ∗*p* < 0.05, ∗∗*p* < 0.01, ∗∗∗*p* < 0.001, and ∗∗∗∗*p* < 0.0001.

To determine whether HuR coordinates transcript fate with protein output, we focused on *Txnip*, a glucose-responsive redox regulator and HuR target.^41^ RNA immunoprecipitation (Figure 8A) confirmed association, as HuR antibody efficiently enriched *Txnip* mRNA compared to IgG control (Figure 8B). HuR depletion increased *Txnip* mRNA abundance (Figure 8C). Actinomycin D chase experiments revealed that this increase was partly attributable to enhanced transcript stability, with a modest but significant ∼20% increase of mRNA half-life (Δt½ = 1.42 → 1.72 h) (Figure 8D). Interestingly, this transcriptional and post-transcriptional gain was not reflected at the protein level. TXNIP protein abundance was reduced in HuR KD cells (Figure 8E), revealing an inverse RNA-protein relationship. These data indicate that HuR loss disrupts the coupling between transcript abundance and translational output. To test whether transcript availability limited the protein production, we pharmacologically suppressed *Txnip* expression using SRI37330, a transcriptional inhibitor. In control cells, reducing transcript levels led to a decrease in protein abundance, consistent with transcription-dependent protein output. In contrast, in HuR KD cells, SRI37330 did not significantly alter protein levels, indicating that protein output was no longer limited by transcript abundance. These findings suggest that HuR depletion rewires TXNIP expression at the post-transcriptional or translational levels. Because HG is a potent inducer of TXNIP, we next examined this axis under hyperglycemic conditions. HG increased *Txnip* mRNA and its protein in HuR KD cells as expected, and SRI37330 effectively reduced both (Figures 8F and 8G). Notably, SRI37330 did not impact Mock^KD^ cells but decreased viability selectively in HuR KD cells under both NG and HG conditions, revealing increased dependence on TXNIP regulation in the absence of HuR (Figure 8H). Together, these findings demonstrate that HuR associates with *Txnip* mRNA and that HuR coordinates RNA-protein coupling. Its depletion induces a state of transcription-translation uncoupling, exposing translational rewiring as a key layer of post-transcriptional regulation.

## Discussion

RNA fate and function are largely determined by its interaction with RBPs. HuR is a ubiquitously expressed RBP that interacts with adenine- and uridine-rich elements located within the 3′ untranslated regions (3′ UTRs) of target mRNAs, regulating their stability and AS.^9^^,^^42^^,^^43^ HuR is a key modulator of metabolic disease, and its role appears to vary across cells, tissues, and disease models, where it can be protective or maladaptive.^22^^,^^23^^,^^44^^,^^45^^,^^46^ These context-specific discrepancies also manifest in the skeletal system, underscoring the context- and cell-type-dependent nature of HuR function.^25^^,^^26^

Our transcriptome-wide analysis in osteocytes exposed to HG revealed minimal differential gene expression, whereas AS changes were prominent and regulated a wide array of cellular programs. These findings prompted us to investigate post-transcriptional regulation under HG. We analyzed the sequences surrounding differential splicing events in the DSGs to identify enriched RBP motifs. This analysis revealed significant enrichment of binding motifs recognized by several RBPs including HuR, ZC3H14, and HNRNPL, as recurrently enriched. HuR emerged as a prominent candidate, appearing in multiple positions across various splicing events. Therefore, we examined HuR expression in osteocytic MLO-Y4 cells cultured in HG and in the bones of HFD mice. HuR mRNA and protein levels remained unchanged.

HuR KD in MLO-Y4 cells exhibited profound differential gene expression changes, including reduced expression of genes involved in extracellular matrix (ECM) remodeling, consistent with its reported role in maintaining several ECM proteins including collagens and matrix metalloproteinases critical for bone matrix remodeling.^47^ DEGs also showed downregulation in cytoskeletal organization, reflecting cellular morphological changes, which can impact mechanosensing and transduction.^48^^,^^49^ Consistent with that, phalloidin staining showed rounding and loss of dendrites consistent with GSEA. Because osteocyte mechanotransduction depends on dendritic architecture, cytoskeletal organization, and extracellular matrix connectivity, these structural alterations suggest that HuR deficiency may indirectly influence mechanical signal transmission in osteocytes, although direct mechanical loading experiments were not performed in the present study.

Additionally, enriched pathways were clearly biased toward mRNA quality control and mRNA translation. HuR KD increased the sensitivity of osteocytic cells to HG levels and oxidative stress and displayed elevated ROS levels following exposure to oxidative agents, supporting a protective role for HuR in maintaining redox balance.

Given that mitochondria are the primary source of cellular ROS, we assessed mitochondrial mass and function. HuR KD reduced mitochondrial content and respiration and decreased OCR, maximal respiration, coupling efficiency, and ATP production, while proton leak remained unchanged, suggesting that mitochondrial membrane integrity was preserved. The decrease in ROS under baseline conditions may be explained by reduced mitochondrial activity and total oxygen consumption by cells, reducing ROS-generating capacity. However, the heightened vulnerability to exogenous oxidative stress under HG- and ROS-inducing conditions highlights a failure of adaptive antioxidant response in HuR KD cells. Collectively, these findings underscore HuR’s role in maintaining cell viability under HG conditions and maintaining redox homeostasis and mitochondrial function.

DSG enrichment analysis correlated well with transcriptional changes (DEGs) and showed extensive enrichment in ribosomal and translational terms. Furthermore, AS analysis revealed significant enrichment in stress-related pathways, including autophagy and HIF-1α signaling, as well as key metabolic pathways such as insulin signaling and non-alcoholic fatty liver disease. These pathways were not detected at the gene expression level, which emphasizes the added resolution provided by splicing analyses.

Given the enrichment of gene expression and AS events in pathways related to mRNA translation and protein synthesis, we examined whether these changes were functionally reflected in translational signaling. Eukaryotic translation is initiated primarily through cap-dependent mechanisms, a rate-limiting process governed by eIF4E availability.^50^ eIF4E activity is regulated by the inhibitory 4E-BP1, which releases eIF4E upon its phosphorylation by mTORC1 kinase,^40^ and phosphorylation of eIF4E by MNKs. While MNK-mediated phosphorylation is dispensable for global protein synthesis, mTORC1 inhibition leads to near complete inhibition of mRNA translation.^51^^,^^52^

Our data revealed reduced total levels of p70S6K and 4E-BP1, yet their phosphorylated forms were elevated, resulting in increased phospho-to-total ratios, which suggest relative activation of mTORC1 kinase. In contrast, the MNK1-eIF4E axis was suppressed: phosphorylated MNK1 and its upstream kinase ERK1/2 were reduced, and both total and phosphorylated eIF4E levels were diminished. Despite these alterations, global protein synthesis rates remained preserved. These data indicate preserved mTORC1-driven translation with attenuation of the MNK-eIF4E axis, consistent with translational remodeling rather than global suppression.

To determine whether this translational reprogramming functionally impacts specific HuR targets, we examined TXNIP.^43^ We found that HuR coordinated TXNIP transcript availability with protein output. HuR KD increased *Txnip* mRNA abundance and stability, yet protein levels were reduced, indicating disruption of RNA-protein coupling. Pharmacologic suppression of *Txnip* transcription further supported this model; in control cells, protein levels decreased with decreasing transcript availability, whereas in HuR KD cells, reducing mRNA levels did not proportionally alter protein abundance, indicating that TXNIP levels are no longer limited by how much mRNA is made, but by how efficiently it is translated.

To establish whether this dysregulation has functional consequences under metabolic stress, HuR KD cells were exposed to HG in the presence or absence of a *Txnip* pharmacologic inhibitor. HG increased both *Txnip* mRNA and protein levels. Pharmacological inhibition reduced transcript abundance and was accompanied by a corresponding decrease in protein levels. This contrasts with basal conditions in HuR KD cells, where *Txnip* mRNA and its protein levels were uncoupled. Under HG, however, TXNIP protein levels increased in parallel with mRNA, indicating that the relationship between transcript abundance and protein output differs depending on metabolic state. Functionally, TXNIP inhibition reduced cell viability in HuR KD cells but not in control cells, suggesting that HuR-deficient cells are more sensitive to perturbations in TXNIP.

In summary, our findings define HuR as an important regulator of osteocyte adaptation to metabolic stress. HuR loss reshapes gene expression and AS, disrupts mitochondrial function and redox balance, and remodels translational signaling without altering global protein synthesis. The uncoupling of *Txnip* mRNA and its protein under basal conditions highlights altered coordination between transcript abundance and protein output in HuR KD cells. Together, these data position HuR as a key integrator of post-transcriptional regulation in osteocytes and emphasize the importance of translational control in skeletal responses to hyperglycemic stress.

### Limitations of the study

This study has several limitations that should be considered when interpreting the findings. First, although we employed both primary osteocytes and MLO-Y4 cells, our *in vitro* models do not fully recapitulate the complex *in vivo* environment of bone. All *in vitro* experiments were conducted on tissue culture plastic, and emerging evidence demonstrates that osteocyte gene expression and cell behavior are influenced by substrate dimensionality and stiffness when cells are cultured in 3D, which should be considered in future analysis systems.^53^ Moreover, HFD-induced hyperglycemia *in vivo* and HG culture *in vitro* represent complementary contexts; the HFD model induces chronic hyperglycemia in the context of systemic alterations, including fluctuating glucose and insulin levels and increased inflammatory load. Moreover, exposing cells to stably elevated glucose in culture does not recapitulate the dynamic fluctuations observed *in vivo*; however, this is a valuable approach for isolating the direct effects of glucose on cellular responses, independent of confounding systemic factors. Future work using conditional HuR knockout mice will be important to validate these mechanisms in the skeleton.

Although both shRNA constructs achieved comparable KD levels of HuR, their cellular effects were markedly different. Construct A induced severe cytotoxicity, dendrite loss, and activation of DNA damage and checkpoint pathways, consistent with a generalized stress response (Figure S5). In contrast, construct B preserved dendritic morphology and produced a transcriptomic profile consistent with established HuR functions. Importantly, construct B has been employed extensively in the literature to study HuR biology across different systems, including colorectal cancer,^33^ acute myeloid leukemia,^34^^,^^35^ and osteosarcoma.^36^ By comparison, we were unable to identify any studies using construct A. On this basis, we selected construct B for downstream analysis.

shRNA-mediated KD remains one of the most widely used and validated strategies for probing gene function, offering several practical advantages; nevertheless, caveats are inherent to shRNA-based approaches. Off-target silencing can occur and may influence gene expression independently of the intended target. Reliance on a single construct raises the possibility that residual off-target effects remain, even when the observed phenotypes are consistent with published literature. For these reasons, our use of two independent shRNAs to initially interrogate HuR function was critical for identifying potential off-target effects. While we recognize these limitations, the extensive validation of our results and widespread application of construct B shRNA KD lend confidence to the specificity of our findings.

## Resource availability

### Lead contact

Requests for further information and resources should be directed to and will be fulfilled by the lead contact, Aseel Marahleh (aseel.mahmoud.suleiman.marahleh.e6@tohoku.ac.jp).

### Materials availability

This study did not generate new unique reagents. All resources and materials reported in this paper will be shared by the lead contact upon request.

### Data and code availability

• Raw sequencing data are publicly available in the NCBI Sequence Read Archive (SRA: SRP584780; BioProject: PRJNA1262104).
• This paper does not report original code.
• Any additional information required to reanalyze the data reported in this paper is available from the lead contact upon request.

## Acknowledgments

This work is supported by The FRIS Creative Interdisciplinary Collaboration Program and a 10.13039/501100001691Japan Society for the Promotion of Science, 10.13039/501100001691JSPS Kakenhi grant #25K20447 to A.M. and JST 10.13039/501100025019SPRING to Z.F. (grant no. JPMJSP2114).

## Author contributions

Conceptualization, A. Marahleh.; methodology, A. Marahleh., Z. Fan., and S. Rashad.; investigation, Z. Fan. and A. Mousa.; writing – original draft, A. Marahleh.; writing – review and editing, A. Marahleh., Z. Fan., and S. Rashad.; funding acquisition, A. Marahleh. and Z. Fan.; resources, H. Kitaura, J. Ren., F. Ohori., K. Niizuma., and H. Kanetaka; supervision, A. Marahleh. and H. Kitaura.

## Declaration of interests

The authors report no competing interests.

## Declaration of generative AI and AI-assisted technologies in the writing process

No generative AI or AI-assisted technologies were used during the preparation of this work.

## STAR★Methods

### Key resources table

**Table:** 

REAGENT or RESOURCE	SOURCE	IDENTIFIER
**Antibodies**		

Anti- eIF4E Antibody	Cell Signaling Technology	Cat# 9742; RRID: AB_823488
Phospho-eIF4E (Ser209) Antibody	Cell Signaling Technology	Cat# 9741; RRID: AB_331677
p70 S6 Kinase Antibody	Cell Signaling Technology	Cat# 9202; RRID: AB_331676
Phospho-p70 S6 Kinase (Thr389) Antibody	Cell Signaling Technology	Cat# 9205; RRID: AB_330944
Mnk1 (C4C1) Rabbit mAb	Cell Signaling Technology	Cat# 2195; RRID: AB_2235175
Phospho-Mnk1 (Thr197/202) Antibody	Cell Signaling Technology	Cat# 2111; RRID: AB_2266303
p44/42 MAPK (Erk1/2) Antibody	Cell Signaling Technology	Cat# 9102; RRID: AB_330744
Phospho-p44/42 MAPK (Erk1/2) (Thr202/Tyr204) Antibody	Cell Signaling Technology	Cat# 9101; RRID: AB_331646
4E-BP1	Cell Signaling Technology	Cat# 9452; RRID: AB_331692
Phospho-4E-BP1 (Ser65) Antibody	Cell Signaling Technology	Cat# 9451; RRID: AB_330947
TXNIP (D5F3E) Antibody	Cell Signaling Technology	Cat# 14715; RRID: AB_2714178
Anti-β-Actin (ACTB) Antibody	Sigma-Aldrich	Cat# A2228; RRID: AB_476697
Anti-HuR/ELAVL1 antibody [EPR17397]	Abcam	Cat# ab200342; RRID: AB_2784506
Anti-Puromycin antibody [EPR27218-173]	Abcam	Cat# ab315887; RRID: AB_3716527 (pending approval in Antibody Registry)
Rabbit IgG	Sigma-Aldrich	Cat# PP64; RRID: AB_97852
Anti-rabbit IgG, HRP-linked Antibody	Cell Signaling Technology	Cat# 7074; RRID: AB_2099233
Sheep Anti-Mouse IgG - Horseradish Peroxidase	Cytiva	Cat# NA931; RRID: AB_772210

**Bacterial and virus strains**		

ECOS™ Competent E. coli DH5α	Nippon Gene	Code# 310-06236

**Chemicals, peptides, and recombinant proteins**		

Actinomycin D	Sigma Aldrich	Cat# A9415-5 MG
TRIzol Reagent	Invitrogen	Cat# 15596018
β-Mercaptoethanol	Bio-Rad	Cat# 1610710
Insulin	Sigma-Aldrich	Cat# I6634
Alexa Fluor 568 Phalloidin	Invitrogen	Cat# A12380
DAPI (4′,6-diamidino-2-phenylindole, dihydrochloride)	Thermo Fisher Scientific	Cat# 62248
SRI-37330 hydrochloride	Selleck Chemicals	Cat# E1272

**Critical commercial assays**		

Qiagen RNeasy kit	QIAGEN	Cat# 74104
SuperScript® IV First-Strand Synthesis System	Invitrogen	Cat# 18091200
Cell Counting Kit-8	Dojindo Laboratories	Cat# 343-07623
EZ-Magna RIP™ RNA-Binding Protein Immunoprecipitation Kit	Sigma-Aldrich	Cat# 17-701
DCFDA/H2DCFDA-Cellular ROS Assay Kit	Abcam	Cat# ab113851
Mito Stress Test Kit	Agilent	Cat# 103015-100
Seahorse XFe96/XF Pro FluxPak	Agilent	Cat# 103792-100
Seahorse XF DMEM medium	Agilent	Cat# 103575-100
MitoTracker™ Red CMXRos FM	Invitrogen	M7512

**Deposited data**		

The sequencing data have been deposited in the NCBI Sequence Read Archive (SRA) under accession number SRP584780.	This paper	Accession number SRA: SRP584780; BioProject: PRJNA1262104

**Experimental models: Cell lines**		

MLO-Y4 Transformed cell line	AddexBio Technologies	AddexBio Cat# P0004012

**Experimental models: Organisms/strains**		

Mouse: C57BL/6-Tg(Dmp1-Topaz)1Ikal/J	JAX Mice and Services	RRID: IMSR_JAX:017467
Mouse C57BL/6	Clea Japan	N/A

**Oligonucleotides**		

shRNA HuR targeting sequence Construct A (YAKD) CATTGGGAGAACGAATTTAAT	VectorBuilder’s shRNA Target Design tool	N/A
shRNA HuR targeting sequence Construct B (YBKD) TTGTTAGTGTACAACTCATTT	VectorBuilder’s shRNA Target Design tool	N/A
Primers for qPCR experiments can be found in Table S5	Sigma Aldrich	N/A

**Recombinant DNA**		

Plasmid: pLKO.1 puro	Addgene	RRID: Addgene_8453
Plasmid: psPAX2	Addgene	RRID: Addgene_12260
Plasmid: pMD2.G	Addgene	RRID: Addgene_12259

**Software and algorithms**		

rMATs	Shen et al.^31^	https://rnaseq-mats.sourceforge.io/
rMAPs2	Hwang et al.^32^	http://rmaps.cecsresearch.org/
Trimmomatic	Bolger et al.^54^	http://www.usadellab.org/cms/index.php?page=trimmomatic
HISAT2	Kim et al.^55^	https://daehwankimlab.github.io/hisat2/
FeatureCounts	Liao et al.^56^	https://subread.sourceforge.net/featureCounts.html
Limma-Voom	Law et al.^57^	https://insightsengineering.github.io/hermes/latest-tag/reference/h_diff_expr_voom.html
eVITTA	Cheng et al.^58^	https://tau.cmmt.ubc.ca/eVITTA/
ImageJ	Schneider et al.^59^	https://imagej.net/ij/
GraphPad Prism 9	N/A	https://www.graphpad.com/features
Rstudio 4.4.0	Posit team (2025). RStudio: Integrated Development Environment for R. Posit Software, PBC, Boston, MA.	http://www.posit.co/.
Evolution-Capt Edge	Vilber Lourmat, France	N/A

### Experimental model and study participant details

#### Animals and animal care

Wild-type male C57BL/6J mice (*Mus musculus*) and C57BL/6-Tg (Dmp1-Topaz)1Ikal/J mice (*Mus musculus*) were used in this study. 12-week-old wild-type male C57BL/6J mice housed in pairs were acclimated for 4 weeks in specific pathogen-free (SPF) conditions under a 12-h light/dark cycle with *ad libitum* feeding (Research Diet, D12450H, Japan) and at 16 weeks the mice were randomly allocated to normal (Research Diet, D12450H, Japan) or high fat (Research Diet, D12451, Japan) dietary intervention for 16 weeks.

Neonatal 7-9-day-old C57BL/6-Tg (Dmp1-Topaz)1Ikal/J mice expressing the topaz variant of green fluorescent protein (GFP) under the direction of the mouse dentin matrix protein 1 (Dmp1) promoter were housed in specific pathogen-free (SPF) conditions under a 12-h light/dark cycle with parent access to *ad libitum* feeding of regular chow diet.

#### Sex as a biological variable

Female mice were not included in the diet intervention study. The use of adult male mice minimizes potential confounding effects of sex hormones, which are known to influence bone metabolism in adulthood. Sex determination is not feasible in neonatal mice as mice pups have not yet developed distinguishable sexual characteristics; therefore, the influence of sex on the reported outcomes is not applicable.

#### Study approval

All procedures involving animals were performed in accordance with the ARRIVE guidelines and were approved by the Regulations for Animal Experiments and Related Activities at Tohoku University with institutional approval document ID (2021DnLMO-007-04).

#### Primary cells

Primary osteocytes cells were isolated from the calvaria of 7-9-day-old C57BL/6-Tg (Dmp1-Topaz)1Ikal/J mice (*Mus musculus*) using enzymatic digestion and fluorescent activated cell sorting (FACS) as described below. After isolation, cells were cultured at standard conditions (37 ֯Ϲ, 5% CO_2_) overnight in alpha-minimum essential medium (ꭤ-MEM; Wako Pure Chemical Industries, Osaka, Japan) containing 10% fetal bovine serum (FBS; Biowest, Nuaillé, France), 100IU/mL penicillin G, 100 μg/mL streptomycin (1% P/S) in 10 cm plastic culture dishes.

#### Cell lines

The murine osteocyte-like cell line MLO-Y4 cell line was obtained from AddexBio (P0004012) and certified PCR-tested Mycoplasma free. MLO-Y4 cells were maintained at 37°Ϲ, 5% CO_2_ in 10% FBS, 1% P/S and kept at 70–80% confluency and passaged as needed in plastic culture dishes.

### Method details

#### Primary cells and cell lines

Primary osteocytes were isolated from littermate neonatal calvariae of C57BL/6-Tg (*Dmp1-Topaz*)1Ikal/J (7–9 days) at which age the mice have not yet developed distinguishable sexual characteristics, therefore, sex of the mice could not be determined. The parent mice were kept on a regular chow diet. Using serial enzymatic digestion with collagenase and EDTA followed by FACS to isolate osteocytes as described previously.^60^ After isolation, cells were cultured at standard conditions (37°Ϲ, 5% CO_2_) overnight in ꭤ-MEM (Wako Pure Chemical Industries, Osaka, Japan) containing 10% FBS (Biowest, Nuaillé, France) and 1% P/S, containing 5.5 mM glucose (standard medium) in 10 cm plastic culture dishes. After that, the media was replaced with fresh 5.5 mM glucose (normal glucose, NG), 25 mM glucose (Sigma-Aldrich, St. Louis, MO, USA) (high glucose, HG) containing media or 5.5 mM glucose supplemented with 19.5 mM mannitol (osmotic control, M) (Sigma-Aldrich) for 3 days. MLO-Y4 cells (AddexBio) were maintained at 37°Ϲ, 5% CO_2_ in standard medium and kept at 70–80% confluency and passaged as needed. Standard medium was changed to NG, HG or M media for subsequent experiments.

#### Mice and feeding experiments

12-week-old male C57BL/6J mice were purchased from CLEA Japan (Tokyo, Japan). Mice were acclimated for 4 weeks in specific pathogen-free (SPF) conditions under a 12-h light/dark cycle with *ad libitum* feeding (Research Diet, D12450H, Japan) and at 16 weeks the mice were randomly allocated to a 10% Kcal from fat termed normal diet (ND) (Research Diet, D12450H, Japan) or 45% Kcal from fat termed high fat diet (HFD) (Research Diet, D12451, Japan) for 16 weeks to induce chronic hyperglycemia. Mice were housed in pairs. The mice were tested for fasting blood glucose and glucose tolerance at 4 and 8 weeks of diet and one day before collecting the bones at 16 weeks of diet with 4–5 mice per group. After sacrifice, the long bones (femur, tibia, humerus) were excised. The muscles and soft tissues were carefully removed, and the bones were scraped using a scalpel. The epiphyses were cut and the bone marrow flushed in phosphate-buffered saline (PBS) ×1 at 9000 xg, 4 ֯Ϲ three times. The bones were immediately frozen in liquid nitrogen and stored at −80 ֯Ϲ for RNA and protein extraction. All procedures involving animals were performed in accordance with the ARRIVE guidelines and were approved by the Regulations for Animal Experiments and Related Activities at Tohoku University (2021DnLMO-007-04).

#### Blood glucose measurement and metabolic tolerance tests

Fasting blood glucose tolerance and insulin tolerance tests were assessed after a 6-h fast (8:00 AM–2:00 PM) using a rodent specific glucometer with compatible LG sensors (LabGluco Funakoshi., Tokyo, Japan). Approximately, 5 μL or less blood was drawn from a tail tip clip at 0, 15, 30, 60, and 90 min for insulin tolerance tests and 120 min for glucose tolerance tests. Injection volumes were calculated as follows: GTT: glucose solution volume (Sigma-Aldrich) = 7.5 × body weight in grams from 20% glucose in saline solution. ITT: insulin solution volume (Novolin ® R) = 3 × body weight from 0.25 UI/mL insulin in saline solution Weights were measured immediately before each test and injections were carried out intraperitonially.

#### Short hairpin RNA KD

Two short hairpin RNA (shRNA) sequences targeting mouse *Elavl1* mRNA were designed using VectorBuilder’s shRNA Target Design tool. An empty backbone sequence was used as a non-targeting control. The shRNA oligonucleotides were ordered and synthesized by FASMAC Co., Ltd. (Kanagawa, Japan). Oligos were annealed and ligated and cloned into pLKO.1 puro vector (Addgene, Plasmid# 8453). Plasmids were then extracted using Nucleospin plasmid DNA purification kit (Macherey-Nagel, Düren, Germany) followed by validating the insertions using colony-PCR confirming sequences by Sanger Sequencing (Eurofins Genomics, Tokyo, Japan). HEK293T cells were co-transfected with shRNA plasmids, lentiviral packaging plasmid psPAX2 (Addgene, Plasmid#12260) and VSV-G envelope expressing plasmid pMD2.G (Addgene, Plasmid#12259) using Lipofectamine 3000 (Thermo Fisher Scientific, Waltham, MA, USA). Viral particles were collected after 48 and 72 h, centrifuged at 1500 xg for 5 min and supernatant was filtered through a 0.45 μm PVDF filter. Viral particles were stocked at 4 ֯Ϲ for immediate use or −80 ֯Ϲ for long term storage. MLO-Y4 cells were grown to 80% confluency in standard medium, then media was changed to transduction medium containing standard medium and lentiviral particles containing supernatant at 4:1 (by volume) and 8 μg/mL polybrene (Sigma-Aldrich, St. Louis, MO, USA) for 24 h. Transduction medium was then replaced with standard medium and incubated for 6 h then puromycin was added at (2 μg/mL) and transfected cells were selected for 7 days before passaging. Construct sequences of control Mock KD (Mock^KD^), construct A KD (YA^KD^) and construct B KD (YB^KD^) in Table S5. A and B denote 2 separate constructs and (Y) denotes that MLO-Y4 cells were used for KD.

#### RNA sequencing

Total RNA was extracted using Qiagen RNeasy kit (QIAGEN, Hilden, Germany) with a DNase digestion step (QIAGEN) as per manufacturer’s instructions. The samples were processed by Azenta, Japan as follows: samples were screened and quantified on an Agilent 4200 Tapestation with RNA ScreenTape. The resultant RIN (RNA integrity number) was 9.9 or 10 for all samples. mRNA was enriched using NEBNext Poly(A) mRNA Magnetic Isolation Module (NEB) and RNA library preparation was done NEBNext Ultra II Directional RNA Library Prep Kit (Illumina E7760). Samples were pooled on an Illumina Novaseq to obtain strand-specific 150 bp paired-end reads and 50M depth ensuring exon–exon junction coverage and accurate splice variant detection. FASTQ files were then used for bioinformatics analysis. All sequencing experiments were done in triplicates.

#### RNA sequencing analysis

Quality control of raw FASTQ files was conducted using FastQC. Adapters and low-quality reads were removed using Trimmomatic.^54^ Trimmed reads were aligned to the mouse reference genome (M35, GRCm39) using the splice-aware aligner HISAT2.^55^ The resulting BAM files of mapped reads were quantified against gene features using FeatureCounts with default parameters.^56^ Differentially expressed genes (DEGs) were identified using Limma-Voom,^57^ applying TMM normalization and defining DEGs as those with |log_2_ fold change| ≥ 1 and adjusted *p* value <0.05.

#### AS and RBP motif analysis

Aligned BAM files were used to conduct AS analysis using the rMATs-turbo suite.^31^ Statistical significance of AS events was considered at FDR <0.05 and |Ψ| > 0.2 to filter in DSGs. Splice motif analysis was conducted using rMAPs2.^32^

#### Gene ontology and functional enrichment analysis

DSGs were used for functional enrichment analyses. The output of rMATs was piped into rMAPs to generate RBP motif maps.^32^ DEGs and DSGs were analyzed for gene ontology (GO) and functional enrichment analysis using either David GO functional annotation tools or eVITTA’s pre-ranked gene set enrichment analysis (GSEA) or overrepresentation analysis (ORA) toolbox.^58^ Figures in this publication were made using bespoke scripts in Rstudio 4.4.0 ggplot or ggplot2 packages and extensions.

#### Cell viability assay

In a 96-well plastic plate, 1×10^4^ Mock^KD^, YA^KD^ or YB^KD^ were seeded in each well. Cells were cultured in each well with 200 μL of α-MEM supplemented with 10% FBS and 1% P/S. Cell viability was measured at 0, 24, 48, and 72 h. The plate was then incubated for 2 h at 37°C with each well containing 100 μL of α-MEM and 10 μL of Cell Counting Kit-8 solution (Dojindo Laboratories, Kumamoto, Japan). Absorbance was measured at 450 nm using a microplate reader (Sunrise Remote, Tecan, Männedorf, Switzerland). For experiments under NG or HG conditions with or without SRI-37330 hydrochloride (Selleck Chemicals), the culture medium was replaced with NG or HG medium containing SRI-37330 3 μM or vehicle control after cell attachment. Cell viability was assessed at the indicated time points.

#### polymerase chain reaction Real-time (qPCR)

RNeasy kit (QIAGEN) was used to extract total RNA from cells according to manufacturer’s instructions and Trizol-chloroform was used to extract RNA from bones. 1 femur was immersed in 1 mL Trizol and pulverized 5 times at 5000 RPM speed setting for 60 s each in TOMY Micro Smash MS 100 Cell Disruptor. Total RNA was used to synthesize cDNA using the SuperScript® IV First-Strand Synthesis System (Invitrogen, Carlsbad, CA, USA) according to manufacturer’s instructions. mRNA expression values were measured using the CFX96 Touch Real-Time PCR Detection System (Bio-Rad).

#### RNA immunoprecipitation (RIP) assay

RIP assay was performed using the EZ-Magna RIP kit (Millipore, Billerica, MA) according to the manufacturer’s instructions. Briefly, cells at 80% confluency were scraped off and lysed in RIP lysis buffer provided in the kit. Then 100 μL of whole cell lysates were incubated with RIP buffer containing magnetic beads conjugated with anti-HuR antibody (or normal IgG as negative control, Anti-SNRNP70 as positive control). After washing, RNA–protein complexes were eluted and digested with proteinase K, and the RNA was purified using the kit reagents. Purified RNA was subjected to cDNA synthesis and qPCR analysis as described in the RT-qPCR section.

#### mRNA stability assay

To estimate mRNA half-life, nascent transcription was blocked with Actinomycin D (ActD, final 10 μg/mL). At t = 0, 1, 2, 4, 6, 8 h after addition, cells were rapidly washed with ice-cold PBS and lysed in TRIzol and RT-qPCR was done as described. mRNA decay rate was measured by non-linear regression curve fitting (one phase decay) using GraphPad Prism using the following parameters: goodness of fit was quantified with R^2^, using ordinary fit at confidence level 95%.

#### Insulin response tests

Mock^KD^ or YB^KD^ cells were seeded in α-MEM supplemented with 10% FBS and 1% P/S until reaching 80% confluency. The media were then replaced with α-MEM containing 1% P/S and 1% FBS for 3 h followed a switch to the same media with or without 100 nM insulin (Sigma-Aldrich). After a 30-min incubation period, cells were harvested and protein extracted using radioimmunoprecipitation (RIPA) assay buffer (Millipore, Burlington, MA, USA) containing 1% protease and phosphatase inhibitor cocktail (Thermo Fisher Scientific) on ice for 20 min; insoluble material was separated by centrifugation at 14000 xg. Total protein was collected for western blot analysis.

#### Reactive oxygen species detection assay

Mock^KD^ or YB^KD^ (2.5 × 10^4^ cells per well) were seeded in a black, clear bottom 96-well microplate overnight. Intracellular reactive oxygen species (ROS) levels were measured using the DCFDA/H2DCFDA-Cellular ROS Assay Kit (Abcam, Cambridge, UK) according to manufacturer’s instructions. Cells were stained with 20 μM DCFDA in HBSS for 45 min at 37°C. Mock^KD^ or YB^KD^ cells treated with 50 μM TBHP for 4 h were used as positive control. DCF fluorescence intensity was measured using a fluorescence plate reader (Flex Station 3, Molecular Devices, San Jose, CA, USA) with excitation at 485 nm and emission at 535 nm.

#### Western blot

Cells were lysed using RIPA assay buffer (Millipore, Burlington) containing 1% protease and phosphatase inhibitor cocktail (Thermo Fisher Scientific) on ice for 20 min; insoluble material was separated by centrifugation 14000 xg. Protein from bones was extracted in RIPA buffer and processed similar to RNA extraction above. Total protein was quantified using Pierce BCA protein assay kit (Thermo Fisher Scientific). To prepare for SDS-PAGE gel electrophoresis, the protein was treated with β-Mercaptoethanol (Bio-Rad Laboratories) and Laemmli sample buffer (BioRad Laboratories) 3:1 and denatured at 95°C for 5 min. Equal protein amounts were loaded onto 4–15% Mini-PROTEAN TGX Precast Gels (Bio-Rad Laboratories) and transferred to a Trans-Blot Turbo Transfer System (Bio-Rad Laboratories) and then blocked with Block-Ace (DS Pharma Biomedical, Osaka, Japan) 1 h at room temperature (RT). Primary antibodies were incubated overnight at 4°C. The membranes were incubated with horseradish peroxidase-conjugated anti-rabbit antibody (Cell Signaling Technology, Danvers, MA, USA) at a dilution of 1:5,000 or anti-mouse antibody (GE Healthcare, Chicago, IL, USA) at a dilution of 1:10,000 for 1 h at RT after being washed in tris-buffered saline with Triton X-100 (TBS-T). Bound antibodies were detected with SuperSignalWest Femto Maximum Sensitivity Substrate (Thermo Fisher Scientific) and a FUSION-FX6 EDGE Chemiluminescence Imaging System (Vilber Lourmat, Collégien, France). Primary antibodies are in Table S5.

#### Confocal microscopy

1 × 10^5^ Mock^KD^, YA^KD^ or YB^KD^ cells were seeded in 35 mm glass bottom dish overnight. Cells were fixed with 4% formaldehyde for 15 min, then washed 3 times with ×1 PBS. Cells were permeabilized using 0.1% Triton X-100 for 10 min and blocked with 1.5% bovine serum albumin (BSA) for 30 min at RT. Cells were incubated with Alexa Fluor 568 Phalloidin (Invitrogen) for 1 h at RT. Afterward, cell nuclei were stained with 4′,6-diamidino-2-phenylindole, dihydrochloride (DAPI) (ThermoFisher Scientific) for 5 min at RT. For mitochondrial staining, 5 × 104 live cells were incubated with the MitoTracker Red CMXRos FM (Invitrogen) in a glass bottom dish for 45 min. Cells were washed with ×1 PBS 3 times and fixed with 4% formaldehyde for 15 min then washed by ×1 PBS. Cells were incubated for 10 min in ×1 PBS containing 0.1% Triton X-100 and stained with DAPI for 5 min at RT. Confocal images were acquired using a Zeiss LSM800 confocal laser scanning microscope (Carl Zeiss, Oberkochen, Germany). Confocal micrographs acquired with 10× and 20× objectives for phalloidin staining, and 40× for MitoTracker as a single optical slice.

#### Seahorse mitochondrial mito-stress test

Oxygen consumption rate (OCR) was measured using the Seahorse XF96 analyser (Agilent Technologies, Santa Clara, CA, USA) and Mito-stress test kit (Agilent Technologies) along with Seahorse XFe96/XF Pro FluxPak (Agilent Technologies) and Seahorse XF DMEM medium (without Phenol Red/pH 7.4/with HEPES/500 mL, Agilent Technologies). Initially, Mock^KD^ and YB^KD^ cells were seeded at 25,000 cells/well in 96-well plates on day one, while the XFe96 sensor cartridge hydrated in 200 μL calibration medium. On the next day, the cell medium was replaced with Seahorse XF DMEM, and cells were incubated at 37°C in a CO_2_-free incubator for 1 h. During this time, 1 μM oligomycin, 0.5 μM FCCP, antimycin A, and rotenone were loaded into the drug ports of the cartridge. After loading, the sensor plate was calibrated in the analyser. Following calibration, the cell culture plate was loaded, and the analysis initiated with the program: set: Mixture 2 min, Measure 3 min, 4 sets in all steps. Ports: (A: Oligomycin/B: FCCP/C: Antimycin/Rotenone).

#### Puromycin incorporation assay

Nascent protein synthesis was evaluated by puromycin incorporation assay. Cells were treated with 10 μg/mL puromycin (Sigma-Aldrich) for 30 min prior to harvest. Cell lysates were prepared and analyzed by western blot as previously described.

#### Pharmacological inhibition of TXNIP

To pharmacologically inhibit TXNIP, cells were treated with SRI-37330 hydrochloride purchased as a pre-dissolved stock solution in DMSO (10 mM). The compound was diluted in culture medium to a final concentration of 3 μM, and vehicle controls received the same volume of DMSO.

### Quantification and statistical analysis

All experiments were done in 3 or more replicates. Statistical analysis was performed using GraphPad Prism 9.0. Data were assessed for normality using the Shapiro–Wilk test. For datasets meeting normality assumptions, parametric tests including Student’s *t* test for two group comparisons and one-way ANOVA for multiple groups followed by Tukey’s post hoc test were applied. When data did not conform to a normal distribution, non-parametric tests were used including Mann–Whitney test for two-group comparisons and Kruskal–Wallis test for multiple-group comparisons followed by Dunn’s multiple comparison test. *p* values less than 0.05 were considered statistically significant. Significance levels are indicated as follows: ∗*p* < 0.05, ∗∗*p* < 0.01, ∗∗∗*p* < 0.001, and ∗∗∗∗*p* < 0.0001.

## Supplemental information

Document S1. Figures S1–S5

Table S1. Overrepresentation analysis of DSGs in primary osteocytes cultured in HG conditions

Table S2. Top 20 RNA-binding motifs around differentially spliced transcripts in primary osteocytes cultured in HG conditions

Table S3. Overrepresentation analysis of DEGs in after HuR KD

Table S4. DSGs, overrepresentation analysis of DSGs, and GOCC enrichment analysis after HuR KD

Table S5. Primer sequences, shRNA construct designs, and antibodies used for western blotting

Table S6. rMATS output from primary osteocytes cultured in HG conditions

Table S7. rMAPS output from primary osteocytes cultured in HG conditions

Table S8. rMATS output after HuR KD
