# Supplementary material for: Transcriptome-wide splicing analysis reveals HuR-regulated post-transcriptional programs in osteocytes
Source: iScience. 2026 Aug 10;29(8):116529. doi: 10.1016/j.isci.2026.116529 (PMC13486848; doi:10.1016/j.isci.2026.116529)

iScience, Volume *29*

Supplemental information

Transcriptome-wide splicing analysis reveals HuR-regulated post-transcriptional programs in osteocytes

Ziqiu Fan, Aseel Marahleh, Hideki Kitaura, Jiayi Ren, Abdulrahman Mousa, Fumitoshi Ohori, Kuniyasu Niizuma, Sherif Rashad, and Hiroyasu Kanetaka

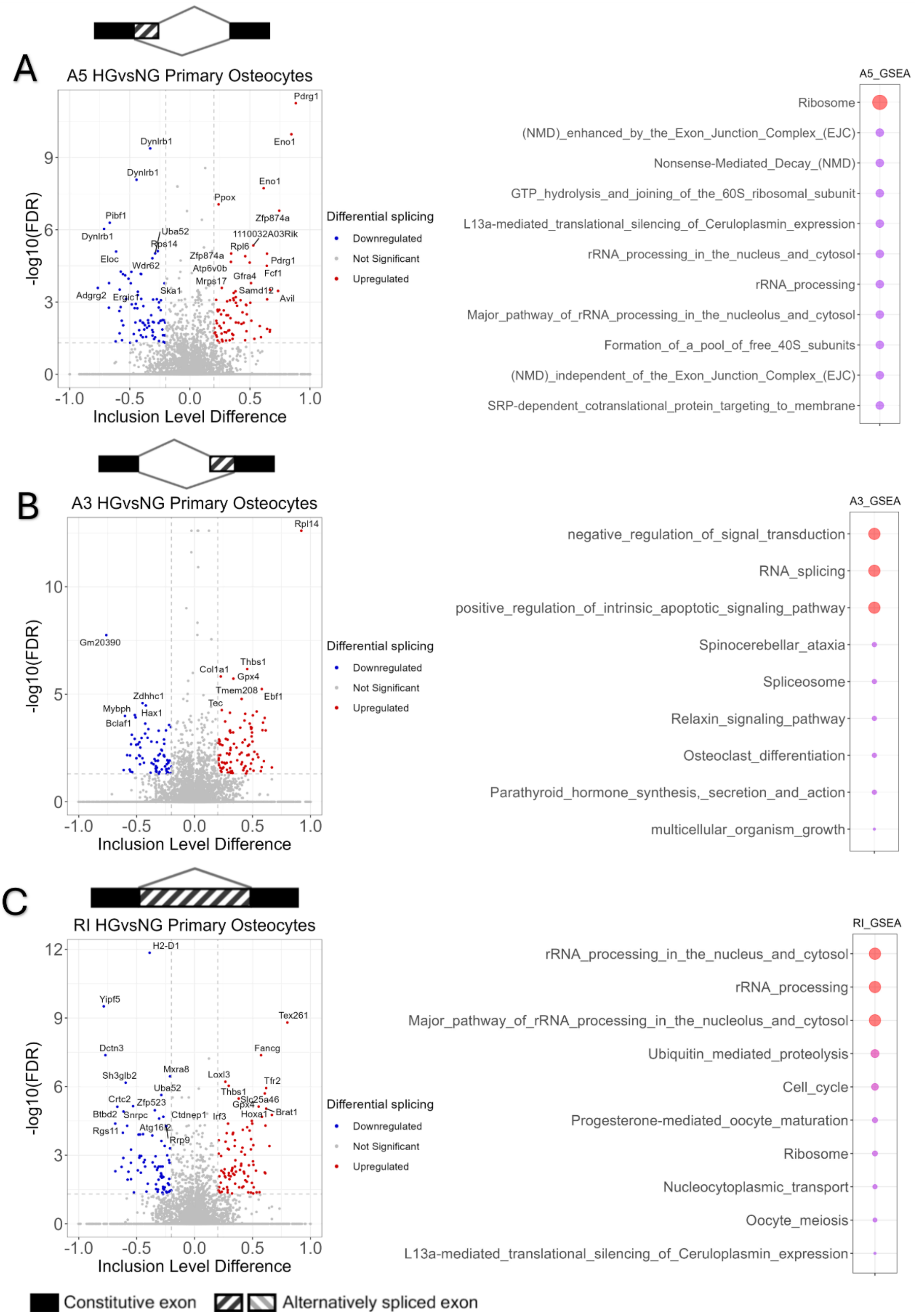
A
A5 HGvsNG Primary Osteocytes
-log10(FDR)
Inclusion Level Difference
Pdrg1
Dynlrb1
Eno1
Ppox
Pibf1
Zfp874a
Uba52
1110032A03Rik
Rps14
Rpl6
Eloc
Zfp874a
Pdrg1
Wdr62
Atp6v0b
Fcf1
Gfra4
Mrps17
Ska1
Samd12
Adgrg2
Ergic1
Avil
Differential splicing
Downregulated
Not Significant
Upregulated
A5_GSEA
Ribosome
(NMD)_enhanced_by_the_Exon_Junction_Complex_(EJC)
Nonsense-Mediated_Decay_(NMD)
GTP_hydrolysis_and_joining_of_the_60S_ribosomal_subunit
L13a-mediated_translational_silencing_of_Ceruloplasmin_expression
rRNA_processing_in_the_nucleus_and_cytosol
rRNA_processing
Major_pathway_of_rRNA_processing_in_the_nucleolus_and_cytosol
Formation_of_a_pool_of_free_40S_subunits
(NMD)_independent_of_the_Exon_Junction_Complex_(EJC)
SRP-dependent_cotranslational_protein_targeting_to_membrane
B
A3 HGvsNG Primary Osteocytes
-log10(FDR)
Inclusion Level Difference
Rpl14
Gm20390
Thbs1
Col1a1
Gpx4
Tmem208
Zdhhc1
Ebf1
Tec
Mybph
Hax1
Bclaf1
Differential splicing
Downregulated
Not Significant
Upregulated
A3_GSEA
negative_regulation_of_signal_transduction
RNA_splicing
positive_regulation_of_intrinsic_apoptotic_signaling_pathway
Spinocerebellar_ataxia
Spliceosome
Relaxin_signaling_pathway
Osteoclast_differentiation
Parathyroid_hormone_synthesis,_secretion_and_action
multicellular_organism_growth
C
RI HGvsNG Primary Osteocytes
-log10(FDR)
Inclusion Level Difference
H2-D1
Yipf5
Tex261
Dctn3
Fancg
Mxra8
Sh3glb2
Loxl3
Tfr2
Uba52
Thbs1
Crtc2
Zfp523
Slc25a46
Gpx4
Btbd2
Snrpc
Ctdnep1
Irf3
Hoxa1
Brat1
Rgs11
Atg16l2
Rrp9
Differential splicing
Downregulated
Not Significant
Upregulated
RI_GSEA
rRNA_processing_in_the_nucleus_and_cytosol
rRNA_processing
Major_pathway_of_rRNA_processing_in_the_nucleolus_and_cytosol
Ubiquitin_mediated_proteolysis
Cell_cycle
Progesterone-mediated_oocyte_maturation
Ribosome
Nucleocytoplasmic_transport
Oocyte_meiosis
L13a-mediated_translational_silencing_of_Ceruloplasmin_expression
Constitutive exon
Alternatively spliced exon

Supplementary Fig 1: High glucose induces extensive AS changes in primary osteocytes. Related to Figure 1.

(A-C) Volcano plots and overrepresentation analysis of DSGs around AS events showing inclusion level (ΔΨ) difference comparing 5.5mM and 25mM glucose at FDR < 0.05 and 0.2 < ΔΨ < -0.2 in primary osteocytes. Overrepresentation analysis was done through the eVITTA web-based toolbox. All analyses were done on n=3.

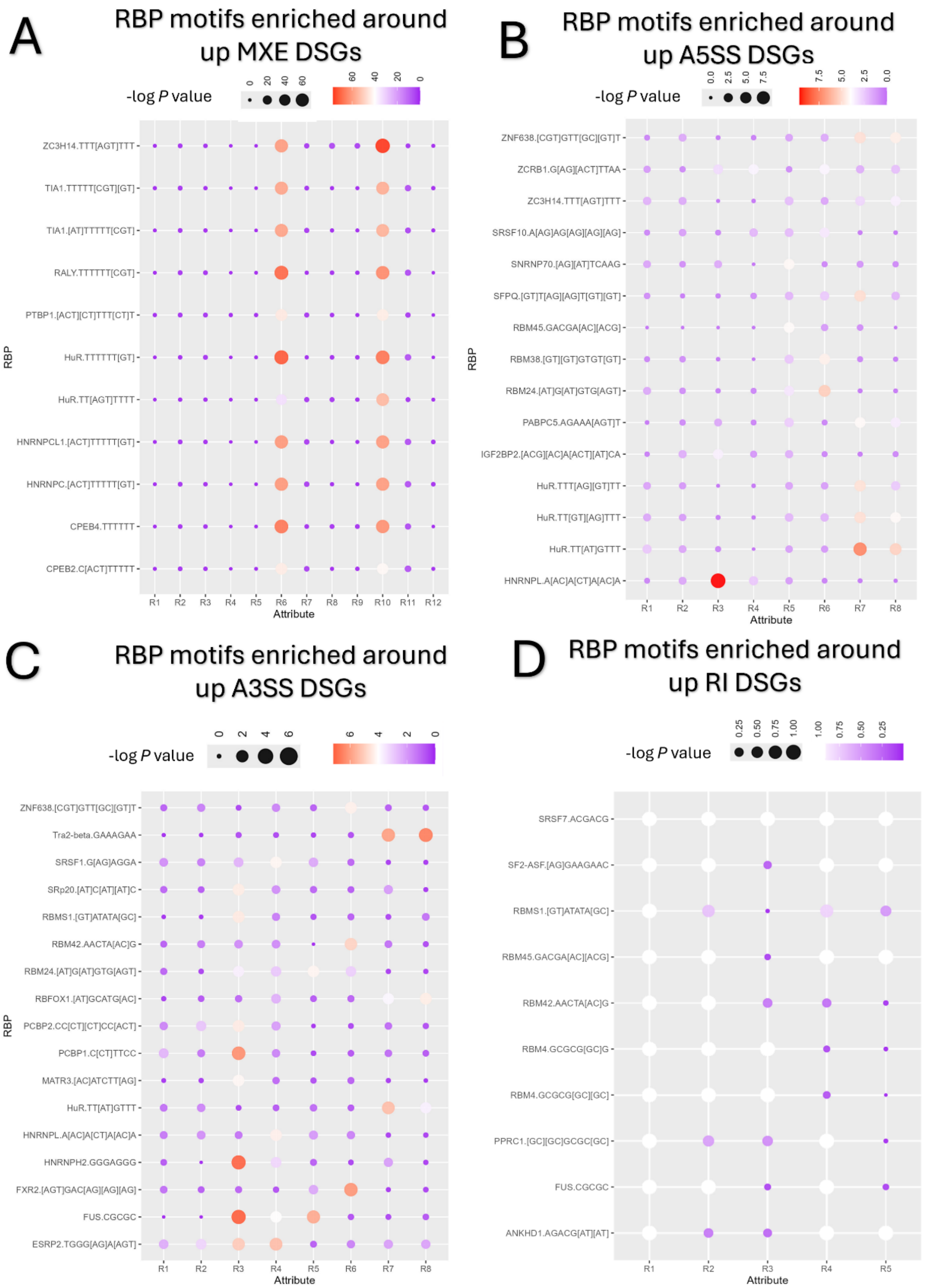

A
RBP motifs enriched around up MXE DSGs
-log P value
0 20 40 60
60 40 20 0
RBP
ZC3H14.TTT[AGT]TTT
TIA1.TTTTT[CGT][GT]
TIA1.[AT]TTTTT[CGT]
RALY.TTTTTT[CGT]
PTBP1.[ACT][CT]TTT[CT]T
HuR.TTTTTT[GT]
HuR.TT[AGT]TTTT
HNRNPCL1.[ACT]TTTTT[GT]
HNRNPC.[ACT]TTTTT[GT]
CPEB4.TTTTTT
CPEB2.C[ACT]TTTTT
R1 R2 R3 R4 R5 R6 R7 R8 R9 R10 R11 R12
Attribute
B
RBP motifs enriched around up A5SS DSGs
-log P value
0.0 2.5 5.0 7.5
7.5 5.0 2.5 0.0
RBP
ZNF638.[CGT]GTT[GC][GT]T
ZCRB1.G[AG][ACT]TTAA
ZC3H14.TTT[AGT]TTT
SRSF10.A[AG]AG[AG][AG][AG]
SNRNP70.[AG][AT]TCAAG
SFPQ.[GT]T[AG][AG]T[GT][GT]
RBM45.GACGA[AC][ACG]
RBM38.[GT][GT]GTGT[GT]
RBM24.[AT]G[AT]GTG[AGT]
PABPC5.AGAAA[AGT]T
IGF2BP2.[ACG][AC]A[ACT][AT]CA
HuR.TTT[AG][GT]TT
HuR.TT[GT][AG]TTT
HuR.TT[AT]GTTT
HNRNPL.A[AC]A[CT]A[AC]A
R1 R2 R3 R4 R5 R6 R7 R8
Attribute
C
RBP motifs enriched around up A3SS DSGs
-log P value
0 2 4 6
6 4 2 0
RBP
ZNF638.[CGT]GTT[GC][GT]T
Tra2-beta.GAAAGAA
SRSF1.G[AG]AGGA
SRp20.[AT]C[AT][AT]C
RBMS1.[GT]ATATA[GC]
RBM42.AACTA[AC]G
RBM24.[AT]G[AT]GTG[AGT]
RBFOX1.[AT]GCATG[AC]
PCBP2.CC[CT][CT]CC[ACT]
PCBP1.C[CT]TTCC
MATR3.[AC]ATCTT[AG]
HuR.TT[AT]GTTT
HNRNPL.A[AC]A[CT]A[AC]A
HNRNPH2.GGGAGGG
FXR2.[AGT]GAC[AG][AG][AG]
FUS.CGCGC
ESRP2.TGGG[AG]A[AGT]
R1 R2 R3 R4 R5 R6 R7 R8
Attribute
D
RBP motifs enriched around up RI DSGs
-log P value
0.25 0.50 0.75 1.00
1.00 0.75 0.50 0.25
SRSF7.ACGACG
SF2-ASF.[AG]GAAGAAC
RBMS1.[GT]ATATA[GC]
RBM45.GACGA[AC][ACG]
RBM42.AACTA[AC]G
RBM4.GCGCG[GC]G
RBM4.GCGCG[GC][GC]
PPRC1.[GC][GC]GCGC[GC]
FUS.CGCGC
ANKHD1.AGACG[AT][AT]
R1 R2 R3 R4 R5
Attribute

Supplementary Fig 2: Significantly up-enriched RBPs motifs around AS events. Related to Figure 2.

(A-D) The x axis (Attribute) represents the position (R) of each splicing event in 5' → 3'. RBP binding motifs are described in the y axis. The panels show MXE, A5SS, A3SS and RI RBP motifs around up and down regulated DSGs and bubble size and color denote the – log10 P value.

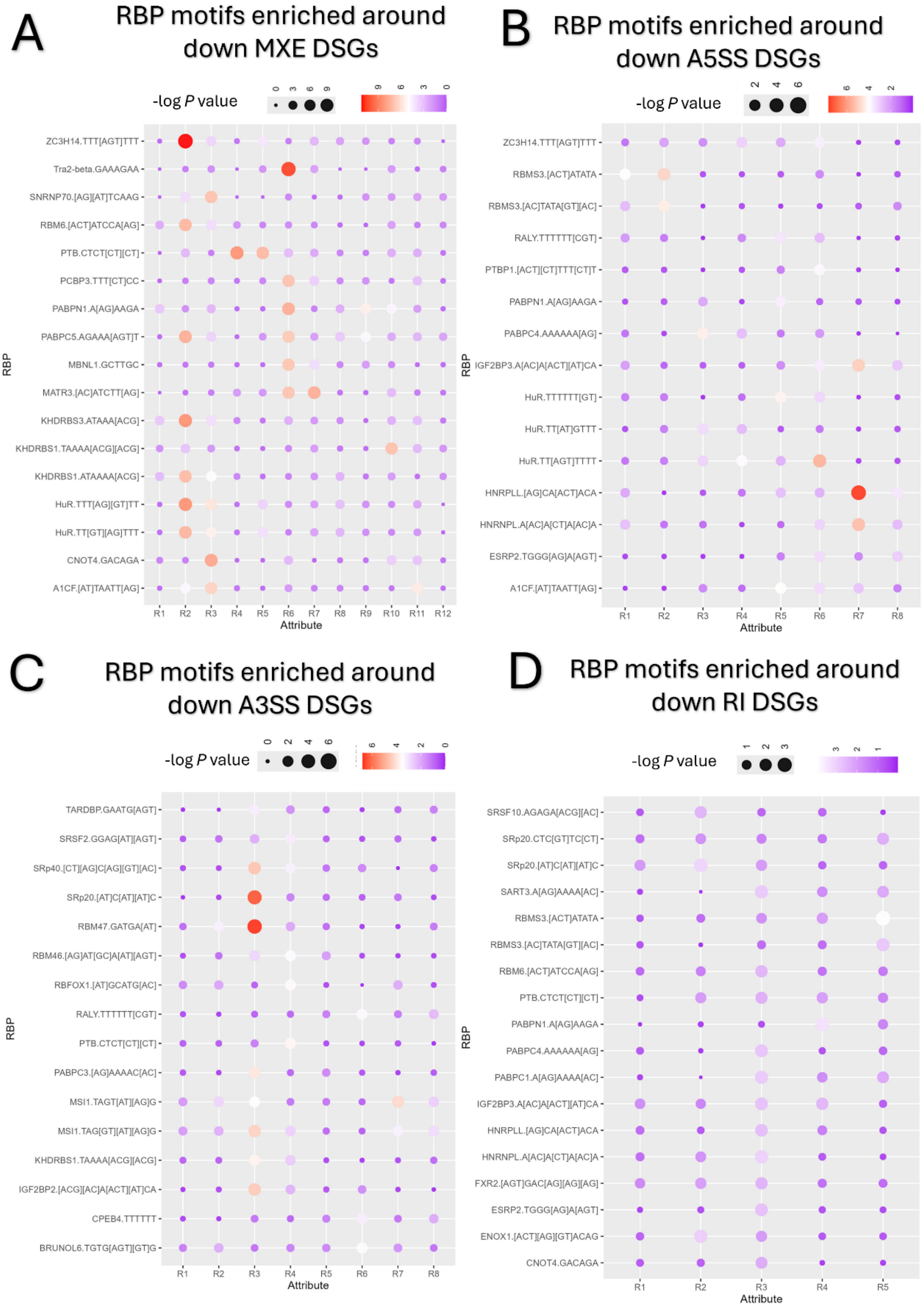
A
RBP motifs enriched around down MXE DSGs
-log P value
ZC3H14.TTT[AGT]TTT
Tra2-beta.GAAAGAA
SNRNP70.[AG][AT]TCAAG
RBM6.[ACT]ATCCA[AG]
PTB.CTCT[CT][CT]
PCBP3.TTT[CT]CC
PABPN1.A[AG]AAGA
PABPC5.AGAAA[AGT]T
MBNL1.GCTTGC
MATR3.[AC]ATCTT[AG]
KHDRBS3.ATAAA[ACG]
KHDRBS1.TAAAA[ACG][ACG]
KHDRBS1.ATAAAA[ACG]
HuR.TTT[AG][GT]TT
HuR.TT[GT][AG]TTT
CNOT4.GACAGA
A1CF.[AT]TAATT[AG]
RBP
Attribute
B
RBP motifs enriched around down A5SS DSGs
-log P value
ZC3H14.TTT[AGT]TTT
RBMS3.[ACT]ATATA
RBMS3.[AC]TATA[GT][AC]
RALY.TTTTTT[CGT]
PTBP1.[ACT][CT]TTT[CT]T
PABPN1.A[AG]AAGA
PABPC4.AAAAAA[AG]
IGF2BP3.A[AC]A[ACT][AT]CA
HuR.TTTTTT[GT]
HuR.TT[AT]GTTT
HuR.TT[AGT]TTTT
HNRPLL.[AG]CA[ACT]ACA
HNRNPL.A[AC]A[CT]A[AC]A
ESRP2.TGGG[AG]A[AGT]
A1CF.[AT]TAATT[AG]
RBP
Attribute
C
RBP motifs enriched around down A3SS DSGs
-log P value
TARDBP.GAATG[AGT]
SRSF2.GGAG[AT][AGT]
SRp40.[CT][AG]C[AG][GT][AC]
SRp20.[AT]C[AT][AT]C
RBM47.GATGA[AT]
RBM46.[AG]AT[GC]A[AT][AGT]
RBFOX1.[AT]GCATG[AC]
RALY.TTTTTT[CGT]
PTB.CTCT[CT][CT]
PABPC3.[AG]AAAAC[AC]
MSI1.TAGT[AT][AG]G
MSI1.TAG[GT][AT][AG]G
KHDRBS1.TAAAA[ACG][ACG]
IGF2BP2.[ACG][AC]A[ACT][AT]CA
CPEB4.TTTTTT
BRUNOL6.TGTG[AGT][GT]G
RBP
Attribute
D
RBP motifs enriched around down RI DSGs
-log P value
SRSF10.AGAGA[ACG][AC]
SRp20.CTC[GT]TC[CT]
SRp20.[AT]C[AT][AT]C
SART3.A[AG]AAAA[AC]
RBMS3.[ACT]ATATA
RBMS3.[AC]TATA[GT][AC]
RBM6.[ACT]ATCCA[AG]
PTB.CTCT[CT][CT]
PABPN1.A[AG]AAGA
PABPC4.AAAAAA[AG]
PABPC1.A[AG]AAAA[AC]
IGF2BP3.A[AC]A[ACT][AT]CA
HNRPLL.[AG]CA[ACT]ACA
HNRNPL.A[AC]A[CT]A[AC]A
FXR2.[AGT]GAC[AG][AG][AG]
ESRP2.TGGG[AG]A[AGT]
ENOX1.[ACT][AG][GT]ACAG
CNOT4.GACAGA
RBP
Attribute

Supplementary Fig 3: Significantly down-enriched RBPs motifs around AS events. Related to Figure 2.

(A-D) The x axis (Attribute) represents the position (R) of each splicing event in 5’ → 3’. RBP binding motifs are described in the y axis. The panels show MXE, A5SS, A3SS and RI RBP motifs around up and down regulated DSGs and bubble size and colour denote the – log10 P value.

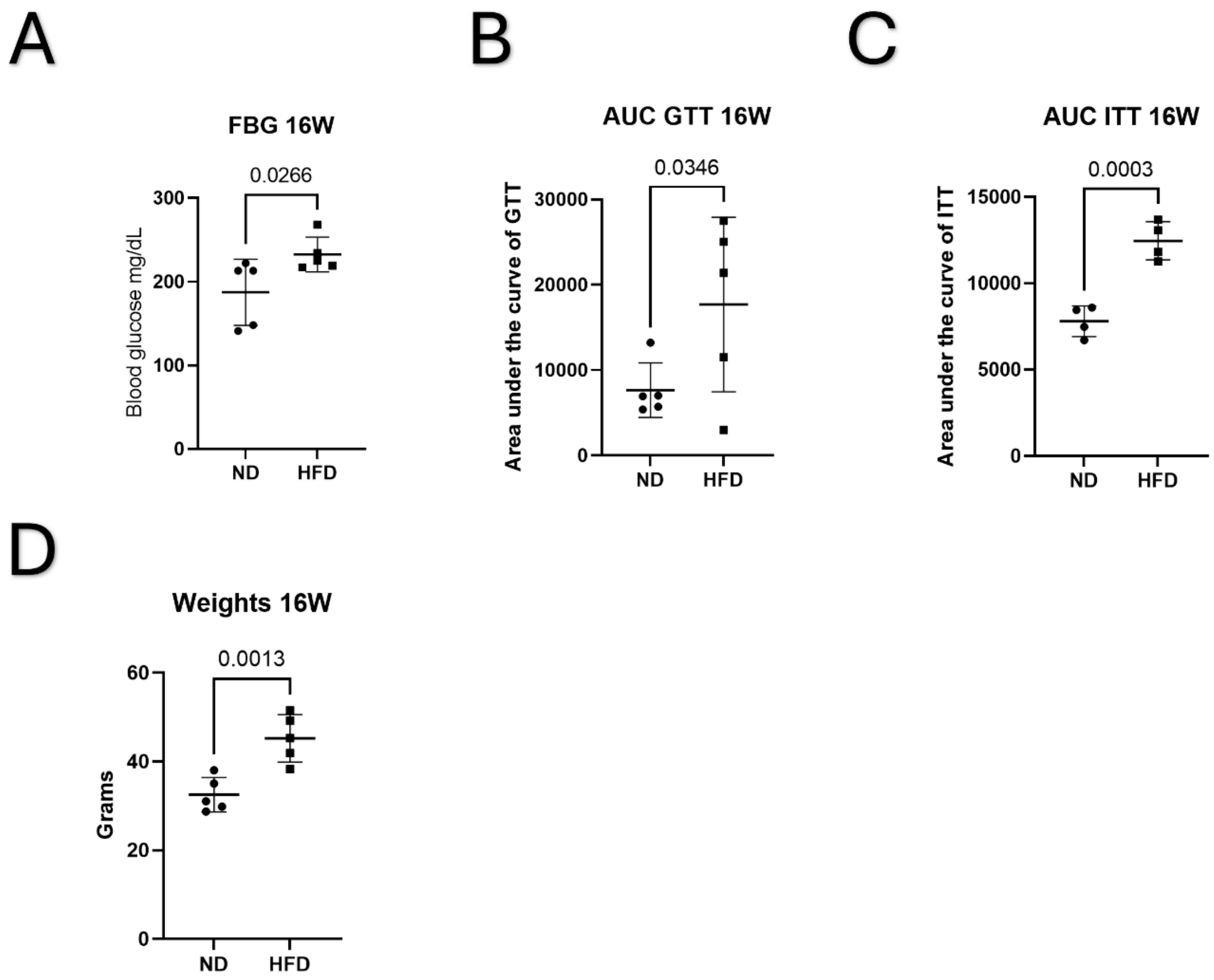

Supplementary Fig 4: Weight and metabolic tests of mice fed a high fat diet for 16 weeks. Related to Figure 3 and Star Methods.

(A) Six-hour fasted blood glucose levels of mice fed a ND or HFD. Data are presented as mean ± SD, n=5.

(B) Glucose tolerance test area under the curve (AUC) calculated from individual glucose tolerance tests of mice fed a ND or HFD. Data are presented as mean ±SD, n=5.

(C) Insulin tolerance test area under the curve (AUC) calculated from individual insulin tolerance tests of mice fed a ND or HFD. Data are presented as mean ±SD, n=4.

(D) Weight in grams of mice fed a ND or HFD Data are presented as mean ± SD, n=5.

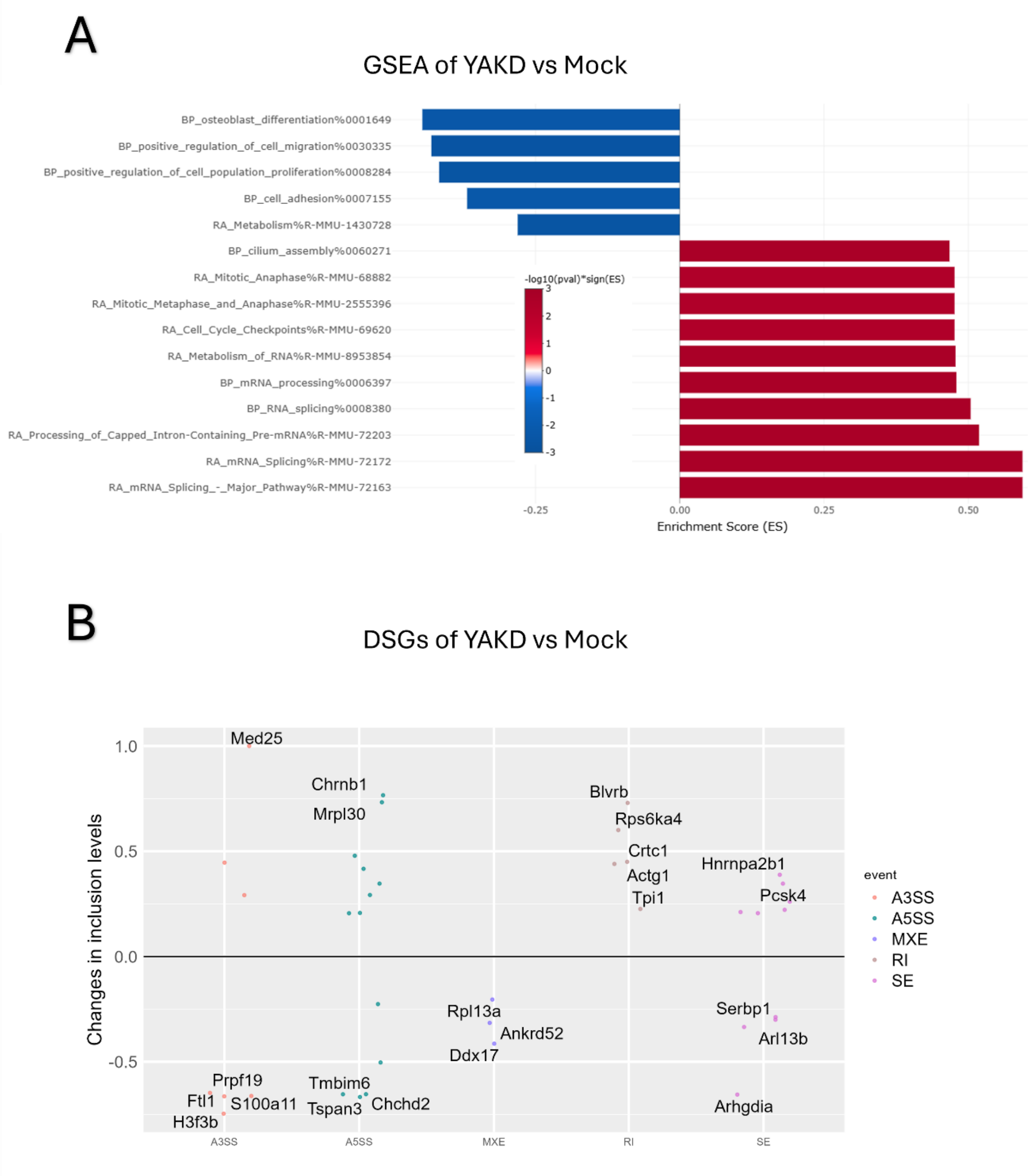

Supplementary Fig 5: Alternative HuR KD (YA^{KD}) construct enrichment analysis suggests off target effects. Related to Figure 4 and Figure 6.

(A) Gene set enrichment analysis using eVITTA toolbox after YA^{KD} of DEGs at LogFC 1 and -log10 adjusted p<0.05. Enriched terms include splicing and RNA metabolism as well as DNA damage repair, cell checkpoint activation and reduced cell adhension and proliferation.

(B) Scatter plot showing DSGs around AS in YA^{KD} events in 5 patterns showing almost no differentially spliced genes at FDR < 0.05 and $0.2 < \Delta\Psi < -0.2$.

Data S1: Full western blot images of representative immunoblot panels in this study.

Figure 3C

HuR (36kDa)

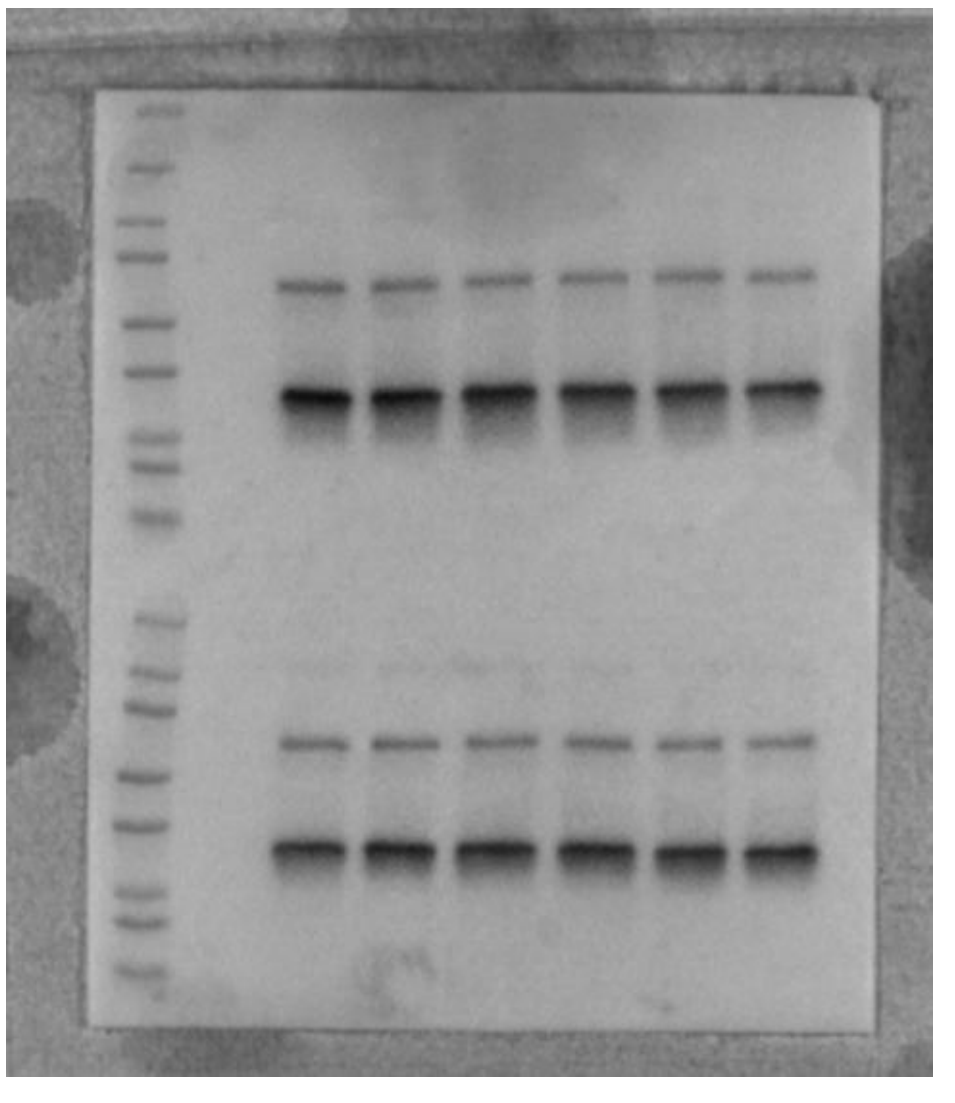

β-actin (42kDa)

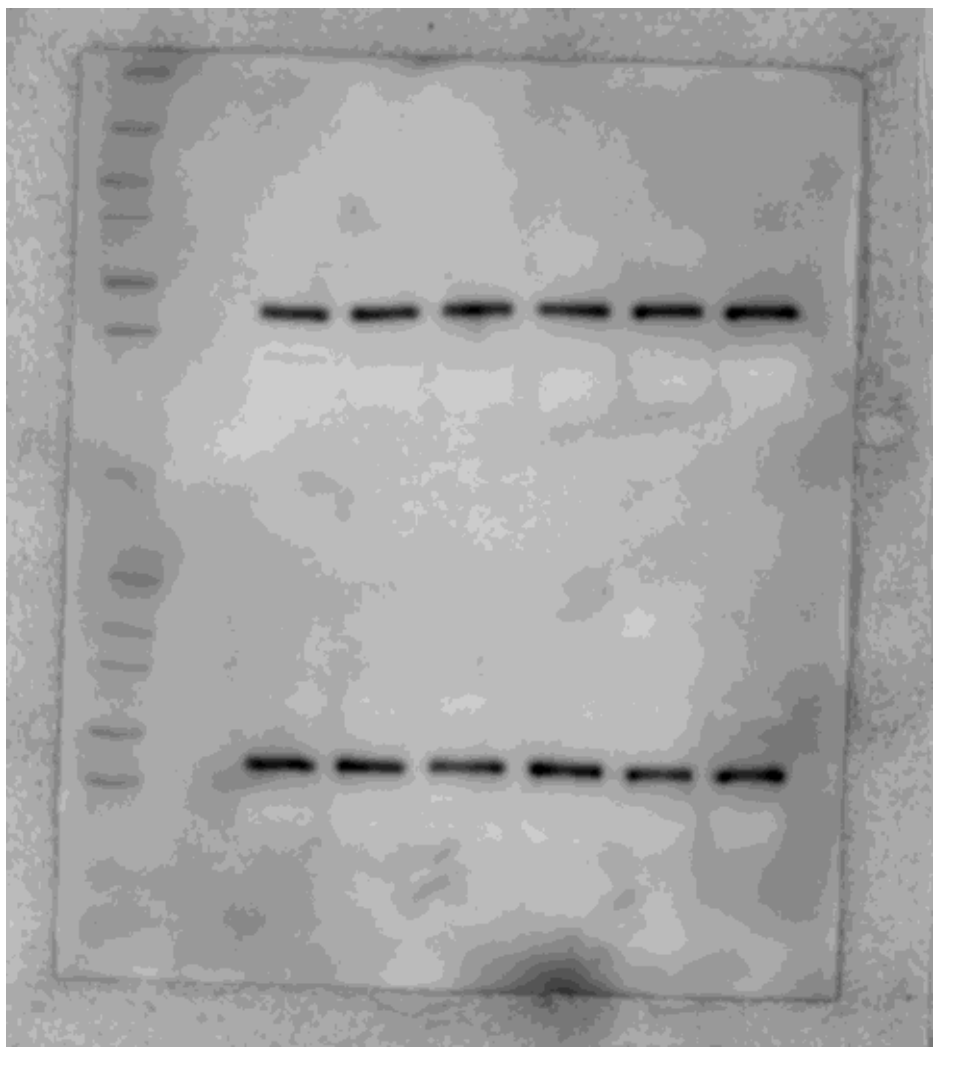

Figure 3F

HuR (36kDa)

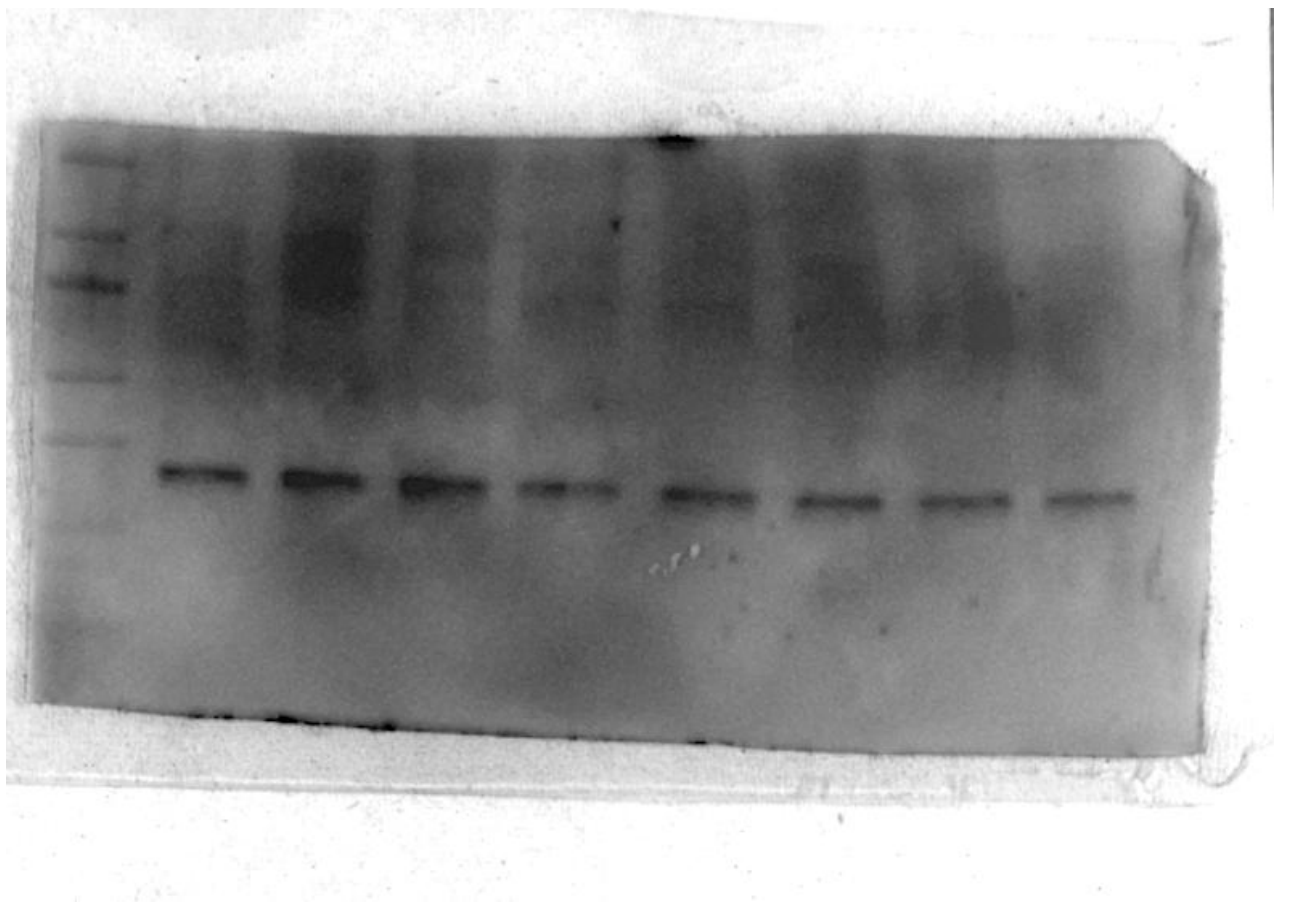

β-actin (42kDa)

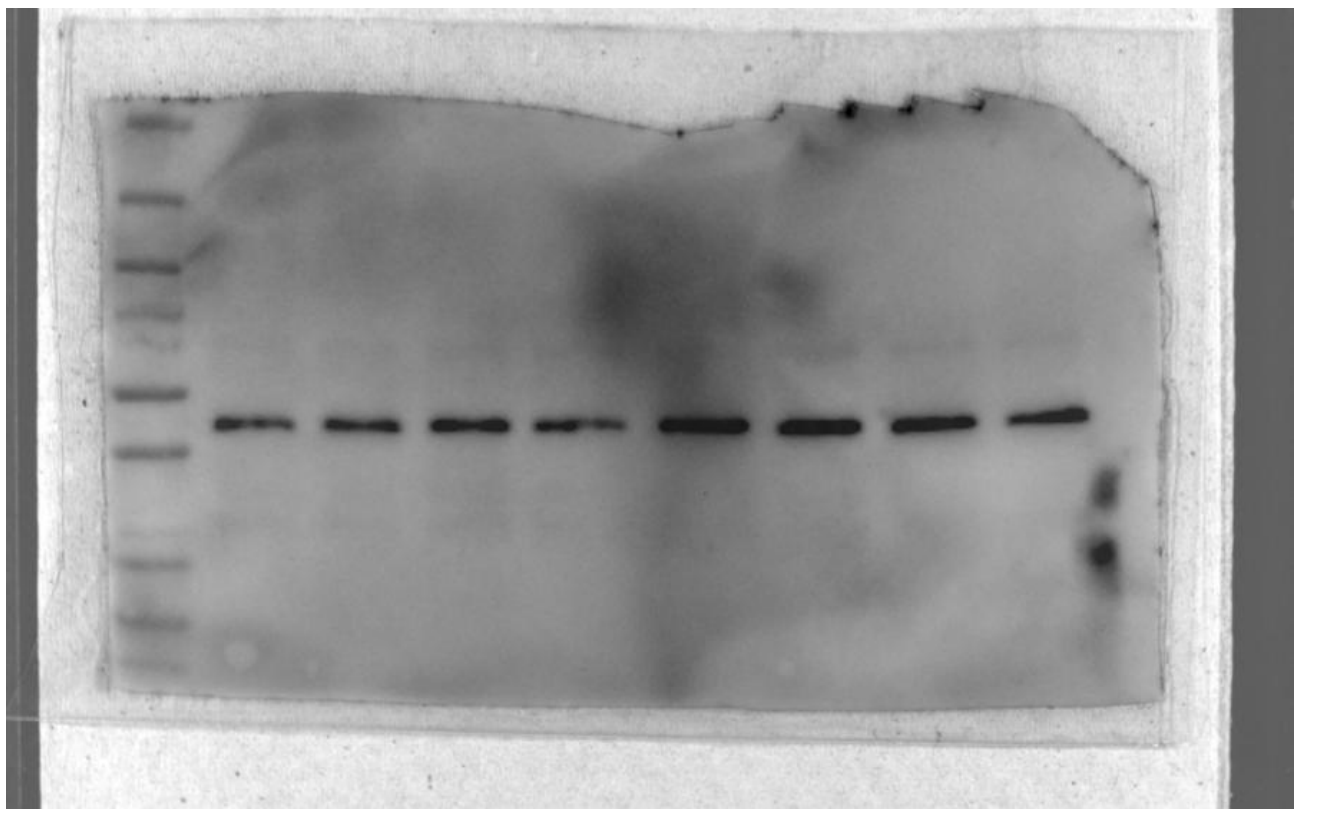

Figure 4A

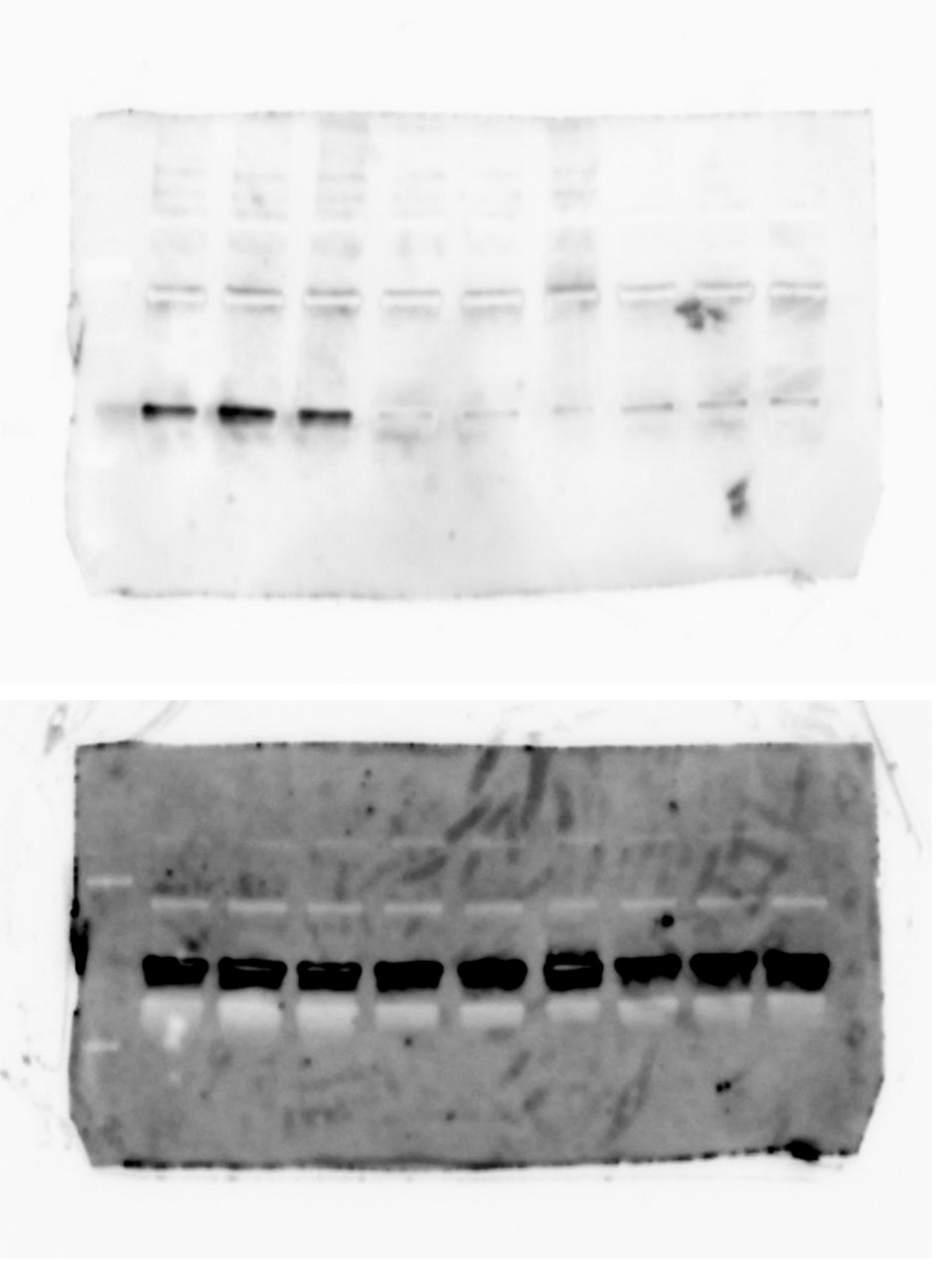

HuR (36kDa)

β-actin (42kDa)

Figure 7A

p-p70s6k(70kDa)

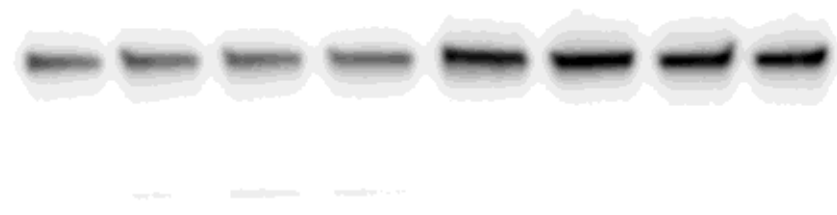

p70s6k (70kDa)

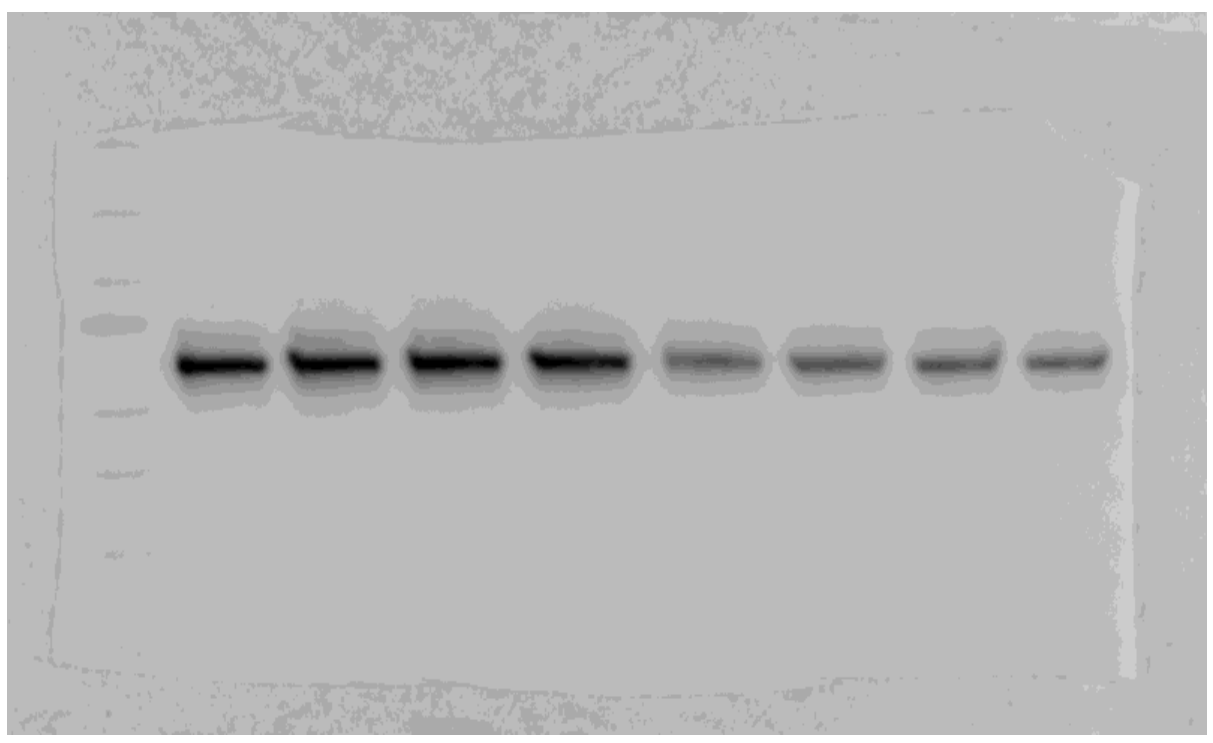

β-actin (42kDa)

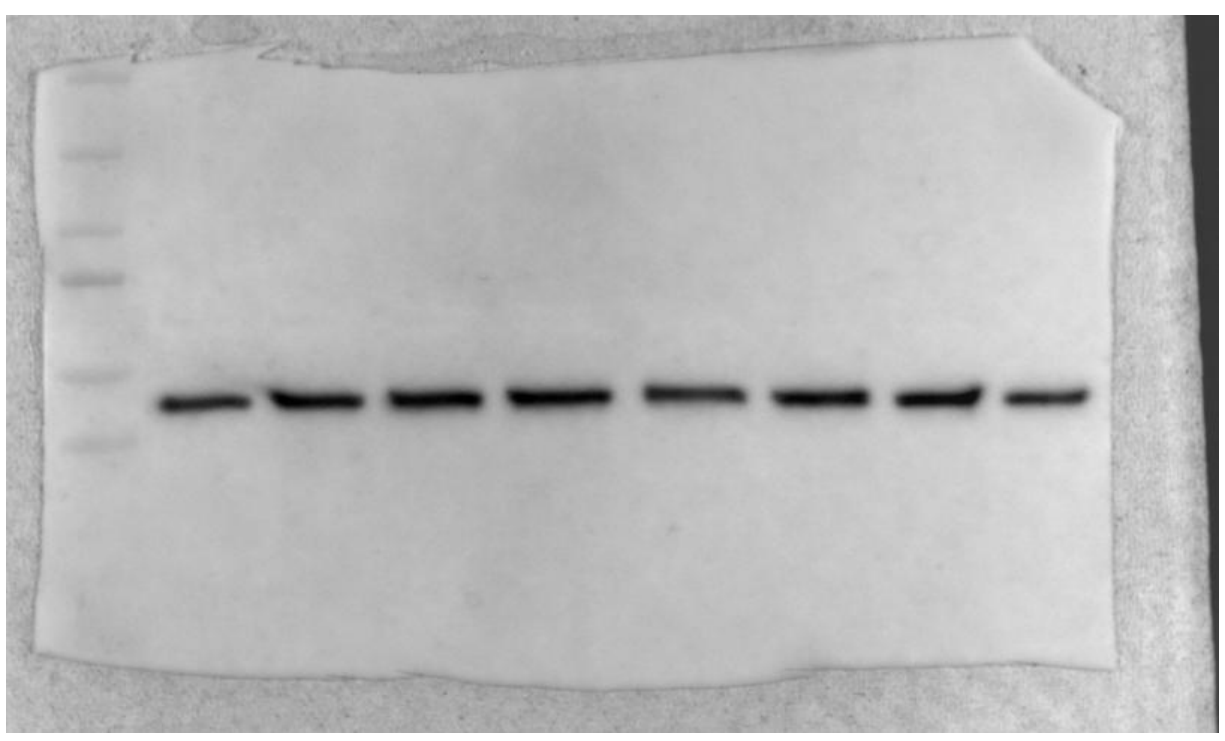

Figure 7B

p-4E-BP1 (15-20kDa)

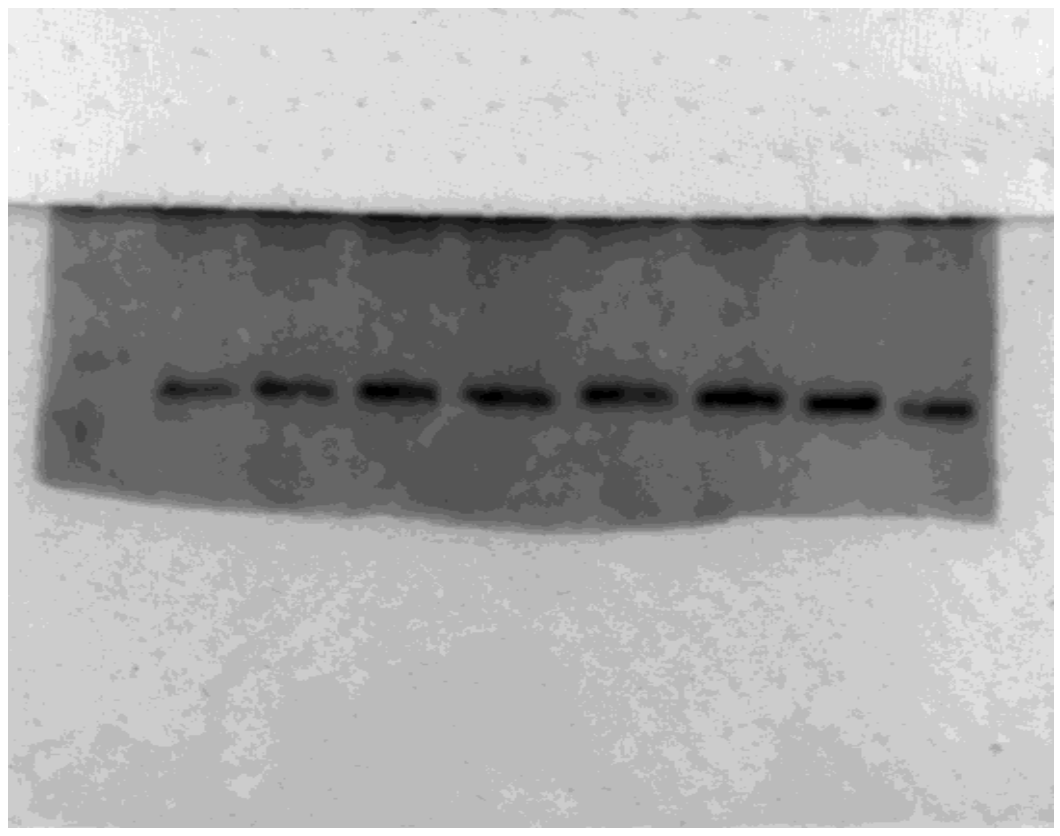

4E-BP1 (15-20kDa)

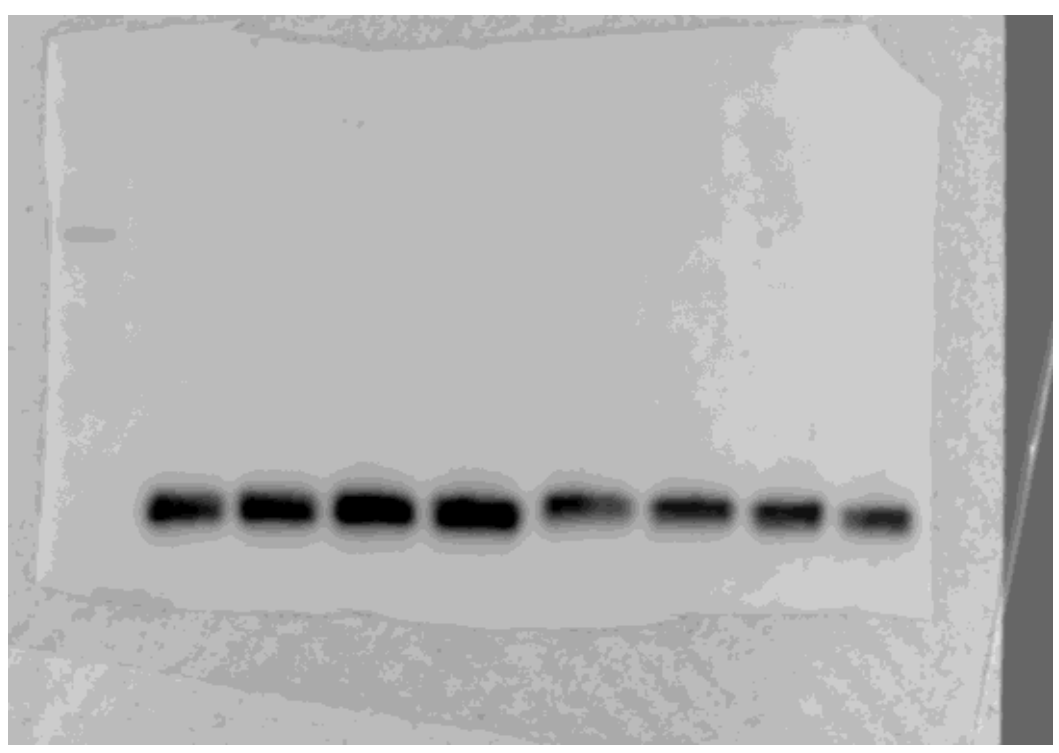

β-actin (42kDa)

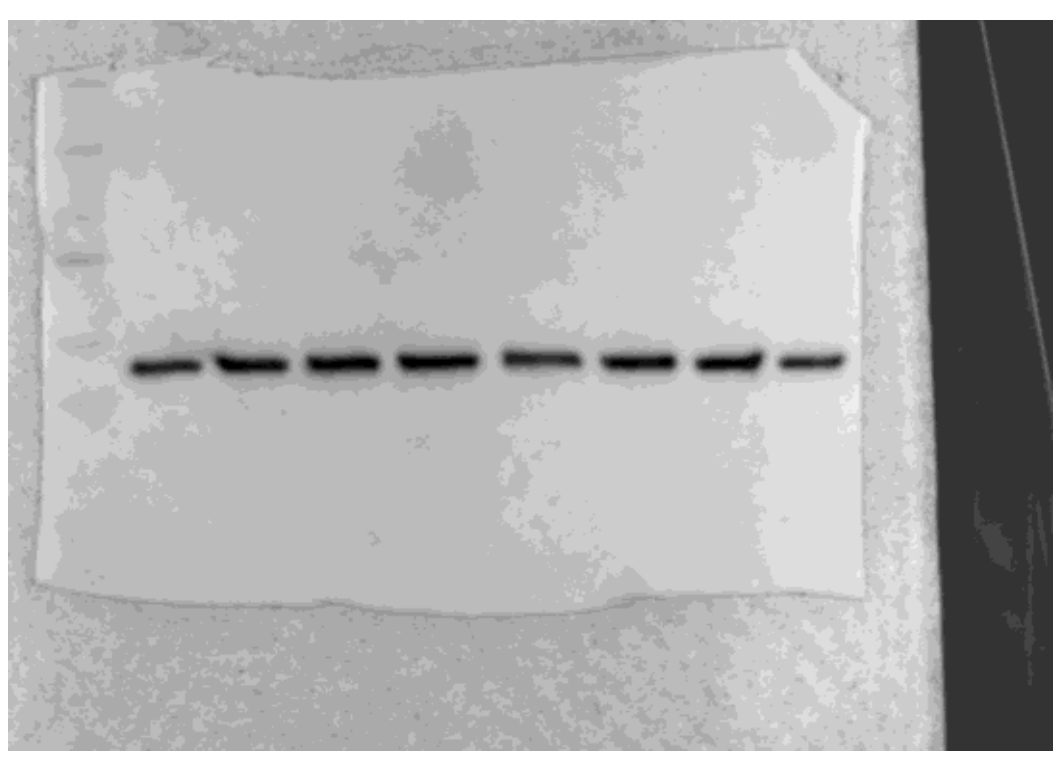

Figure 7C

p-MNK1 (50kDa)

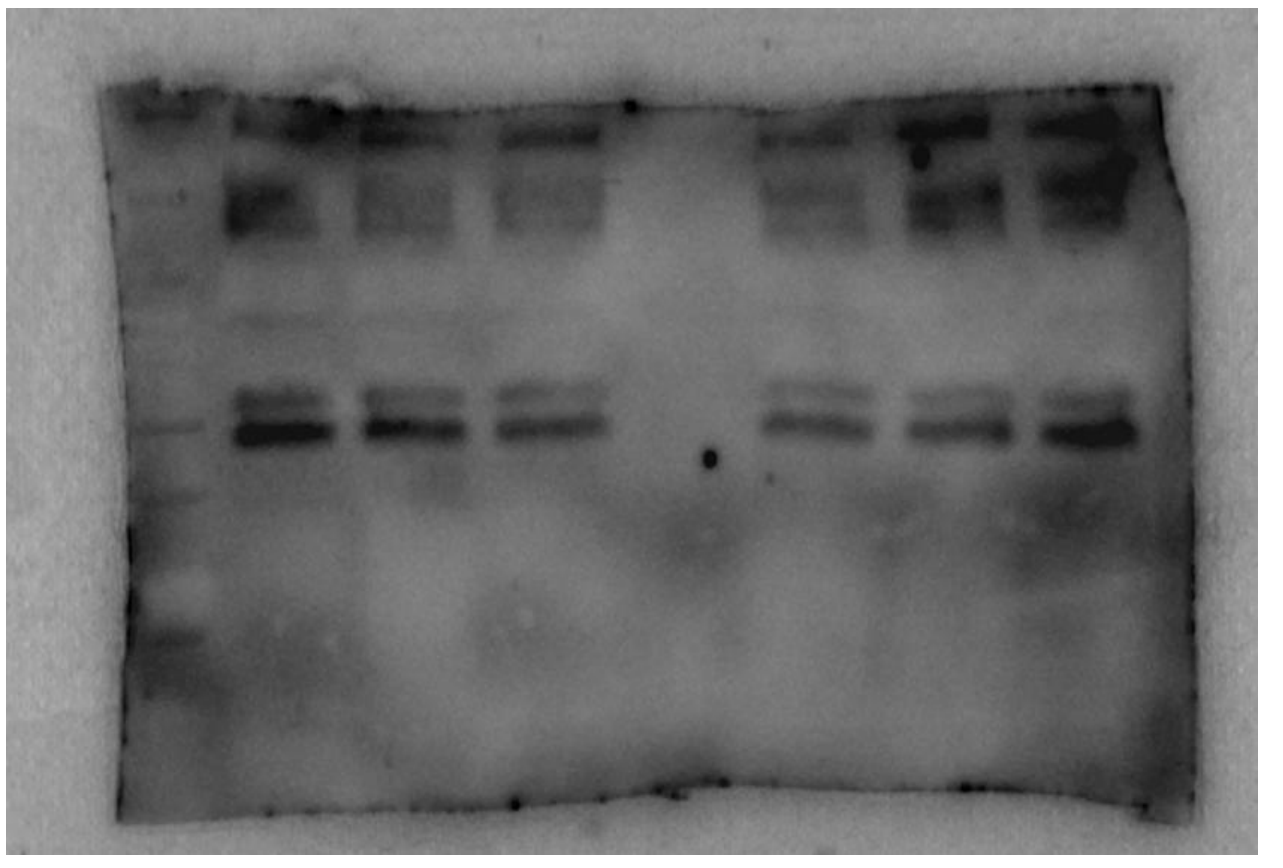

MNK1 (50kDa)

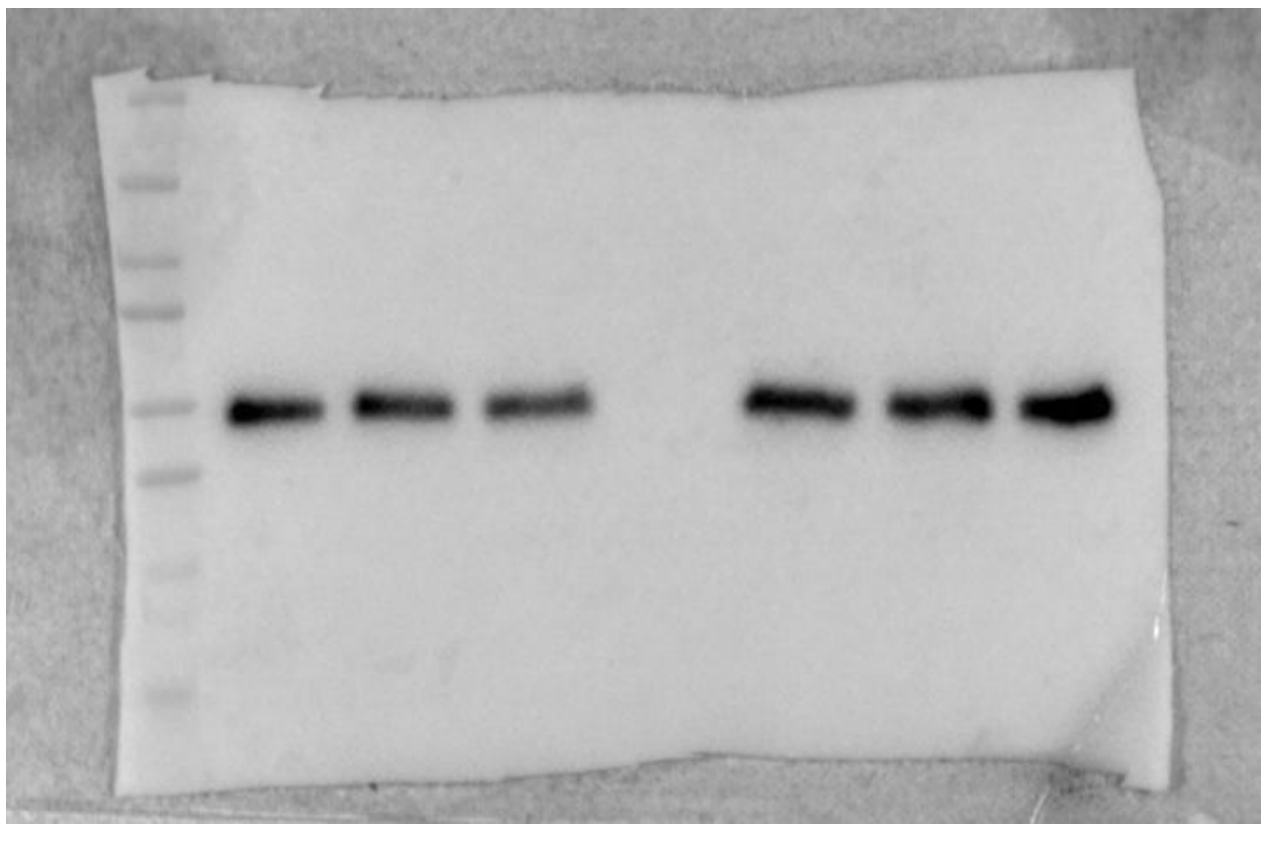

β-actin (42kDa)

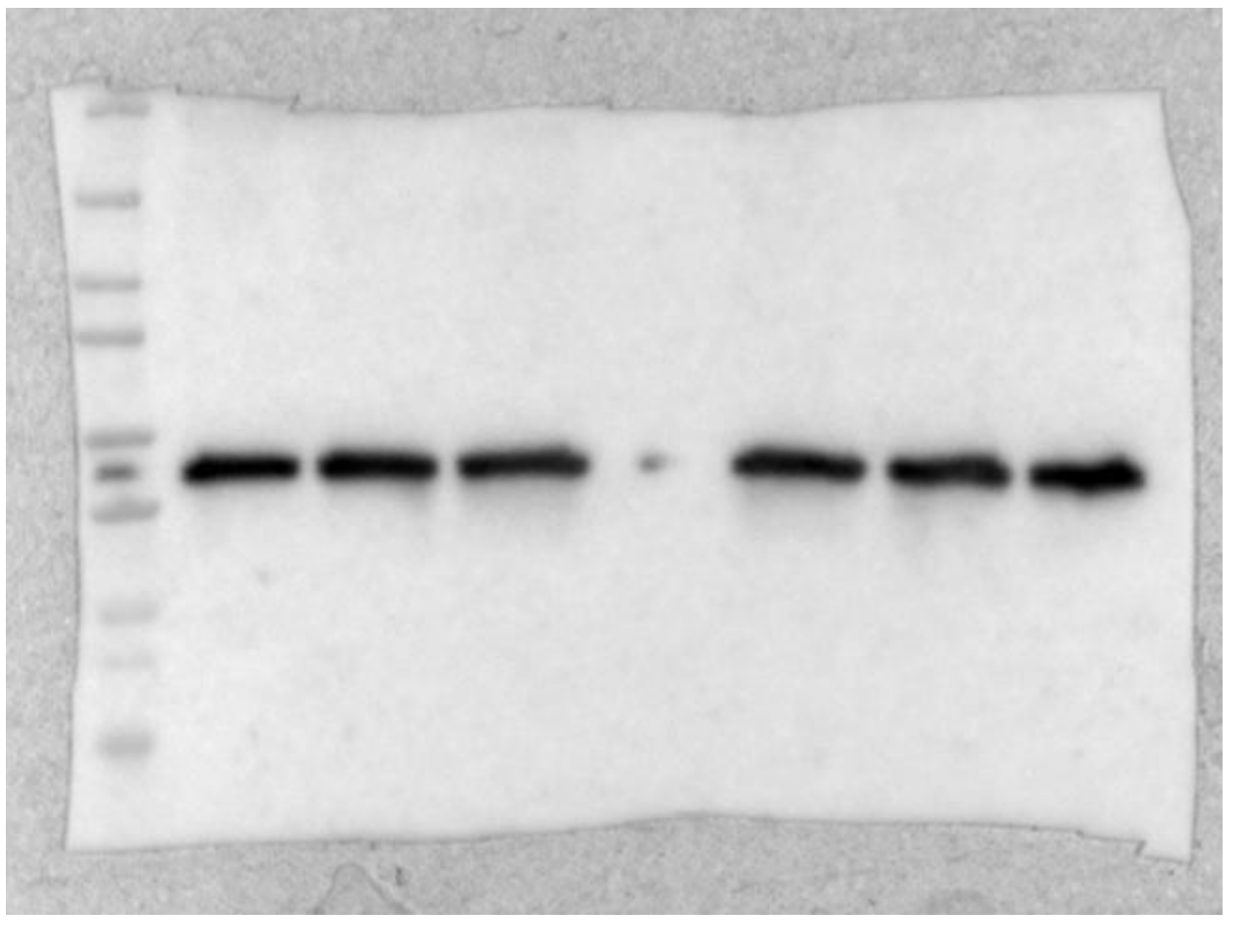

Figure 7D

p-ERK1/2 (42, 44k Da)

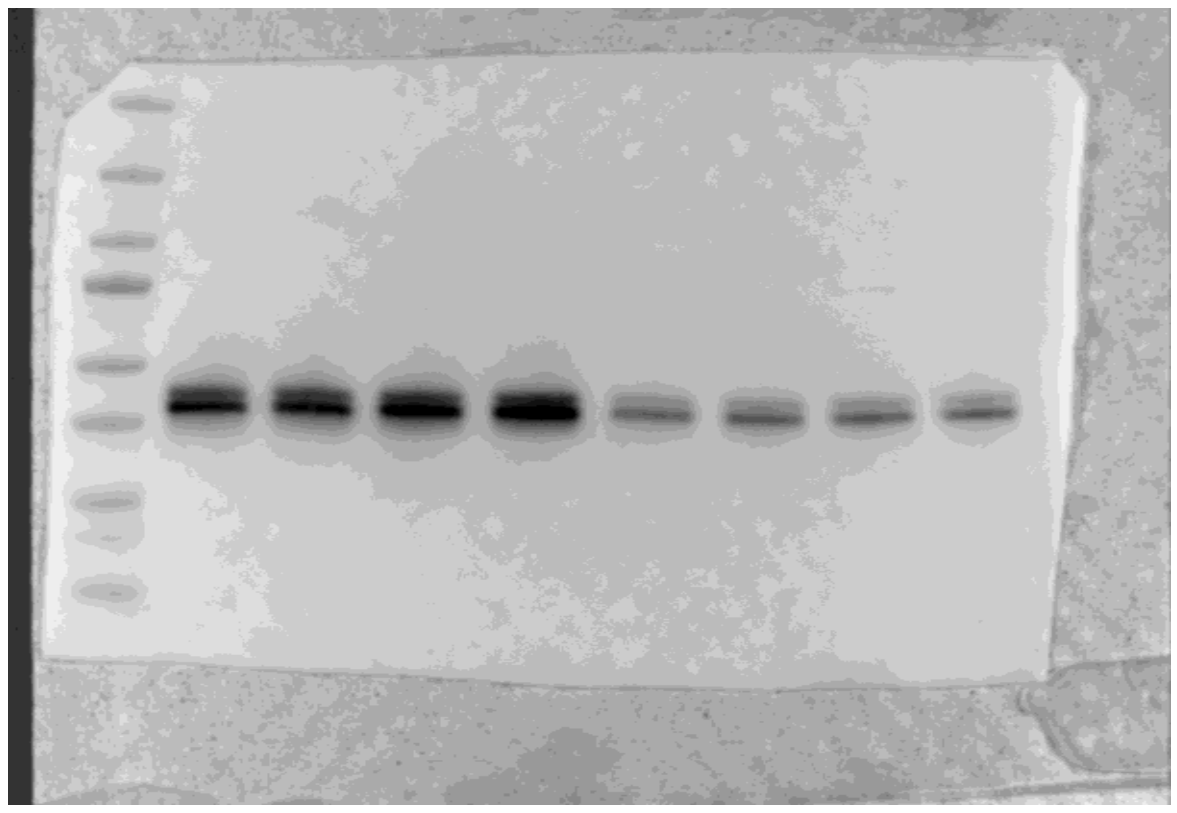

ERK1/2 (42, 44k Da)

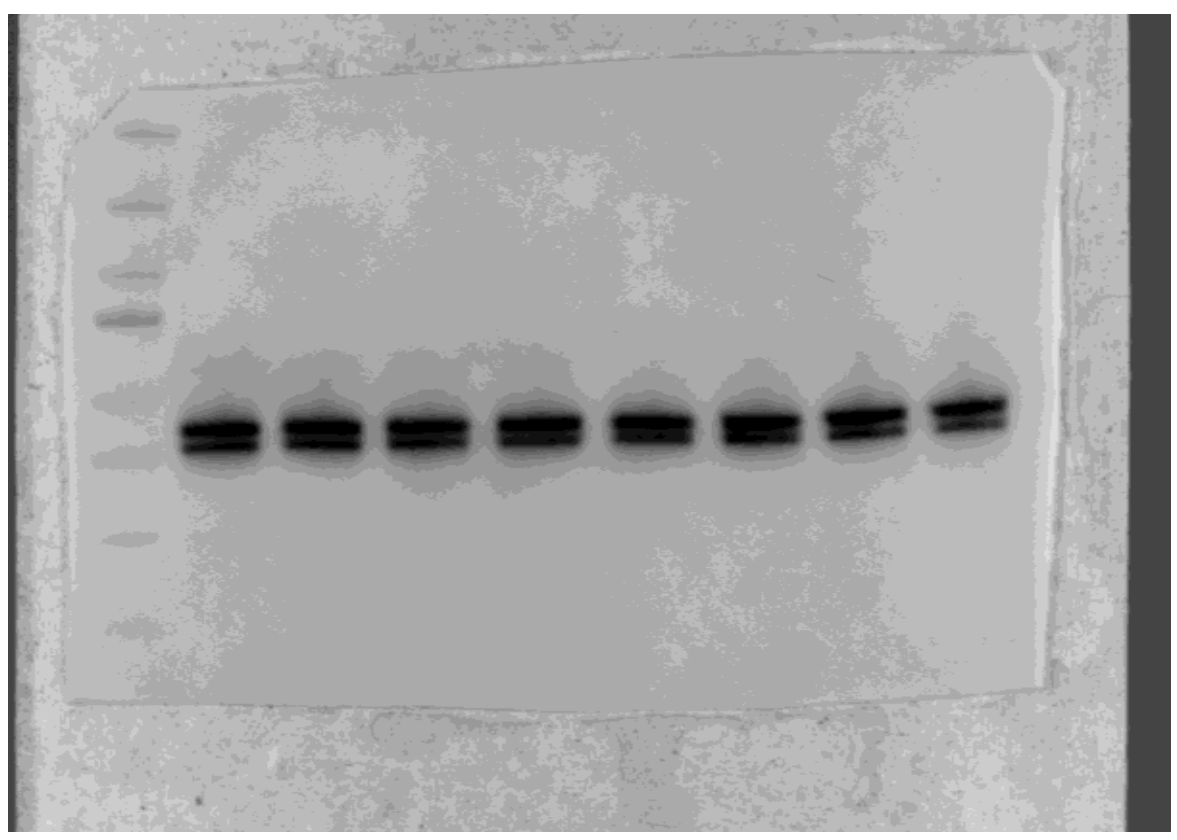

β-actin (42kDa)

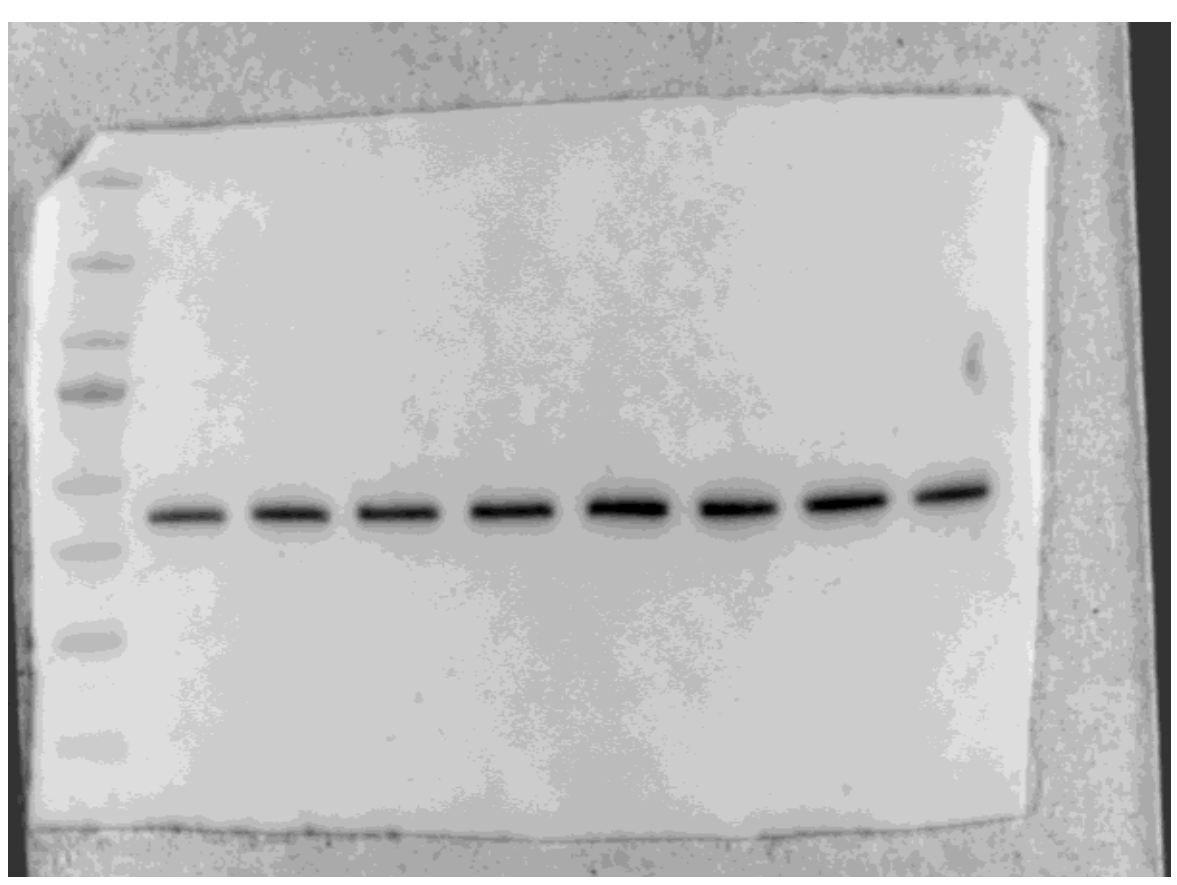

Figure 7E

p-eIF4E (25kDa)

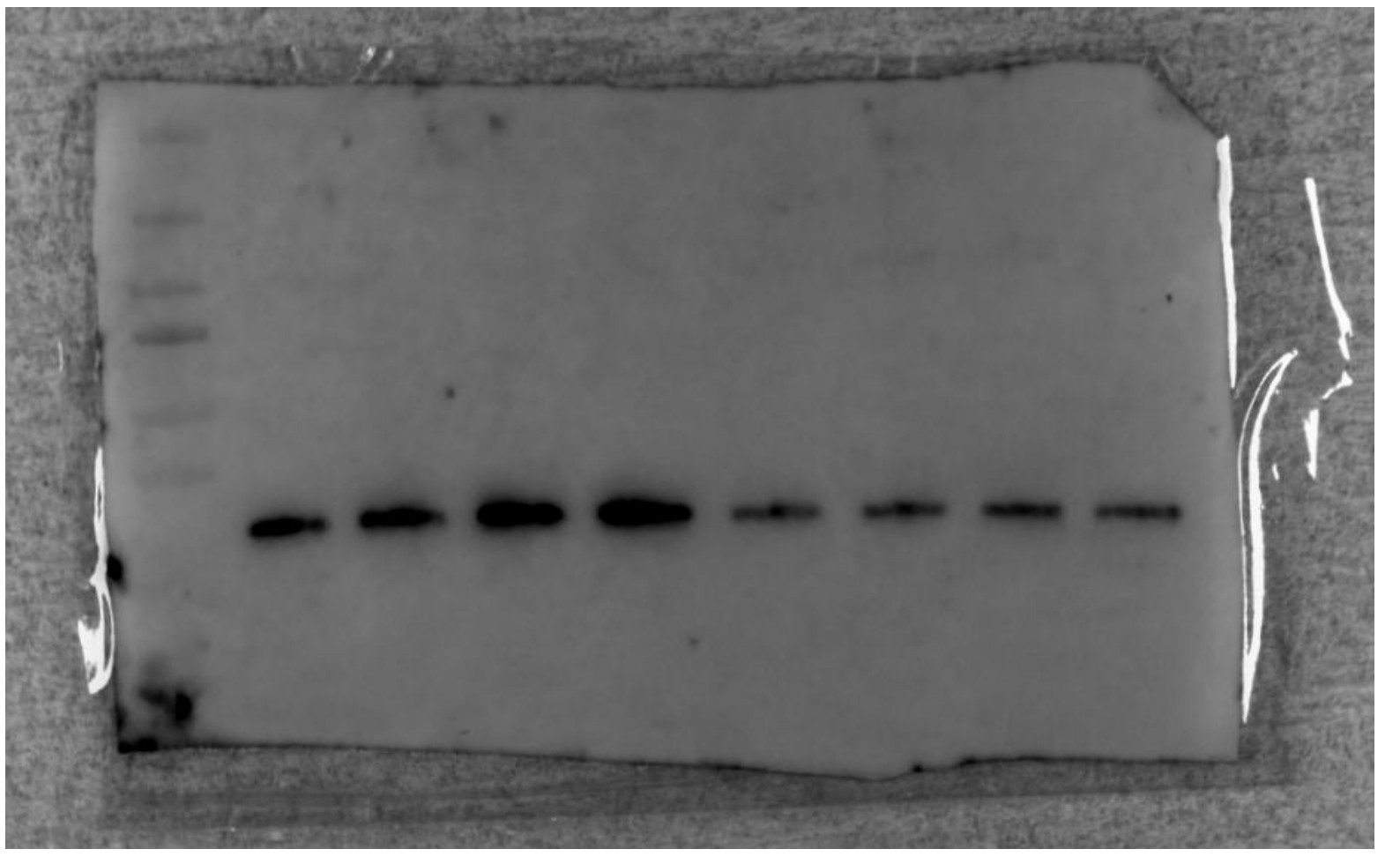

eIF4E (25kDa)

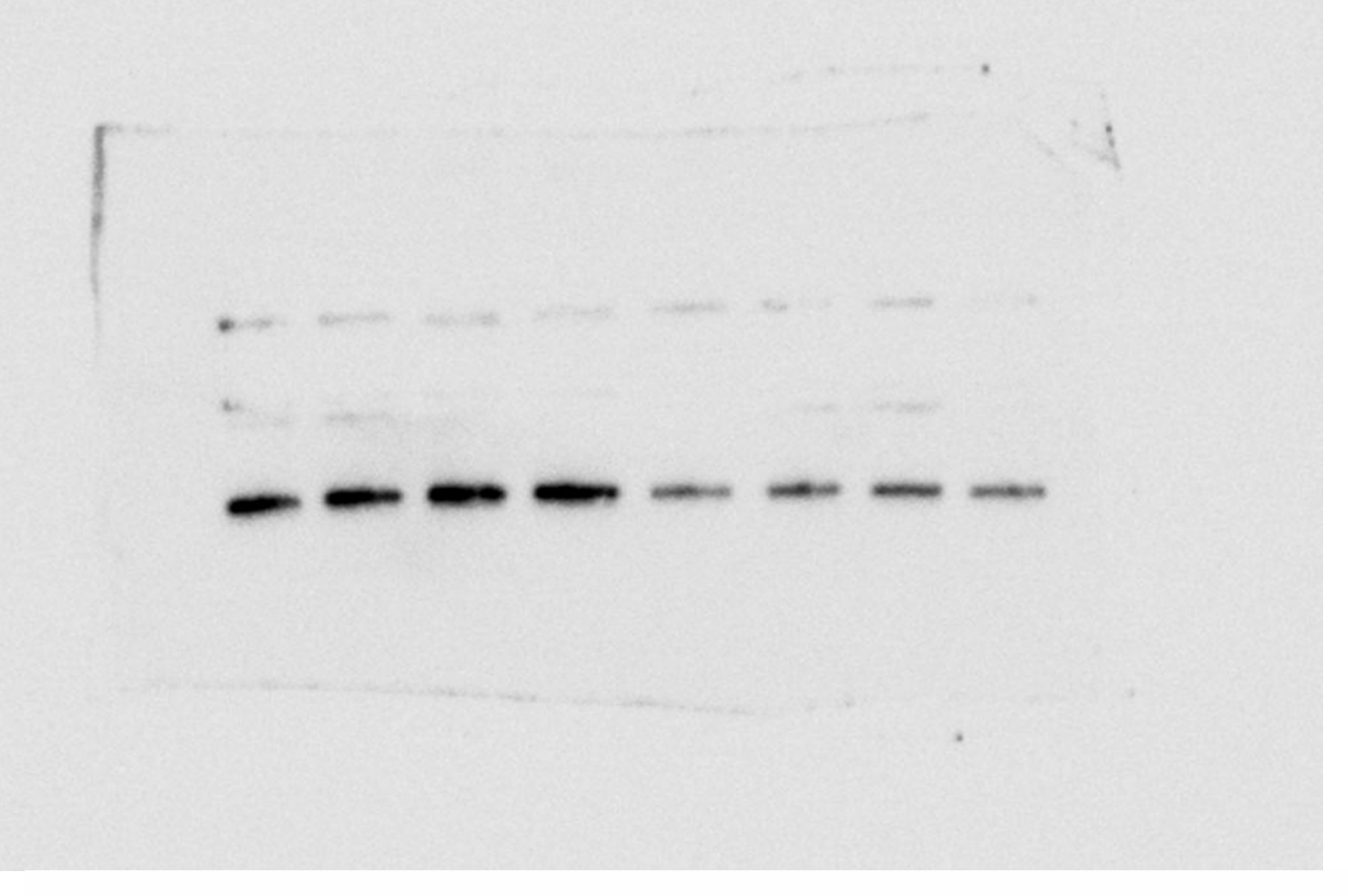

β-actin (42kDa)

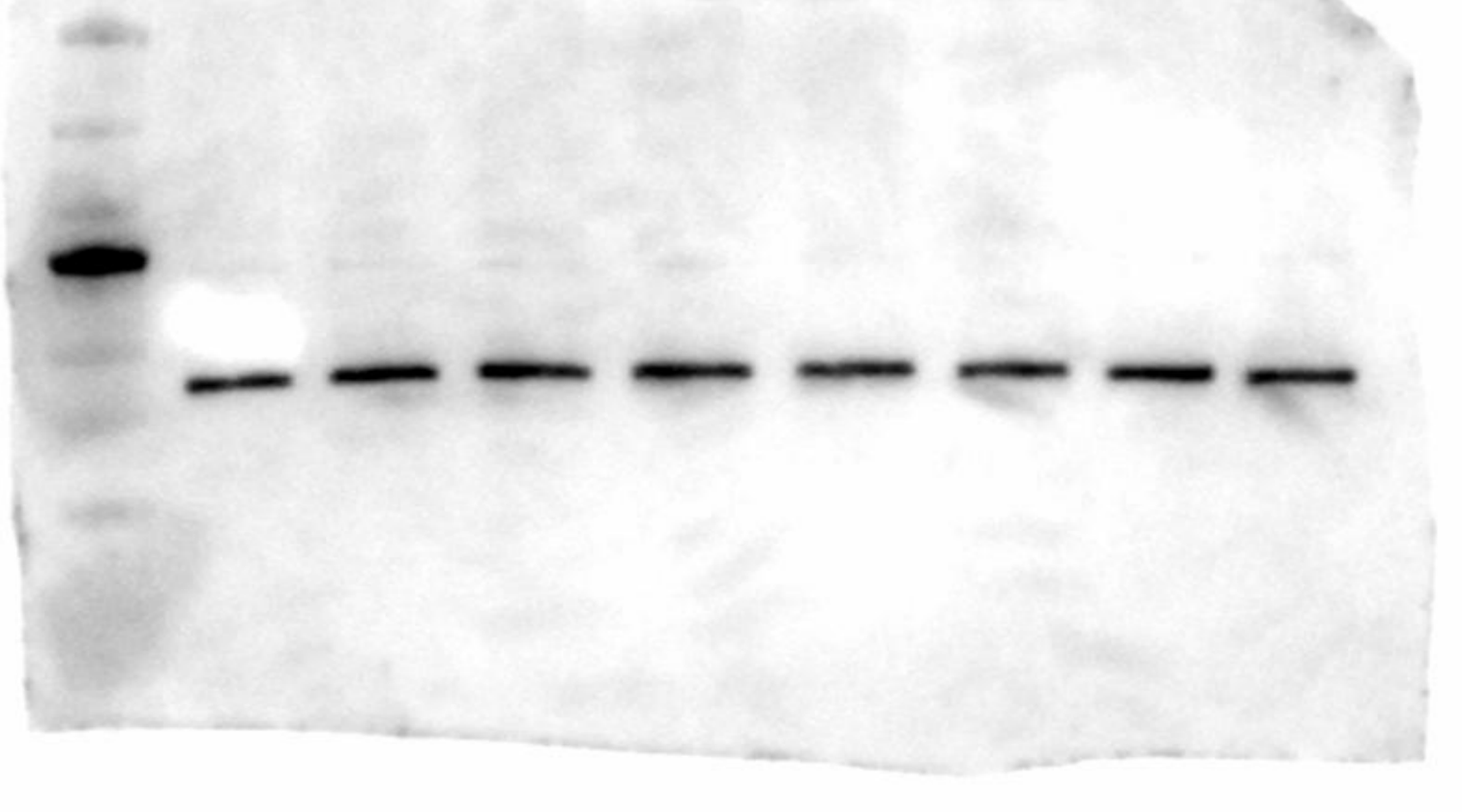

Figure 7F

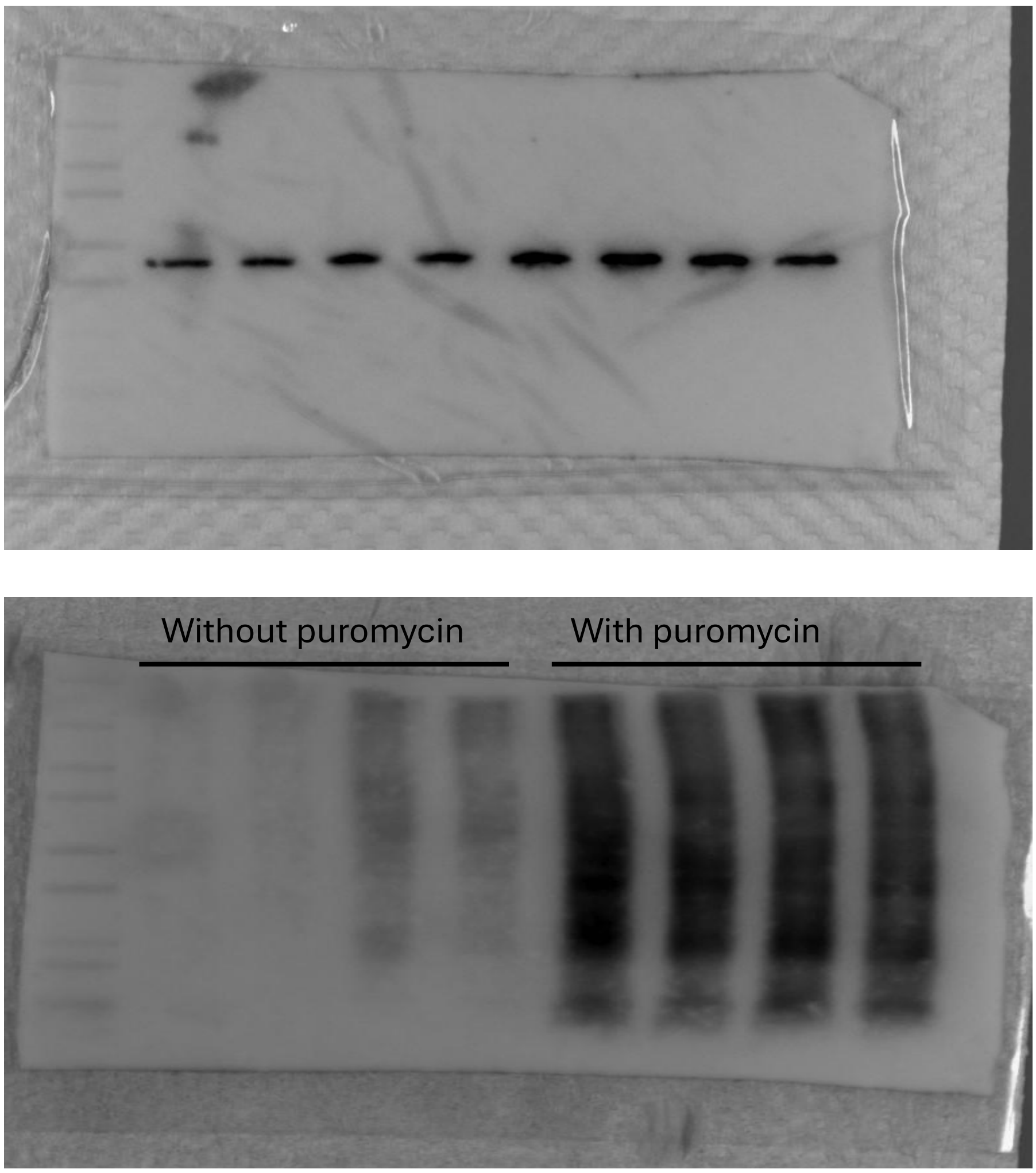

Figure 8A

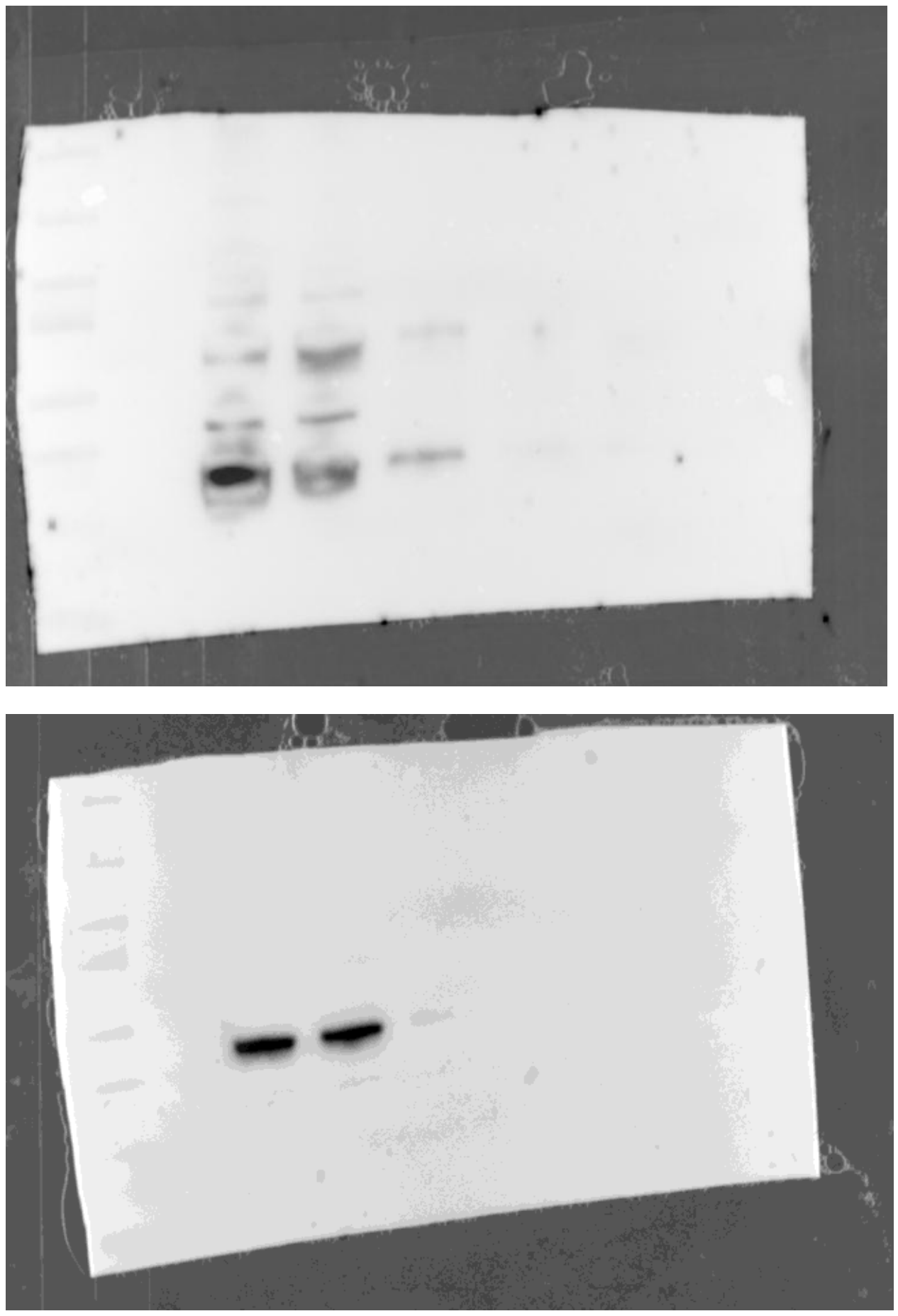

HuR (36kDa)

β-actin (42kDa)

Figure 8E

TXNIP (55kDa)

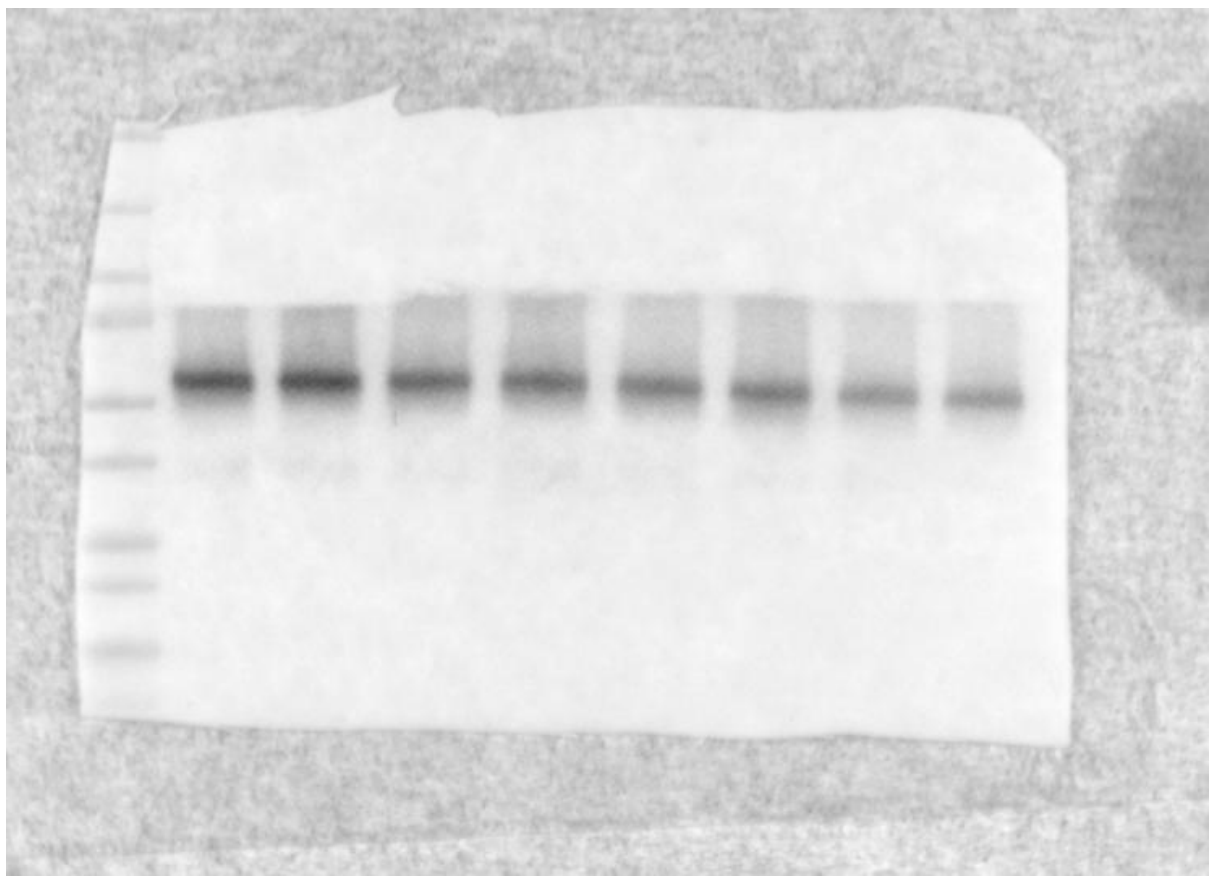

β-actin (42kDa)

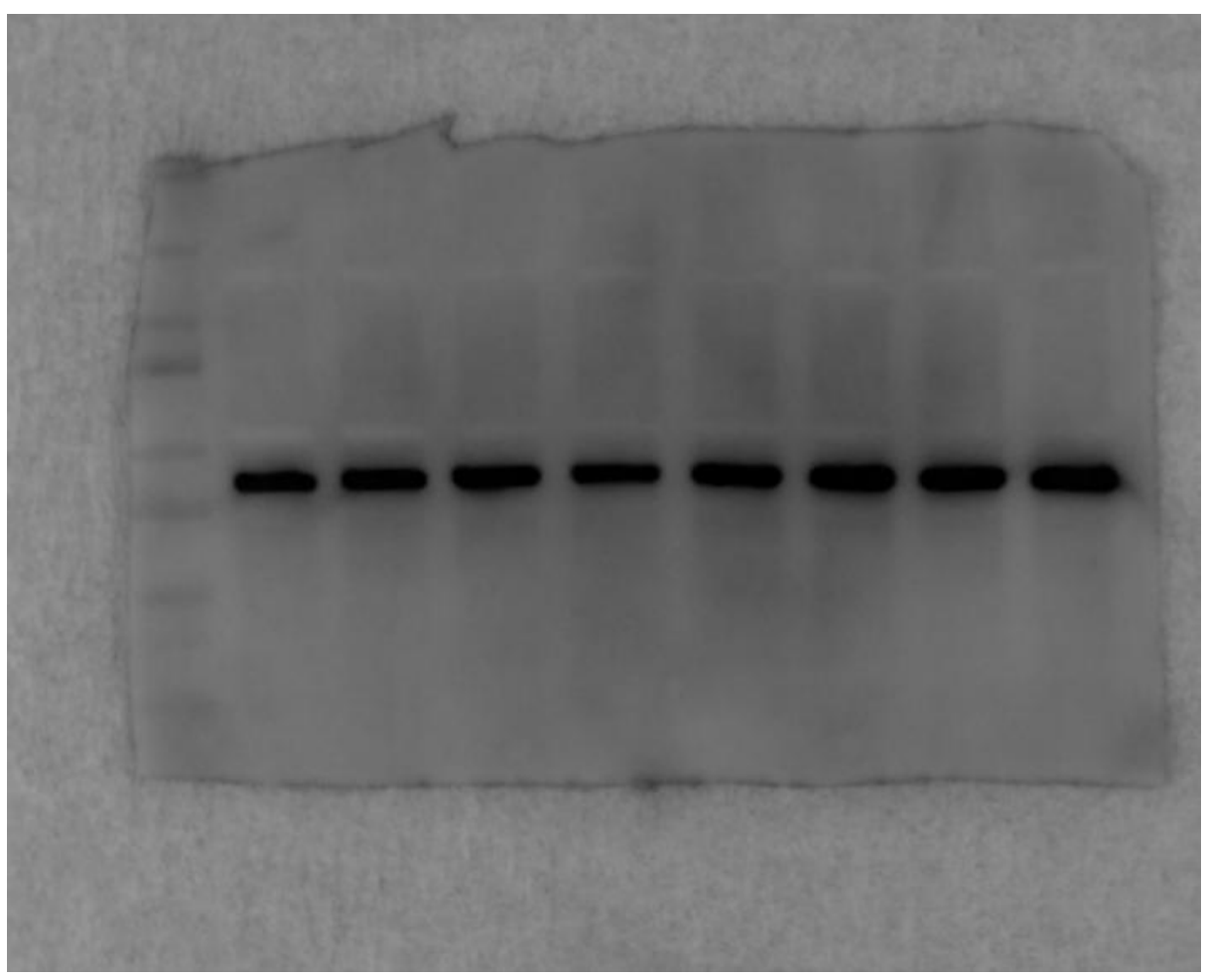

HuR (36kDa)

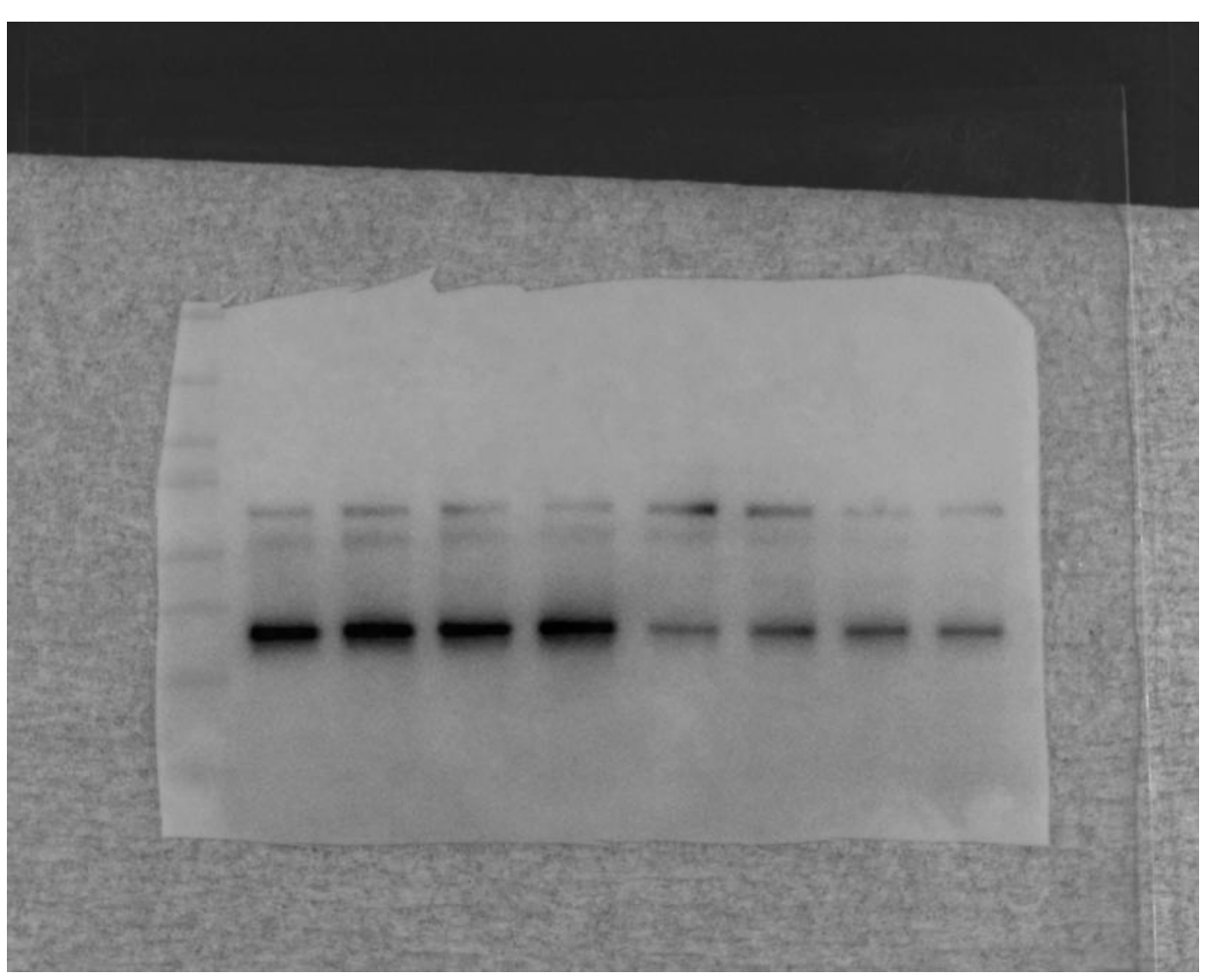

Figure 8G

TXNIP (55kDa)

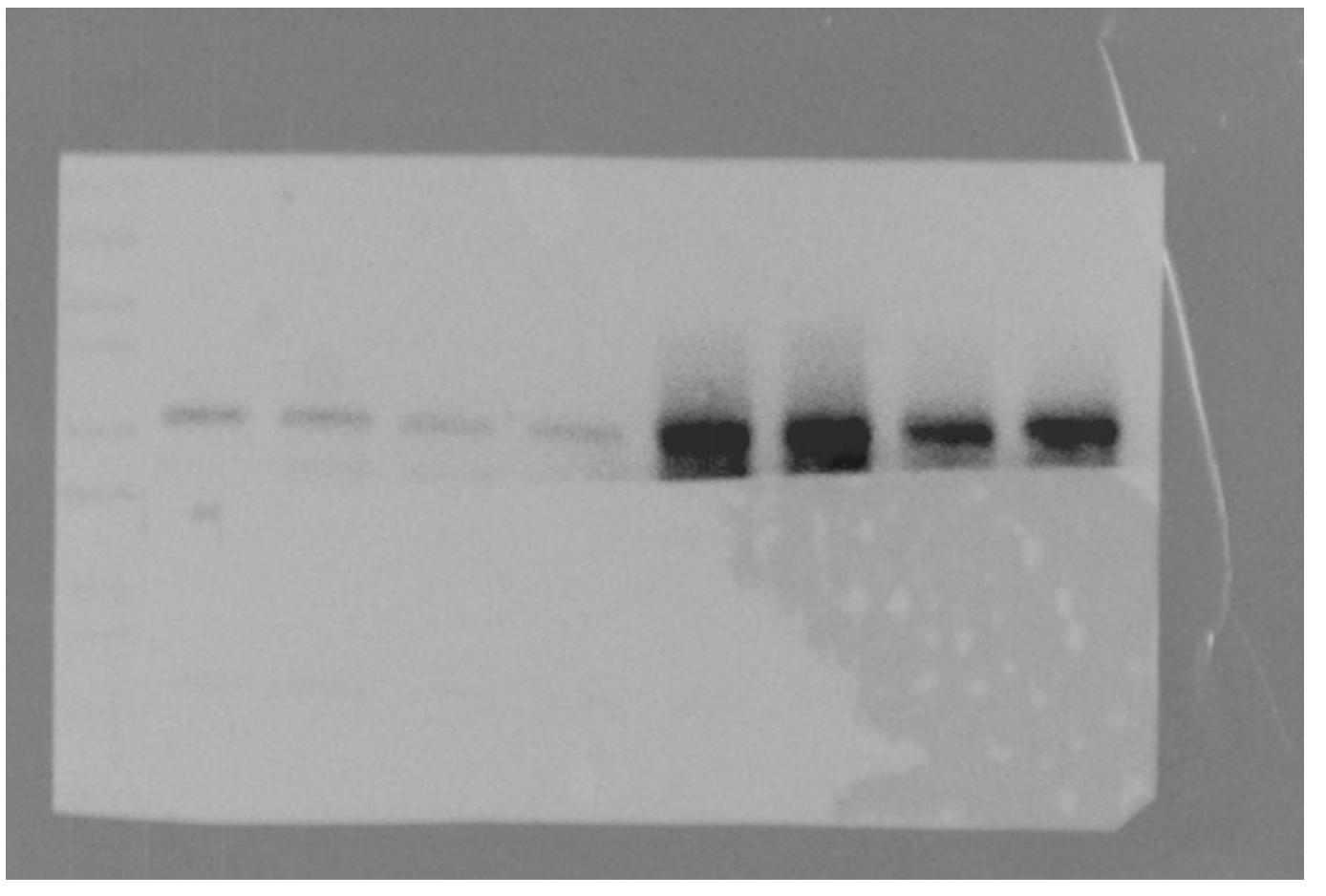

β-actin (42kDa)

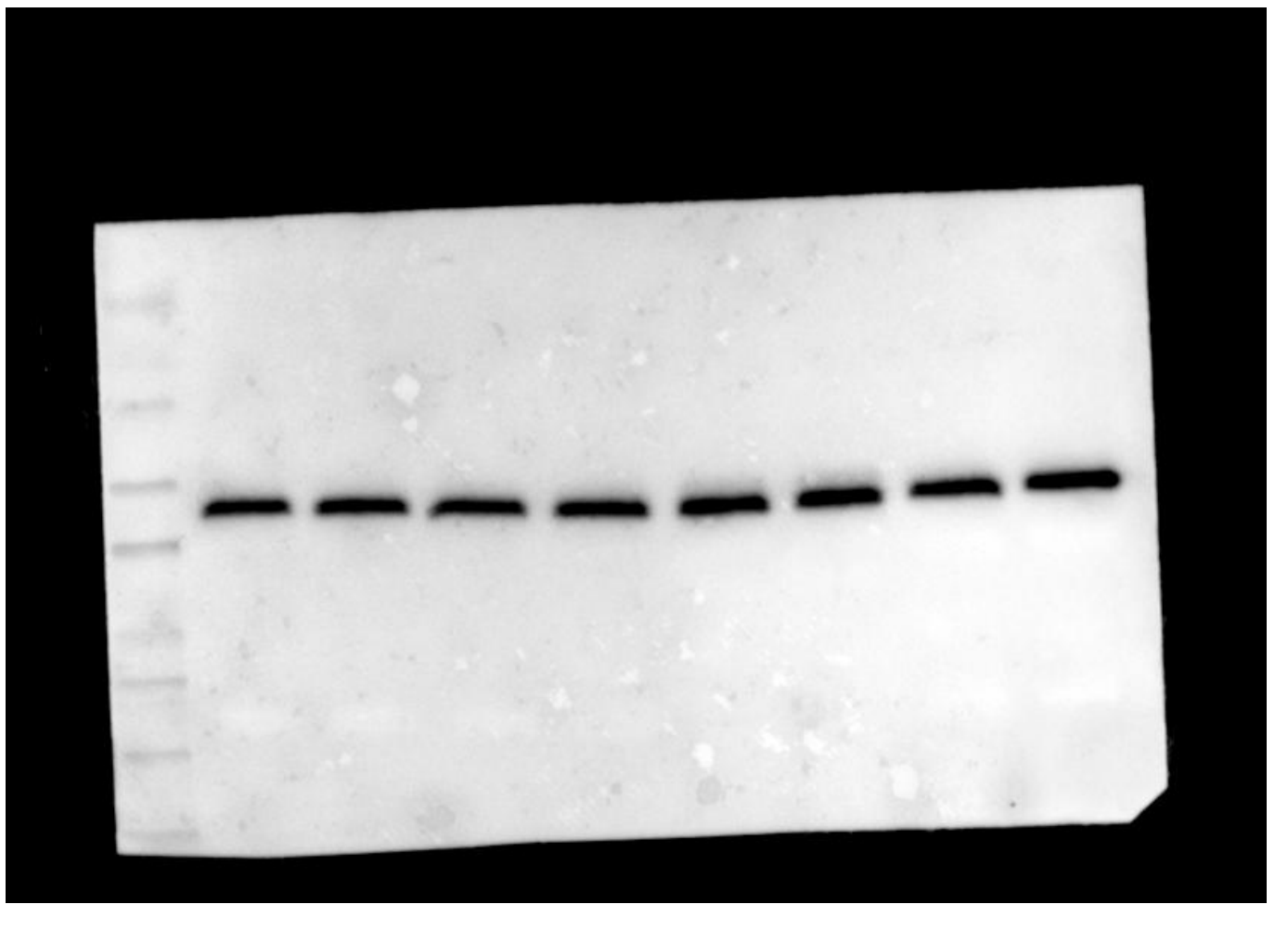

Supplement: Supplementary file 1 [file mmc1.pdf]
